# Common health conditions in childhood and adolescence, school absence, and educational attainment: Mendelian randomization study

**DOI:** 10.1038/s41539-020-00080-6

**Published:** 2021-01-04

**Authors:** Amanda Hughes, Kaitlin H. Wade, Matt Dickson, Frances Rice, Alisha Davies, Neil M. Davies, Laura D. Howe

**Affiliations:** 1 Medical Research Council Integrative Epidemiology Unit, University of Bristol, Bristol, BS8 2BN UK; 2 Bristol Medical School, University of Bristol, Barley House, Oakfield Grove, Bristol, BS8 2BN UK; 3 Institute for Policy Research, University of Bath, Bath, UK; 4 Cardiff University, Cardiff, UK; 5 Public Health Wales, Wales, UK; 6 Department of Public Health and Nursing, K.G. Jebsen Center for Genetic Epidemiology, NTNU, Norwegian University of Science and Technology, Trondheim, Norway

**Keywords:** Education, Human behaviour

## Abstract

Good health is positively related to children’s educational outcomes, but relationships may not be causal. Demonstrating a causal influence would strongly support childhood and adolescent health as important for education policy. We applied genetic causal inference methods to assess the causal relationship of common health conditions at age 10 (primary/elementary school) and 13 (mid-secondary/mid-high school) with educational attainment at 16 and school absence at 14–16. Participants were 6113 children from the Avon Longitudinal Study of Parents and Children (ALSPAC). Exposures were symptoms of attention-deficit hyperactivity disorder (ADHD), autism spectrum disorder (ASD), depression, asthma, migraines and BMI. Genetic liability for these conditions and BMI was indexed by polygenic scores. In non-genetic, multivariate-adjusted models, all health conditions except asthma and migraines were associated with poorer attainment and greater school absence. School absence substantially mediated effects of BMI (39.9% for BMI at 13) and migraines (72.0% at 10), on attainment with more modest mediation for emotional and neurodevelopmental conditions. In genetic models, a unit increase in standardized BMI at 10 predicted a 0.19 S.D. decrease (95% CI: 0.11, 0.28) in attainment at 16, equivalent to around a 1/3 grade lower in all subjects, and 8.7% more school absence (95% CI:1.8%,16.1%). Associations were similar at 13. Genetic liability for ADHD predicted lower attainment but not more absence. Triangulation across multiple approaches supports a causal, negative influence on educational outcomes of BMI and ADHD, but not of ASD, depression, asthma or migraine. Higher BMI in childhood and adolescence may causally impair educational outcomes.

## Introduction

Good health in childhood predicts better educational attainment^1–3^ but associations may not be causal. Less advantaged children have worse health^4^, so associations could be confounded by socioeconomic conditions^5^ or, especially for mental health, reflect reverse causation^6^. But if childhood health does causally influence attainment, it may play an important role in intergenerational transmission of socioeconomic (dis)advantage^7^.

Impact on education of behavioural, emotional and physical health may differ^8^. Attention-deficit hyperactivity disorder (ADHD) predicts lower educational attainment, as measured by test scores and grade repetition^9–11^, including where sibling-fixed effects are used to control for family-level confounding^10^. Evidence is more mixed for depressive symptoms^6,12–16^, which in some studies show a negative association with years of schooling but in others do not. For autism spectum disorder (ASD), evidence suggests substantial heterogeneity in educational outcomes, even after considering variability in IQ^17,18^. For BMI, evidence is mixed. Among longitudinal studies, plausibly less affected by reverse causation, and other causal inference studies, only some support a detrimental impact of overweight/obesity in childhood on educational outcomes^19–22^. There is also mixed evidence for asthma^23–27^ and rarer health conditions^23–25,28,29^. The impact on school absence, and any mediating role of school absence in explaining associations of health with educational attainment, also likely differs by condition^30–32^. A link with school absence is clearer for migraine^26,33^ and depression^34^, than for asthma^23,27,31^, ADHD^24,35^, ASD^24,36^ or obesity^37–40^.

Approaches have been developed to circumvent confounding and reverse causation by using genetic variants associated with health conditions as proxies or instrumental variables. Genetic variants associated with health conditions are, conditional on certain assumptions, assigned randomly at conception and cannot be influenced by later environmental influences or health^41^. Methods using genetic variants are therefore unaffected by classical kinds of bias or reverse causation which affect traditional observational study designs, and for this reason are often compared to a randomized controlled trial^42^. These approaches support a causal influence on educational attainment of ADHD^9,43^, but are inconclusive for body weight^44–46^. Results for ASD are null^43^, or point to existence of high-functioning subgroups^47^. One study has examined depression, reporting null results^48^.

The aim of this study was to assess the causal influence of common health conditions in childhood and adolescence on educational attainment, on school absence, and the extent to which school absence mediates health-attainment associations. We applied genetic methods to the impact of six aspects of childhood and adolescent health on educational attainment and on school absence: ADHD, ASD, depressive symptoms, BMI, asthma, and migraines, in an English birth cohort^49^. We considered health conditions which are prevalent enough to be of considerable importance in current child and adolescent public health, and which have known genetic markers, to allow application of genetic methods. To avoid the confounding and reverse causation, which have affected previous investigation, we use polygenic scores (PGSs) capturing genetic liability for health conditions. We explore associations of health with educational attainment, associations with absenteeism, and mediation of associations with attainment by absenteeism. In all cases we avoid recall bias by using linked records for school absence and educational attainment. We consider health at age 10 and 13, to examine whether influence of health on attainment differs with age. Finally, we conducted two-sample Mendelian randomization for influence of the same conditions on educational attainment in independent adult samples.

## Results

### Socioeconomic background, GCSEs, and school absence

Reflecting the regional nature of the cohort, the analytic sample showed differences from national averages, for example in a slightly higher maternal age (28.3 years, compared to 27.7 nationally in 1991). Participants in the analytic sample had a higher average GCSE capped score (around 332 points) than the national average at the time (308 points), consistent with differences previously described for the cohort as a whole^49^. Within the analytic sample, GCSEs and school absence (Table 1) varied by gender and socioeconomic background (Supplementary Table 6). GCSE points scores were higher for girls than boys: 345.6 (95% CI: 338.7, 352.5) compared to 319 (95% CI: 311.0, 327.1), as was school absence: 7.8% (95% CI: 7.2%, 8.4%) compared to 7.2% (95% CI: 6.6%, 7.7%). Maternal education was associated positively with attainment, and negatively with school absence. Children whose mothers had a degree had an average GCSE point score of 392.9 (95% CI: 387.0, 398.9) and school absence of 6.1% (95% CI: 5.0%, 7.3%). For children whose mothers had the least qualifications, average GCSE points score was 275.7 (95% CI: 266.9, 284.5) and school absence 9.9% (95% CI: 9.0%, 10.7%). GCSE points scores were negatively associated with school absence. Adjusting for gender, an increase in absence corresponding to an extra day/year was associated with −2.6 (−3.2,−2.1) fewer GCSE points.

**Table 1:** Descriptive characteristics of analytic sample (*N* = 6113)^a^.

Continuous variables	Mean	SD	Range
Maternal age	28.27	4.68	15–44.1
SDQ hyperactivity score^b^ at 10 (114 months)	3.14	2.23	0–10
SDQ hyperactivity score^b^ at 13 (156 months)	3.23	2.21	0–10
MFQ score^c^ at 10 (127 months)	4.32	3.60	0–22.07
MFQ score^c^ at 13 (154 months)	4.24	3.94	0–24
SCDC^d^ scored at 10 (120 months)	2.91	3.79	0–24
SCDC^d^ scored at 13 (156 months)	3.29	3.97	0–24
BMI *z*-score^e^ at 10 (127 months)	0.34	1.16	−3.65 to 4.26
BMI *z*-score^e^ at 13 (154 months)	0.41	1.20	−3.83 to 4.55
GCSE capped points score	332.34	87.36	0–540
	Median	IQR	Range
Percent of sessions absent, year 11 (age 15–16)	5.53	7.28	0–98.59
Percent of sessions absent, year 10 (age 14–15)	4.83	6.71	0–90.39
Percent of sessions absent, key stage 4 (age 14–16)	5.42	6.34	0–79.29
Categorical variables	Category		%
Gender	Male		49.99
	Female		50.01
Maternal educational qualifications	CSE or less		16.77
	vocational		9.49
	O level		35.94
	A level		24.33
	Degree		13.47
Maternal parity at child’s birth	0		44.9
	1		36.15
	2		14.00
	3+		4.94
Migraines at 10	No		95.03
	Yes		4.97
Asthma at 10 (128 months)	No		87.77
	Yes		12.23
Asthma at 13 (157 months)	No		87.99
	Yes		12.01
School type^f^ at key stage 4 (age 14–16)	Mainstream state		93.13
	Independent		5.45
	Other		1.42

^a^Analysis was restricted to unrelated ALSPAC participants with genetic data and linked GCSE records. Missing data in covariates, exposures and school absence was imputed using multiple imputation by chained equations.

^b^SDQ = Strengths and Difficulties Questionnaire, for ADHD symptoms.

^c^MFQ = Mood and Feelings Questionnaire, for depressive symptoms.

^d^SCDC = Social Communication Disorder Checklist, for autistic social traits.

^e^Using 1990 UK Growth Reference. Values represent standard deviation difference from age-specific and gender-specific reference mean.

^f^Mainstream state schools: community, voluntary controlled or aided, foundation, city technology college, academy. Other schools: community special, pupil referral unit, further education college.

### Phenotypic models: health, GCSEs and school absence

In phenotypic models (Table 2), all aspects of child and adolescent health were associated with GCSE points score except migraines and asthma. All were associated with school absence (Table 2). Depressive symptoms at 10 showed a considerably stronger association with GCSEs than depressive symptoms at 13 (GCSE points scores: −16.4 (95% CI: −19.7, −13.1) compared to −6.8 (95% CI: −9.9, −3.8) per SD MFQ score). Otherwise, associations did not differ substantially by age. In mediation analyses, associations between all aspects of health and educational attainment were mediated by school absence except for asthma (Table 3). Percent of associations mediated was lowest for ADHD (7.1% at age 10, 8.4% at 13) and highest for BMI (39.9% at age 10, 32.6% at 13) and migraine (72.0% at age 10). (Table 3). Results were similar restricting to participants in mainstream state schools. For other school types, small numbers of participants led to imprecise estimates (Supplementary Tables 7 and 8).

**Table 2:** Phenotypic associations of health conditions with educational attainment at 16 and school absence at 14–16 (*N* = 6113).

	GCSE points score^a^		School absence at age 14–16^b^	
	Beta	CI	% increase	CI
*Health conditions at age 10*				
Standardized values of SDQ-HI score^c^	−25.58	−28.42, −22.73	6.08	3.51, 8.72
Standardized values of MFQ score^d^	−16.39	−19.67, −13.10	6.02	3.24, 8.88
Standardized values of SCDC score^e^	−20.73	−24.09, −17.37	9.34	6.39, 12.37
Migraines at age 10	−6.58	−19.12, 5.97	15.42	3.25, 29.03
Asthma in past 12 months	2.06	−5.01, 9.14	12.15	4.48, 20.39
BMI *z*-score^f^	−2.30	−4.11, −0.49	2.82	0.96, 4.71
*Health conditions at age 13*				
Standardized values of SDQ-HI score^c^	−28.75	−31.56, −25.95	8.58	5.89, 11.34
Standardized values of MFQ score^d^	−6.83	−9.90, −3.76	7.10	4.66, 9.61
Standardized values of SCDC score^e^	−25.23	−28.84, −21.62	12.96	9.62, 16.4
Asthma in past 12 months	−1.27	−9.18, 6.63	10.60	2.67, 19.14
BMI *z*-score^f^	−3.99	−5.88, −2.11	3.99	2.15, 5.86

^a^Coefficients represent change in GCSE capped points score adjusted for gender, maternal education, maternal housing tenure, maternal age, maternal parity, whether smoked in pregnancy.

^b^Coefficients represent proportional change in absenteeism (0% = no change) with presence of the health condition, or per SD increase in continuous exposures.

^c^SDQ-HI: Strengths and Difficulties Questionnaire hyperactivity subscale.

^d^Mood and Feelings Questionnaire.

^e^Social Communication Disorders Checklist.

^f^Based on 1990 UK Growth Reference, values represent SD difference from age-specific and gender-specific reference mean.

**Table 3:** Mediation by absenteeism at age 14–16 of associations of health conditions with educational attainment at 16.

Exposure	Age	Pathway	Beta^a^	LCI	UCI	*p*	% mediated
SDQ-HI score^b^ for ADHD	10	Direct	−23.77	−26.13	−21.41	<0.001	7.05
		Indirect	−1.80	−2.84	−0.77	<0.001	
		Total	−25.58	−28.02	−23.13	<0.001	
MFQ score^c^ for depressive symptoms	10	Direct	−14.54	−17.22	−11.87	<0.001	11.26
		Indirect	−1.84	−3.02	−0.67	0.002	
		Total	−16.39	−19.18	−13.59	<0.001	
SCDC score^d^ for autistic social traits	10	Direct	−18.01	−20.62	−15.39	<0.001	13.13
		Indirect	−2.72	−3.93	−1.51	<0.001	
		Total	−20.73	−23.36	−18.10	<0.001	
Migraines at 10	10	Direct	−1.84	−10.95	7.26	0.69	71.97
		Indirect	−4.73	−8.28	−1.19	0.01	
		Total	−6.58	−16.11	2.96	0.18	
Asthma in past 12 months	10	Direct	5.87	−0.30	12.05	0.06	−184.97
		Indirect	−3.81	−6.34	−1.28	0.003	
		Total	2.06	−4.39	8.51	0.53	
BMI *z*-score^f^	10	Direct	−1.38	−3.44	0.67	0.19	39.87
		Indirect	−0.92	−1.89	0.05	0.06	
		Total	−2.30	−4.42	−0.18	0.03	
SDQ-HI score^b^ for ADHD	13	Direct	−26.35	−28.71	−23.98	<0.001	8.37
		Indirect	−2.41	−3.50	−1.31	<0.001	
		Total	−28.75	−31.14	−26.37	<0.001	
MFQ score^c^ for depressive symptoms	13	Direct	−4.60	−7.28	−1.92	<0.001	32.63
		Indirect	−2.23	−3.43	−1.03	<0.001	
		Total	−6.83	−9.61	−4.05	<0.001	
SCDC score^d^ for autistic social traits	13	Direct	−21.73	−24.50	−18.96	<0.001	13.87
		Indirect	−3.50	−4.80	−2.20	<0.001	
		Total	−25.23	−28.00	−22.47	<0.001	
Asthma in past 12 months	13	Direct	2.07	−4.28	8.41	0.52	262.50
		Indirect	−3.34	−5.93	−0.75	0.01	
		Total	−1.27	−7.87	5.32	0.71	
BMI *z*-score^f^	13	Direct	−2.71	−4.78	−0.65	0.01	32.08
		Indirect	−1.28	−2.24	−0.32	0.01	
		Total	−3.99	−6.12	−1.87	<0.001	

^a^For binary exposures, coefficients represent change associated with presence vs. absence of the health condition. For continuous exposures, coefficients represent change associated with a 1 standard-deviation increase from sample mean.

^b^SDQ-HI: Strengths and Difficulties Questionnaire hyperactivity subscale.

^c^MFQ: Mood and Feelings Questionnaire.

^d^SCDC: Social Communication Disorders Checklist.

^f^Based on 1990 UK Growth Reference, values represent SD difference from age-specific and gender-specific reference mean.

### Genetic models: health, GCSEs and school absence

Predictive power of the PGSs varied considerably. The proportion of variance explained by the PGS (*R*^2^ or pseudo-*R*^2^ for binary exposures) was 7.6% and 7.9% for BMI at 10–11 and 13, respectively, but <1% for ADHD, migraine and asthma, and <0.1% for ASD and depressive symptoms (Supplementary Table 9). Tests for instrument strength (Supplementary Table 9) confirmed only the BMI PGS could be used as an instrumental variable.

For ADHD and BMI, higher values of the PGS predicted lower GCSE points (Table 4). Each SD increase in the PGS for ADHD corresponded to a decrease of 2.70 (95% CI: −4.83, −0.58) GCSE points. A one SD increase in the BMI PGS corresponded to a decrease of 5.37 (95% CI: −7.78, −2.96) GCSE points and a 2.72% (95% CI: 0.57, 4.91) increase in school absence. Using the BMI PGS as an instrumental variable showed that, for each unit increase in BMI *z*-score at age 10, GCSE points scores were 16.79 lower (95% CI: −24.38, −9.19) and absences were 8.73% greater (95% CI: 1.82, 16.12). For each unit increase in age-standardized and gender-standardized BMI at age 13, GCSE points scores were 15.90 lower (95% CI: −23.09, −8.72) and absences were 8.25% greater (95% CI: 1.75, 15.17).

**Table 4:** Association of polygenic scores with educational attainment at 16 and school absence at age 14–16^a^.

	GCSE points	CI	Absences: % increase	CI
Standardized values of ADHD PGS	−2.70	−4.83, −0.58	−0.24	−2.13, 1.69
Standardized values of depression PGS	0.75	−1.75, 3.25	−0.76	−2.80, 1.32
Standardized values of ASD PGS	−1.78	−3.84, 0.28	−0.41	−2.43, 1.65
Standardized values of migraine PGS	−0.93	−3.04, 1.18	1.38	−0.59, 3.39
Standardized values of asthma PGS	−0.66	−2.80, 1.48	1.18	−0.67, 3.07
Standardized values of BMI PGS	−5.37	−7.78, −2.96	2.72	0.57, 4.91

^a^*N* = 6113. Adjusted for gender and PC1–PC20. GCSE points score: range 0–540, mean 332.3, SD 87.4. Coefficients for absences represent proportional change in absenteeism (0% = no change) per SD increase in the polygenic score.

Results were again similar restricting to participants in mainstream state schools, with estimates for other school types imprecise (Supplementary Table 10). Tests of instrument validity applying two-sample methodology in ALSPAC (Supplementary Table 11) were consistent with main results, and there was no evidence of bias due to pleiotropy for associations with GCSEs. Although there was evidence of pleiotropy for effect of BMI on absenteeism (MR-Egger intercept 0.004, *p* = 0.02), additional SNP-specific checks^50^ could not identify particular SNPs responsible (Supplementary Table 12). Results did not differ using a BMI PGS excluding 24 SNPs identified as outliers in two-sample MR (Supplementary Table 13).

Results of two-sample MR analyses using published GWAS for educational attainment (years of schooling) were broadly consistent with results from ALSPAC (Table 5). Previous two-sample MR analysis reported evidence of an effect of ADHD^43^ but not ASD^43^ or depression^48^. Our analyses supported an influence of BMI, with a one-unit increase in BMI associated with 0.16 (*p* < 0.001) and 0.11 (*p* < 0.001) fewer years of schooling in IVW and weighted median models, respectively. These models also support a negative influence of asthma on years of schooling (−0.02, *p* < 0.001 and −0.02, *p* = 0.01, respectively). There was little evidence of a causal impact of migraine. For BMI, the MR-Egger constant (−0.002, *p* < 0.001) indicated an influence of pleiotropy. Outliers were therefore identified by comparing SNP-specific estimates with the overall IVW estimate, and analyses repeated with these SNPs excluded. This did not change conclusions (Supplementary Table 14). We checked associations of these SNPs with other phenotypes but none stood out as clear confounders (Supplementary Table 15).

**Table 5:** Results from two-sample summary-level Mendelian randomization^a^.

Exposure and outcome GWAS	Method	*N* SNPs	Beta^b^	SE	*p*
Migraine^80^ Years of schooling^63^	Inverse variance weighted	29	−0.002	0.011	0.824
	Weighted median	29	0.016	0.010	0.090
	Weighted mode	29	0.018	0.011	0.113
	MR Egger	29	−0.014	0.029	0.619
	MR Egger—Intercept	29	0.001	0.002	0.654
Asthma^79^ Years of schooling^63^	Inverse variance weighted	8	−0.023	0.005	<0.0001
	Weighted median	8	−0.018	0.007	0.009
	Weighted mode	8	−0.013	0.014	0.396
	MR Egger	8	−0.042	0.038	0.313
	MR Egger—Intercept	8	0.003	0.005	0.632
BMI^68^ Years of schooling^85^	Inverse variance weighted	945	−0.158	0.010	<0.0001
	Weighted median	945	−0.109	0.012	<0.0001
	Weighted mode	945	−0.032	0.031	0.306
	MR Egger	945	−0.042	0.028	0.137
	MR Egger—Intercept	945	−0.002	0.000	0.000

^a^Conducted in MR Base with the TwoSampleMR package. Details of GWAS used in Supplementary Table 3.

^b^Betas from Two-Sample MR represent change in the outcome (years of schooling) per unit increase in BMI, or per unit increase in log-odds of having asthma or migraine.

## Discussion

In phenotypic analysis, all aspects of poorer health at ages 10 and 13 predicted greater school absences at ages 14–16, and all except asthma and migraines predicted lower educational attainment at 16. Depressive symptoms at 10 were more strongly associated with GCSEs than were depressive symptoms at 13. This supports previous work indicating that emotional health around the time of transition from primary school to secondary school may be important for later educational outcomes^51^. For ADHD, ASD, and depressive symptoms, we explored the effect of an incremental increase in symptoms rather than a diagnosis, and relationships of diagnoses with educational outcomes may differ. Lack of associations of asthma and migraine with GCSEs may reflect binary measures unable to capture the full range of symptoms, or misclassification of exposures as diagnosis can occur later. Results of mediation analysis are consistent with a recent UK study^52^ which reported substantial mediation of by parent-reported long-term absence and truancy of the impact of long-term health conditions and of mental health on educational attainment. Our findings indicate such results do not only reflect recall bias and add to current knowledge by showing that school absence additionally mediates the association between BMI and educational attainment. However, phenotypic associations are vulnerable to reverse causation and residual confounding by family-level and individual-level characteristics not captured by covariates. For this reason, we applied genetic causal inference approaches to the relationships.

Consistent with previous work in ALSPAC^9^, genetic liability for ADHD was associated with worse GCSEs, but it was not associated with greater school absence. This is consistent with extensive evidence for the influence of other factors on academic attainment, including how well a child ‘fits’ with the expectations of the school environment, teacher views and attitudes, and bullying by peers^53^. Genetic liability for higher BMI was associated with both attainment and school absence, and using the BMI PGS as an instrument supported these findings. MR analyses using ALSPAC found some evidence of pleiotropy in BMI-absenteeism associations, although no individually pleiotropic SNPs were identified. Mechanisms should be explored in samples large enough to investigate SNP-specific pathways. Two-sample summary-level Mendelian randomization based on older individuals also supported a negative influence of higher BMI on educational attainment. These results indicate that, for BMI, associations with attainment and school absence do not simply reflect confounding. Results from previous observational studies have been mixed, which may reflect heterogeneity in the quality of studies^20^. Previous genetic causal inference work in unrelated adults has suggested that higher BMI reduces likelihood of having a university degree^54^. Our results are consistent with those findings and indicate that any negative impact of BMI on educational attainment may begin long before university. Recent work using genetic data on adult sibling pairs has investigated the influence of family-level factors which could bias results of MR studies using samples of unrelated individuals, for example the influence of parental genotype on offspring phenotype via environmental pathways, or assortative mating^46^. This study found that, when these biases are accounted for, the estimated impact of BMI on educational attainment attenuates. The results in the current study of BMI with educational outcomes may therefore partly be due to assortative mating or parental effects. Investigating these mechanisms requires genetic data on large numbers of related individuals (e.g. siblings or parent–offspring trios), not available in the current study.

Causal mechanisms besides absenteeism linking BMI to attainment are likely complex. Cross-sectional research has suggested negative neurocognitive correlates of obesity, but has not established causal influence^55^ and evidence from longitudinal studies is less clear^19^. Socially mediated processes by which weight could influence educational outcomes involve weight bias by teachers^56^ and bullying by peers^57^. Further work using genetic and qualitative approaches will be required to unpick these mechanisms. That IV coefficients were larger than OLS coefficients for the (negative) influence of BMI on GCSEs may point to an offsetting mechanism causing suppression of effects in observational models. In UK children, both thinness and obesity are associated with deprivation^58^, so a nonlinear relationship between BMI and socioeconomic hardship could bias downwards estimates of the impact of BMI on attainment. IV estimates could also be inflated due to family-level processes, such as the influence of parents’ genotype on offspring via environmental pathways^59^, which may bias MR estimates based on samples of unrelated individuals^46^.

Negative associations of depressive symptoms at age 12^60^, ADHD symptoms in preschool^61^ and obesity at 11 and 16 with GCSEs^22^ have been previously shown in ALSPAC. Our results are consistent with a causal interpretation of the latter two. They are also consistent with studies into educational impact of ADHD and depression using within-sibling comparison^10,12^, which addresses confounding at the level of the family, but not the individual. Two-sample MR suggested an additional influence of asthma on educational attainment. This was observed in a recent study using UK Biobank, where genetically instrumented asthma corresponded to a 17% lower probability (CI: −25.3% to −8.7%) of holding a degree^62^. Since in the two-sample analysis the outcome was measured in a substantially older population^63^, the discrepancy with ALSPAC could reflect better treatment available to younger cohorts, or asthma diagnoses made in adulthood.

A key strength of this study is triangulation across several methodological approaches to investigate if associations are causal. A limitation concerns the differential strength of the genetic instruments. For ADHD, ASD and depression, the low proportion of variance in the phenotype explained by the PGS limited the degree to which genetic methods could be meaningfully applied. For ASD, a more lenient threshold was required for SNPs included in the PGS. Thus, genetic results for ASD and depressive symptoms should not be interpreted as evidence of no effect. Rather, associations may become clearer as the genetics of these conditions becomes better understood. Migraine could only be examined at age 10, not 13, where effects may be greater. Recent evidence points to bias due to family-level processes in genetic studies of BMI^46^. Such effects could have influenced results, potentially overestimating BMI’s causal influence on attainment. Work using genetic data on related family members will be required to investigate this further. ALSPAC is not a national survey, and over-representation of affluent groups and young people with comparatively high educational attainment^49^ may limit generalizability. Analysis was restricted to individuals of European ancestry, and results may not be generalizable to other groups. A major strength was use of linked records for educational attainment and school absence, meaning associations were not influenced by recall bias. A limitation is that absence data was restricted to age 14–16, but absence earlier in school may differently affect attainment.

Our results add to the evidence for the importance of health for educational outcomes. They indicate that children and adolescents with a high BMI, and those affected by ADHD, are at particular risk of not fulfilling their educational potential. They may therefore benefit from extra support. Difficulties experienced by children with ADHD result from an interaction or a poor ‘fit’ of the child with a school environment in which neurodiversity is not able to thrive, for instance, where large class sizes are the norm^53^, and teachers are stressed and under pressure^64^. Given this complexity, a more nuanced approach at the school level may be necessary rather than directly targeting the affected children themselves. With a number of possible social and biological mechanisms linking BMI to educational outcomes, including the psychological effects of weight-based stigma or bullying victimization^57,65^, this may also be the case for BMI.

In an English cohort born in the early 1990s, analyses supported a negative, causal influence of high BMI on educational attainment and school absence. Mediation analysis supported mediation by school absence for BMI and for ADHD, but their influence on attainment was not fully explained. Results therefore highlight the need for better understanding of social and biological mechanisms by which BMI and ADHD negatively influence attainment.

## Methods

### One and two-sample Mendelian randomization

Since SNPs are assigned at conception, associations with SNPs cannot be due to reverse causation or classical confounding^41^. Multiple SNPs associated with a health condition can be combined into a PGS representing genetic liability for a condition. Relative to single SNPs, this improves statistical power. In one-sample Mendelian randomization, causal influence of an exposure is estimated using the PGS as an instrumental variable for the exposure in a two-stage least-squares model. Two-sample Mendelian randomization requires only summary-level results from genome-wide association studies (GWAS)^66^. This compares associations of individual SNPs with an exposure and with an outcome (here, educational attainment). If the exposure causally influences the outcome, the same SNPs should associate with both.

### Study participants

Data came from the Avon Longitudinal Study of Parents and Children (ALSPAC), a birth cohort of children born in south-west England between April 1991 and December 1992 (Supplementary Fig. 1). The total ALSPAC sample comprised 15,454 pregnancies, with 14,901 children alive at 12 months. Data was collected from pregnancy onwards^49,67^. After excluding related individuals, 7856 ALSPAC participants had genetic data, of whom 6113 had GCSE records. These participants comprised the analytic sample. For participants with genetic and GCSE data but missing data on school absence, imputed values of school absence were used. Observed characteristics differed between participants with and without full data (Supplementary Table 1), and multiple imputation with chained equations (*m* = 50) was used to impute remaining missing data (Supplementary Table 2). Power calculations assuming an *R*^2^ for instrument strength of 0.06 (taken from the BMI GWAS^68^) showed that in our sample there was 80% power to detect a causal effect on GCSEs of 0.15 SD with an SD difference in BMI^69^.

Ethical approval for the study was obtained from the ALSPAC Ethics and Law Committee and the Local Research Ethics Committees. Consent for biological samples has been collected in accordance with the Human Tissue Act (2004). Informed consent for the use of data collected via questionnaires and clinics was obtained from participants following the recommendations of the ALSPAC Ethics and Law Committee at the time. Completion of individual questionnaires was taken as consent for use of data from that questionnaire, with additional written consent from parents for use of clinic data. At age 16, young people and their parents gave written informed consent for use of the young person’s genetic information. At age 18, study children were sent ‘fair processing’ materials describing ALSPAC’s intended use of their health and administrative records and were given clear means to consent or object via a written form. Education data were not extracted for participants who objected, or who were not sent fair processing materials^70,71^.

### Measures

ADHD symptoms were based on the Strengths and Difficulties Questionnaire hyperactivity subscale (SDQ-HI), completed by the child’s main carer (usually the child’s mother) in questionnaires administered when children were aged 9 and 13. The SDQ is a validated screening tool for psychiatric disorders at these ages^72^. Depressive symptoms were measured using a validated screening tool, the short-form Mood and Feelings Questionnaire (MFQ)^73^, completed by children at 10 and 13. For autism, a continuous measure of autistic social traits was derived from the Social Communication Disorder Checklist (SCDC)^74^, a validated measure completed by the child’s main carer when the children were aged 10 and 13. BMI (in kg/m^2^) was obtained from height and weight measurements at 10 and 13 and standardized to the 1990 UK Growth Reference by gender and age with STATA’s zanthro package^75^. Resulting *z*-scores, representing SD difference from reference means, were used as continuous variables. Asthma in the past 12 months (yes/no) was defined using mother’s reports of diagnoses, medication use and wheezing symptoms, at 10 and 13. At age 10, but not later, mothers were asked if their children had experienced migraine (yes/no). The study website contains details of available data through a searchable data dictionary and variable search tool: http://www.bristol.ac.uk/alspac/researchers/our-data/.

We consider educational attainment at the end of year 11 (equivalent to 10th grade), when most participants were aged 16, and the end of compulsory education in the UK at the time. Attainment and school absence came from linkage to the National Pupil Database (NPD). Attainment was based on General Certificate of Secondary Education (GCSE) qualifications, compulsory qualifications usually taken at age 16. We used the total GSCE and equivalents points score, a continuous measure (range 0–540), based on a pupil’s best eight subjects, where a one-grade difference in one GCSE subject equates to 6 points. A small number of scores above 464 (8A* grades) reflect pupils who took AS levels early. More information is available from the Department of Education^76^. Absence data was available for academic years 2006–7, 2007–8, and 2008–9, corresponding to school years 9, 10 and 11 for different ALSPAC participants, whose birth dates span almost 2 years. Absence data was available for all sub-cohorts for year 11, most for year 10, but only a small minority for year 9. We therefore considered school absence during years 10 and 11, by imputing each separately and calculating an average post-imputation. (GCSEs are awarded at the end of year 11, so always following the period over which absence was considered.) Absences were analysed as the number of half-day sessions recorded as missed, divided by the number of sessions on which data was available. For most participants, data was available each year for between 280 and 320 sessions, not the 390 of a standard school year, as records cover early September until the end of May. A small minority had data corresponding to fewer sessions (2.3% in year 10, 4.8% in year 11).

ALSPAC children were genotyped using the Illumina HumanHap550 platform, and standard quality control procedures applied. Individuals were excluded for gender mismatches, minimal or excessive heterozygosity, disproportionate individual missingness (>3%) and insufficient sample replication (IBD < 0.8). During genetic quality controls individuals with non-European ancestry were removed, which is standard practice in genetic studies to minimize bias due to ancestral population stratification^77^. SNPs with a minor allele frequency of <1%, call rate of <95% or evidence of Hardy–Weinberg disequilibrium (*p*-value < 5 × 10^−7^) were removed. Cryptic relatedness was measured as proportion of identity by descent (IBD > 0.1). Imputation was performed using Impute v2.2.2 to the 1000Genomes reference panel, and SNPs with poor imputation quality (infoscore < 0.08) removed.

GWAS were used to identify SNPs associated with ADHD, ASD, depression, asthma, migraine, and BMI. We obtained SNP associations for ADHD^78^, depression^48^, ASD^47^, asthma^79^ and migraine^80^ from GWAS including child and adult-onset conditions, since GWAS specifically of child-onset conditions were unavailable. For BMI, we used the largest GWAS of adult BMI. A GWAS of BMI in children exists^81^, but ALSPAC comprised a substantial component of the discovery sample, and such sample overlap can cause bias^82^. When choosing SNPs to include in each PGS, the conventional threshold of genome-wide significance of *p* < 5 × 10^−8^ was applied, except for ASD. As too few SNPs meet that threshold to permit meaningful analysis, a more liberal threshold of *p* < 5 × 10^−7^ was used. Among SNPs available in ALSPAC which had passed standard quality control, we removed non-independent SNPs (linkage disequilibrium clumping threshold *r*^2^ = 0.01, distance = 10,000 kb). Each PGS was calculated in PLINK 1.9 by summing trait-increasing alleles. These were weighted by each allele’s regression coefficient from the relevant GWAS, so that genetic variants with greater effects contributed more to the scores, and standardized (details of GWAS and SNPs are provided in Supplementary Tables 3 and 4).

### Statistical analysis

Analyses in ALSPAC were conducted using Stata v15. The proportion of school sessions missed was considerably skewed, so for analysis was log-transformed after adding 0.01. Coefficients for absence therefore represent percentage change. Linear regression was used to examine associations of health conditions with the two outcomes, attainment and logged school absence. All analyses adjusted for gender and family socioeconomic confounders at birth: maternal age (in years) and parity (categorized as 0, 1, 2, or 3+), maternal educational qualifications (none, CSE, vocational qualifications, O-level, A-level, or university degree), maternal smoking during pregnancy (yes/no), and maternal housing tenure (owner-occupier/council rented/private or housing association rented/other). Sensitivity analyses stratified by school type: mainstream state schools, independent (fee-paying) and other schools (community special schools, pupil referral units, further education colleges). Mediation analysis using STATA’s paramed package considered associations of health with attainment via school absence (the indirect effect) and unexplained by school absence (the direct effect). Models were run separately within imputed datasets and estimates combined across imputations. All analyses clustered standard errors by school. All hypothesis tests were two-sided.

Linear regression was used to examine associations of each PGS with GCSEs and school absence. Genetic models adjusted for gender and 20 principal ancestry components^42^. Where there was evidence of an association and the PGS was a sufficiently strong instrument (first-stage *F*-statistics > 10), PGSs were used as instruments for health conditions at age 10 and 13. A concern in MR studies is pleiotropy, which can bias exposure-outcome causal estimates. This is when alleles related to the exposure influence the outcome via other pathways. Validity of instruments was checked using Stata’s MRRobust package. This applies two-sample MR methodology to the SNPs in each PGS, producing MR-median, MR-modal and MR-Egger estimates^83^.

Using the TwoSampleMR package in R^84^, summary-level MR analyses were performed to assess causal influence of asthma, migraine and BMI on educational attainment in independent adult samples. SNPs associated with educational attainment came from the most recent GWAS of years of schooling in European-ancestry individuals^63^, except for BMI where an earlier GWAS^85^ was used to avoid bias due to sample overlap. Details of GWAS used are given in Supplementary Tables 2 and 3.

## Supplementary information

Supplementary material

## Data Availability

The informed consent obtained from ALSPAC participants does not allow the data to be made freely available through any third party maintained public repository. However, data used for this submission can be made available on request to the ALSPAC Executive. The ALSPAC data management plan describes in detail the policy regarding data sharing, which is through a system of managed open access. Full instructions for applying for data access can be found here: http://www.bristol.ac.uk/alspac/researchers/access/. The GWAS Summary Statistics on which this analysis drew are available from the EBI GWAS Catalog, https://www.ebi.ac.uk/gwas/. Accession numbers are: GCST005839 (depression), GCST007543 (ADHD), GCST007556 (ASD), GCST000804 (asthma), GCST003720 (migraine), GCST006900 (BMI), GCST006442 and GCST003676 (educational attainment).

## Code availability

Stata code used in this analysis is available at https://github.com/ammhughes/childhood-health-and-educational-outcomes.
