# Supplementary material for: Common health conditions in childhood and adolescence, school absence, and educational attainment: Mendelian randomization study
Source: NPJ Sci Learn. 2021 Jan 4;6:1. doi: 10.1038/s41539-020-00080-6 (PMC7782810; doi:10.1038/s41539-020-00080-6)
Supplement: Supplementary file 1 — Supplementary material [file 41539_2020_80_MOESM1_ESM.docx]

**Supplementary material**

**Contents:**

**Supplementary Figure 1: Flow chart of inclusion and exclusion of ALSPAC participants into the study sample**

**Supplementary Table 1: Comparison of observed data between participants with complete and incomplete information**

**Supplementary Table 2: Missingness of variables in unimputed data**

**Supplementary Table 3: GWAS used**

**Supplementary Table 4: SNPs used in polygenic scores**

**Supplementary Table 5: Descriptive Characteristics of Analytic Sample: unimputed data**

**Supplementary Table 6: Association of GCSEs and absenteeism at key stage 4 (age 14-16) with covariates**

**Supplementary Table 7: School type checks: health at 10 and 13 to GCSE points score and absenteeism at age 14-16)**

**Supplementary Table 8: School type checks: associations of health conditions with educational attainment - mediation by school absence at age 14-16**

**Supplementary Table 9: Prediction of phenotypes by polygenic scores in ALSPAC**

**Supplementary Table 10: School type checks: association of polygenic scores with GCSE points score and absenteeism at age 14-16**

**Supplementary Table 11: Robustness checks for individual-participant analyses in ALSPAC using Two-Sample methodology**

**Supplementary Table 12: SNP-specific ratio estimates for the effect of BMI on school absence**

**Supplementary Table 13: Genetic models: association of BMI with educational attainment at 16 and school absence at 14-16**

**Supplementary Table 14: Results of Two-Sample MR with 24 outlier SNPs excluded**

**Supplementary Table 15: Associations of outlier BMI SNPs from TwoSample MR with other traits**

**Supplementary Figure 1:** **Flow chart of inclusion and exclusion of ALSPAC participants into the study sample**

Enrolled in ALSPAC, alive at 1yr, consent not withdrawn (N=14,831)

**Analytic sample (N=6113)**

No school ID, needed for clustering (N=11)

GCSE records available (N=6124)

GCSE records from the National Pupil Database not available (N=1,727)

Related (N=940)

Genetic data not available, or failed genetic quality control (N=6,071)

Unrelated (N=7,851)

Usable genetic data available (N=8,791)

| **Supplementary Table 1: Comparison of observed characteristics between participants with complete and incomplete data (participants alive at 1yr, consent not withdrawn, N=14831^1^)** | | | | |
| --- | --- | --- | --- | --- |
|  |  | **Complete data (N=1,856**) | **Incomplete data (N=12975)** | **p for difference** |
| **Continuous variables** |  | **Mean** | **Mean** | **p (t-test)** |
| Maternal age |  | 27.58 | 28.94 | <0.001 |
| SDQ hyperactivity score^2^ at 10 (114 months) |  | 3.00 | 2.65 | <0.001 |
| SDQ hyperactivity score^2^ at 13 (156 months) |  | 2.97 | 2.73 | <0.001 |
| MFQ score^3^ at 10 (127 months) |  | 4.13 | 3.82 | 0.001 |
| MFQ score^3^ at 13 (154 months) |  | 4.05 | 3.77 | 0.009 |
| SCDC score^4^ at 10 (120 months) |  | 2.47 | 2.03 | <0.001 |
| SCDC score^4^ at 13 (156 months) |  | 2.59 | 2.30 | 0.003 |
| BMI^5^ z-score at 10 (127 months) |  | 0.34 | 0.26 | 0.011 |
| BMI^5^ z-score at 13 (154 months) |  | 0.39 | 0.31 | 0.012 |
| GCSE capped points score^6^ |  | 310.49 | 359.99 | <0.001 |
| Percent of sessions absent, key stage 4 (14-16)^7^ |  | 8.76 | 5.26 | <0.001 |
| Asthma polygenic score^8^ |  | 0.00 | -0.02 | 0.588 |
| Migraine polygenic score^8^ |  | 0.01 | -0.02 | 0.229 |
| BMI polygenic score^8^ |  | -0.02 | 0.00 | 0.548 |
| Autism spectrum disorder polygenic score^8^ |  | 0.01 | -0.03 | 0.128 |
| Attention-deficit hyperactivity disorder polygenic score^8^ |  | 0.00 | -0.04 | 0.103 |
| Depression polygenic score^8^ |  | 0.01 | -0.02 | 0.220 |
| **Categorical variables** | **Category** | **%** | **%** | **p (chi^2^)** |
| gender | male | 51.50 | 48.33 |  |
|  | female | 48.50 | 51.67 | 0.011 |
| maternal educational qualifications | CSE or less | 22.00 | 9.86 |  |
|  | vocational | 10.19 | 8.03 |  |
|  | O level | 33.88 | 38.69 |  |
|  | A level | 21.72 | 26.72 |  |
|  | Degree | 12.22 | 16.70 | <0.001 |
| maternal housing tenure in pregnancy | mortgage/owned outright | 70.88 | 86.58 |  |
|  | council rented | 16.15 | 5.39 |  |
|  | private/other rented | 9.34 | 5.87 |  |
|  | other | 3.63 | 2.16 | <0.001 |
| maternal parity at child’s birth | 0 | 44.05 | 48.17 |  |
|  | 1 | 34.99 | 35.83 |  |
|  | 2 | 14.61 | 12.45 |  |
|  | 3+ | 6.35 | 3.56 | <0.001 |
| mother smoked during pregnancy | no | 70.79 | 84.00 |  |
|  | yes | 29.21 | 16.00 | <0.001 |
| migraines at 10 | no | 94.70 | 96.34 |  |
|  | yes | 5.30 | 3.66 | 0.005 |
| asthma at 10 (128 months) | no | 88.08 | 87.77 |  |
|  | yes | 11.92 | 12.23 | 0.723 |
| asthma at 13 (157 months) | no | 88.03 | 88.47 |  |
|  | yes | 11.97 | 11.53 | 0.613 |
| school type^9^ at key stage 4 (age 14-16) | mainstream state | 91.37 | 100.00 |  |
|  | independent | 6.03 | 0.00 |  |
|  | other | 2.60 | 0.00 | <0.001 |
| ^1^This table compares all 14813 participants who were alive at 1yr and for whom gender was known, as these participants formed the imputation sample. The analytic sample comprised the subset of these individuals who had nonmissing genetic data and linked GCSE records, and were unrelated. ^2^SDQ=Strengths and Difficulties Questionnaire, for ADHD symptoms.  ^3^MFQ=Mood and Feelings Questionnaire, for depressive symptoms. ^4^SCDC=Social Communication Disorder Checklist, for autistic social traits. ^5^Using 1990 UK Growth Reference. Values represent standard deviation difference from age- and gender-specific reference mean. ^6^A small number of scores above 464 (8 A* grades) reflect pupils who took AS levels early. ^7^Absences were analysed as the number of half-day sessions recorded as missed, divided by the number of sessions on which data was available. For most participants, data was available each year for between 280 and 320 sessions, not the 390 of a standard school year, as records cover early September until the end of May. A small minority (2.3% in year 10, 4.8% in year 11) had data corresponding to fewer sessions. ^8^Polygenic scores are standardized. ^9^Mainstream state schools: community, voluntary controlled or aided, foundation, city technology college, academy. Other schools: community special, pupil referral unit, further education college | | | | |

| **Supplementary Table 2: Missingness of variables in unimputed data** | | | | | | | | | |
| --- | --- | --- | --- | --- | --- | --- | --- | --- | --- |
| **Imputed variables** | |  |  | **Imputation sample^1^ (N=14,831)** | | | **Analytic sample (N=6133)** | | |
|  |  |  |  | Observed | Imputed | % imputed | Observed | Imputed | % imputed |
| SDQ hyperactivity score^2^ at 10 | | |  | 7,599 | 7,232 | 0.49 | 4,248 | 1,885 | 0.31 |
| SDQ hyperactivity score^2^ at 13 | | |  | 6,901 | 7,930 | 0.53 | 3,959 | 2,174 | 0.35 |
| MFQ score^3^ at 10 | |  |  | 7,409 | 7,422 | 0.50 | 4,432 | 1,701 | 0.28 |
| MFQ score^3^ at 13 | |  |  | 6,778 | 8,053 | 0.54 | 4,160 | 1,973 | 0.32 |
| SCDC score^4^ at 10 | |  |  | 7,840 | 6,991 | 0.47 | 4,372 | 1,761 | 0.29 |
| SCDC score^4^ at 13 | |  |  | 7,098 | 7,733 | 0.52 | 4,093 | 2,040 | 0.33 |
| BMI^5^ z-score at 10 | |  |  | 7,461 | 7,370 | 0.50 | 4,468 | 1,665 | 0.27 |
| BMI^5^ z-score at 13 | |  |  | 6,699 | 8,132 | 0.55 | 4,116 | 2,017 | 0.33 |
| migraines at 10 | |  |  | 7,238 | 7,593 | 0.51 | 4,354 | 1,779 | 0.29 |
| asthma at 128 months | | |  | 7,777 | 7,054 | 0.48 | 4,342 | 1,791 | 0.29 |
| asthma at 157 months | | |  | 7,018 | 7,813 | 0.53 | 4,013 | 2,120 | 0.35 |
| GCSE capped points score | | |  | 10,874 | 3,957 | 0.27 | 6,113 | 0 | 0.00 |
| Total absence at year 11 (age 15-16) | | | | 10,214 | 4,617 | 0.31 | 5,702 | 431 | 0.07 |
| Total absence at year 10 (age 14-15) | | | | 8,109 | 6,722 | 0.45 | 4,520 | 1,613 | 0.26 |
| Total absence at year 10-11 (age 14-16) | | | | 7,992 | 6,839 | 0.46 | 4,472 | 1,661 | 0.27 |
| Gender |  |  |  | 14,831 | 0 | 0.00 | 6,113 | 0 | 0.00 |
| Maternal age | |  |  | 13,176 | 1,655 | 0.11 | 5,689 | 444 | 0.07 |
| maternal educational qualifications | | | | 12,391 | 2,440 | 0.16 | 5,527 | 606 | 0.10 |
| maternal housing tenure in pregnancy | | | | 13,788 | 1,043 | 0.07 | 5,964 | 169 | 0.03 |
| maternal parity at child’s birth | | |  | 12,904 | 1,927 | 0.13 | 5,597 | 536 | 0.09 |
| mother smoked during pregnancy | | | | 13,244 | 1,587 | 0.11 | 5,698 | 435 | 0.07 |
| school type at key stage 4 (age 14-16) | | | | 10,937 | 3,894 | 0.26 | 6,113 | 0 | 0.00 |
| Polygenic score for ADHD | | |  | 8,791 | 6,040 | 0.41 | 6,113 | 0 | 0.00 |
| Polygenic score for ASD | | |  | 8,791 | 6,040 | 0.41 | 6,113 | 0 | 0.00 |
| Polygenic score for depression | | |  | 8,791 | 6,040 | 0.41 | 6,113 | 0 | 0.00 |
| Polygenic score for asthma | | |  | 8,791 | 6,040 | 0.41 | 6,113 | 0 | 0.00 |
| Polygenic score for BMI | | |  | 8,791 | 6,040 | 0.41 | 6,113 | 0 | 0.00 |
| Polygenic score for migraine | | |  | 8,791 | 6,040 | 0.41 | 6,113 | 0 | 0.00 |
| ^1^Imputation by chained equations was performed for ALSPAC participants alive at 1 year and for whom sex was known. Analysis was restricted to those with genetic data and GCSE records. ^2^SDQ=Strengths and Difficulties Questionnaire, for ADHD symptoms. ^3^MFQ=Mood and Feelings Questionnaire, for depressive symptoms. ^4^SCDC=Social Communication Disorder Checklist, for autistic social traits. ^5^Using 1990 UK Growth Reference. | | | | | | | | | |

| **Supplementary Table 3: GWAS used** | | | | | | |
| --- | --- | --- | --- | --- | --- | --- |
| *Health Measure* | *Author* | *Year* | *Consortium* | *GWAS Source* | *Number of SNPs used in PGS* | *Number of SNPs used in 2-sample MR* |
| Attention-Deficit Hyperactivity Disorder^1^ | Demontis | 2018 | Psychiatric Genetics Consortium | PGC website | 9 | 8 |
| Autism Spectrum Disorder | Grove | 2019 | Psychiatric Genetics Consortium | PGC website | 10 | 7 |
| Depression | Wray | 2018 | Psychiatric Genetics Consortium | MR Base: id 1187/1188^2^ | 41 | 4^3^ |
| Asthma | Moffatt | 2010 | GABRIEL^4^ | MR Base: id 44 | 6 | 8 |
| Migraine | Gormley | 2016 | International Headache Genetics Consortium | EBI GWAS Database: id GCST003720 | 32 | 29 |
| Body Mass Index | Yengo | 2018 | GIANT+UKBiobank | Publication supplement | 965 | 945 |
| ^1^Coefficients as calculated in Europeans only, available from PGC website. ^2^Version including 23andme used for polygenic scores, version excluding 23andme used for 2-sample MR to avoid sample overlap. ^3^Only 5 SNPs met the threshold of 5x10^-8^ when 23andme is excluded from the depression GWAS. ^4^GABRIEL: A Multidisciplinary Study to Identify the Genetic and Environmental Causes of Asthma in the European Community. | | | | | | |

| **Supplementary Table 4: SNPs used in polygenic scores** | | | | | | | | | |
| --- | --- | --- | --- | --- | --- | --- | --- | --- | --- |
| **Trait** | **GWAS author** | **GWAS Source** | **SNP** | **Effect Allele** | **Other Allele** | **EAF** | **Beta (log odds)** | **p** | **MR-Base/EBI Study ID** |
| ADHD^1^ | Demontis | PGC website | rs17531412 | A | G | NA | 0.11 | 1.07E-12 | NA |
| ADHD | Demontis | PGC website | rs4858241 | T | G | NA | 0.08 | 8.17E-09 | NA |
| ADHD | Demontis | PGC website | rs4916723 | A | C | NA | -0.08 | 1.81E-08 | NA |
| ADHD | Demontis | PGC website | rs10262192 | A | G | NA | 0.07 | 3.66E-08 | NA |
| ADHD | Demontis | PGC website | rs74760947 | A | G | NA | -0.18 | 1.39E-08 | NA |
| ADHD | Demontis | PGC website | rs11591402 | A | T | NA | -0.09 | 1.76E-08 | NA |
| ADHD | Demontis | PGC website | rs1427829 | A | G | NA | 0.08 | 1.35E-09 | NA |
| ADHD | Demontis | PGC website | rs8039398 | T | C | NA | -0.08 | 2.99E-09 | NA |
| ADHD | Demontis | PGC website | rs212178 | A | G | NA | -0.12 | 1.20E-08 | NA |
| **Trait** | **GWAS author** | **GWAS Source** | **SNP** | **Effect Allele** | **Other Allele** | **EAF** | **Beta (log odds)** | **p** | **MR-Base/EBI Study ID** |
| ASD | Grove | MR Base | rs112635299 | T | G | NA | 0.22 | 3.04E-07 | 1185 |
| ASD | Grove | MR Base | rs325485 | A | G | NA | 0.07 | 3.25E-07 | 1185 |
| ASD | Grove | MR Base | rs6701243 | A | C | NA | 0.07 | 3.07E-07 | 1185 |
| ASD | Grove | MR Base | rs1452075 | T | C | NA | 0.08 | 2.07E-07 | 1185 |
| ASD | Grove | MR Base | rs45595836 | T | C | NA | 0.14 | 3.13E-07 | 1185 |
| ASD | Grove | MR Base | rs111931861 | A | G | NA | -0.22 | 1.12E-07 | 1185 |
| ASD | Grove | MR Base | rs10099100 | C | G | NA | 0.08 | 1.07E-08 | 1185 |
| ASD | Grove | MR Base | rs910805 | A | G | NA | -0.10 | 2.04E-09 | 1185 |
| ASD | Grove | MR Base | rs139229207 | C | CA | NA | -0.07 | 2.59E-07 | 1185 |
| ASD | Grove | MR Base | rs2391769 | A | G | NA | -0.08 | 1.14E-07 | 1185 |
| **Trait** | **GWAS author** | **GWAS Source** | **SNP** | **Effect Allele** | **Other Allele** | **EAF** | **Beta (log odds)** | **p** | **MR-Base/EBI Study ID** |
| Depression | Wray^2^ | MR Base | rs4904738 | T | C | NA | -0.03 | 2.57E-09 | 1187 |
| Depression | Wray | MR Base | rs915057 | A | G | NA | -0.03 | 7.61E-10 | 1187 |
| Depression | Wray | MR Base | rs4261101 | A | G | NA | -0.03 | 1.04E-08 | 1187 |
| Depression | Wray | MR Base | rs9427672 | A | G | NA | -0.03 | 3.12E-08 | 1187 |
| **Trait** | **GWAS author** | **GWAS Source** | **SNP** | **Effect Allele** | **Other Allele** | **EAF** | **Beta (log odds)** | **p** | **MR-Base/EBI Study ID** |
| Depression | Wray | MR Base | rs116755193 | T | C | NA | -0.03 | 7.01E-09 | 1187 |
| Depression | Wray | MR Base | rs11135349 | A | C | NA | -0.03 | 1.09E-09 | 1187 |
| Depression | Wray | MR Base | rs12666117 | A | G | NA | 0.03 | 1.35E-08 | 1187 |
| Depression | Wray | MR Base | rs4074723 | A | C | NA | -0.03 | 3.12E-08 | 1187 |
| Depression | Wray | MR Base | rs7200826 | T | C | NA | 0.03 | 2.43E-08 | 1187 |
| Depression | Wray | MR Base | rs12958048 | A | G | NA | 0.03 | 3.61E-11 | 1187 |
| Depression | Wray | MR Base | rs1354115 | A | C | NA | 0.03 | 2.37E-08 | 1187 |
| Depression | Wray | MR Base | rs12129573 | A | C | NA | 0.03 | 4.01E-12 | 1187 |
| Depression | Wray | MR Base | rs2389016 | T | C | NA | 0.03 | 1.02E-08 | 1187 |
| Depression | Wray | MR Base | rs7430565 | A | G | NA | -0.03 | 2.87E-09 | 1187 |
| Depression | Wray | MR Base | rs34215985 | C | G | NA | -0.04 | 3.13E-09 | 1187 |
| Depression | Wray | MR Base | rs2005864 | T | C | NA | 0.03 | 6.73E-09 | 1187 |
| Depression | Wray | MR Base | rs1806153 | T | G | NA | 0.04 | 1.18E-09 | 1187 |
| Depression | Wray | MR Base | rs8063603 | A | G | NA | -0.03 | 6.87E-09 | 1187 |
| Depression | Wray | MR Base | rs7856424 | T | C | NA | -0.03 | 8.48E-09 | 1187 |
| Depression | Wray | MR Base | rs12552 | A | G | NA | 0.04 | 6.07E-19 | 1187 |
| Depression | Wray | MR Base | rs10149470 | A | G | NA | -0.03 | 3.05E-09 | 1187 |
| Depression | Wray | MR Base | rs62099069 | A | T | NA | -0.03 | 1.31E-08 | 1187 |
| Depression | Wray | MR Base | rs10959913 | T | G | NA | 0.03 | 5.06E-09 | 1187 |
| Depression | Wray | MR Base | rs61867293 | T | C | NA | -0.04 | 6.97E-10 | 1187 |
| Depression | Wray | MR Base | rs159963 | A | C | NA | -0.03 | 3.19E-08 | 1187 |
| Depression | Wray | MR Base | rs1432639 | A | C | NA | 0.04 | 4.55E-15 | 1187 |
| Depression | Wray | MR Base | rs1226412 | T | C | NA | 0.03 | 2.38E-08 | 1187 |
| Depression | Wray | MR Base | rs247910 | A | G | NA | -0.03 | 1.07E-10 | 1187 |
| Depression | Wray | MR Base | rs1363104 | C | G | NA | 0.03 | 7.38E-11 | 1187 |
| Depression | Wray | MR Base | rs11643192 | A | C | NA | 0.03 | 3.36E-08 | 1187 |
| Depression | Wray | MR Base | rs7198928 | T | C | NA | 0.03 | 1.00E-08 | 1187 |
| Depression | Wray | MR Base | rs5758265 | A | G | NA | 0.03 | 7.55E-09 | 1187 |
| Depression | Wray | MR Base | rs8025231 | A | C | NA | -0.03 | 2.36E-12 | 1187 |
| **Trait** | **GWAS author** | **GWAS Source** | **SNP** | **Effect Allele** | **Other Allele** | **EAF** | **Beta (log odds)** | **p** | **MR-Base/EBI Study ID** |
| Depression | Wray | MR Base | rs11663393 | A | G | NA | 0.03 | 1.65E-08 | 1187 |
| Depression | Wray | MR Base | rs4869056 | A | G | NA | -0.03 | 6.80E-09 | 1187 |
| Depression | Wray | MR Base | rs6905391 | A | G | NA | -0.04 | 1.35E-10 | 1187 |
| Depression | Wray | MR Base | rs9402472 | A | G | NA | 0.03 | 2.78E-08 | 1187 |
| Depression | Wray | MR Base | rs10950398 | A | G | NA | 0.03 | 2.55E-08 | 1187 |
| Depression | Wray | MR Base | rs17727765 | T | C | NA | -0.05 | 8.51E-09 | 1187 |
| Depression | Wray | MR Base | rs4143229 | A | C | NA | -0.05 | 2.51E-08 | 1187 |
| **Trait** | **GWAS author** | **Source** | **SNP** | **Effect Allele** | **Other Allele** | **EAF** | **Beta (log odds)** | **p** | **MR-Base/EBI Study ID** |
| Asthma | Moffatt | MR Base | rs10206753 | T | C | 0.61 | 0.14 | 5.55E-12 | 44 |
| Asthma | Moffatt | MR Base | rs992969 | G | A | 0.79 | -0.17 | 4.03E-14 | 44 |
| Asthma | Moffatt | MR Base | rs6871536 | T | C | 0.77 | -0.13 | 1.89E-08 | 44 |
| Asthma | Moffatt | MR Base | rs1837253 | T | C | 0.26 | -0.15 | 3.03E-10 | 44 |
| Asthma | Moffatt | MR Base | rs17843604 | T | C | 0.54 | 0.15 | 1.68E-10 | 44 |
| Asthma | Moffatt | MR Base | rs8067378 | G | A | 0.49 | -0.17 | 1.04E-17 | 44 |
| **Trait** | **Author** | **Source** | **SNP** | **Effect Allele** | **Other Allele** | **EAF** | **Beta (log odds)** | **p** | **MR-Base/EBI Study ID** |
| Migraine | Gormley | EBI GWAS catalogue | rs10155855 | T | A | 0.05 | 0.08 | 2.00E-08 | GCST003720 |
| Migraine | Gormley | EBI GWAS catalogue | rs10218452 | G | A | 0.22 | 0.10 | 5.00E-38 | GCST003720 |
| Migraine | Gormley | EBI GWAS catalogue | rs1024905 | G | C | 0.47 | 0.06 | 2.00E-17 | GCST003720 |
| Migraine | Gormley | EBI GWAS catalogue | rs10456100 | T | C | 0.28 | 0.06 | 7.00E-13 | GCST003720 |
| Migraine | Gormley | EBI GWAS catalogue | rs10895275 | A | T | 0.33 | 0.04 | 2.00E-08 | GCST003720 |
| Migraine | Gormley | EBI GWAS catalogue | rs11031122 | C | T | 0.24 | 0.04 | 4.00E-08 | GCST003720 |
| Migraine | Gormley | EBI GWAS catalogue | rs11172113 | T | C | 0.58 | 0.11 | 6.00E-49 | GCST003720 |
| Migraine | Gormley | EBI GWAS catalogue | rs11624776 | A | C | 0.69 | 0.04 | 8.00E-09 | GCST003720 |
| Migraine | Gormley | EBI GWAS catalogue | rs12260159 | G | A | 0.93 | 0.08 | 3.00E-10 | GCST003720 |
| Migraine | Gormley | EBI GWAS catalogue | rs1268083 | T | C | 0.52 | 0.04 | 5.00E-09 | GCST003720 |
| Migraine | Gormley | EBI GWAS catalogue | rs13078967 | A | C | 0.97 | 0.14 | 2.00E-09 | GCST003720 |
| Migraine | Gormley | EBI GWAS catalogue | rs138556413 | C | T | 0.97 | 0.13 | 2.00E-08 | GCST003720 |
| **Trait** | **GWAS author** | **GWAS Source** | **SNP** | **Effect Allele** | **Other Allele** | **EAF** | **Beta (log odds)** | **p** | **MR-Base/EBI Study ID** |
| Migraine | Gormley | EBI GWAS catalogue | rs1572668 | G | A | 0.48 | 0.04 | 2.00E-08 | GCST003720 |
| Migraine | Gormley | EBI GWAS catalogue | rs186166891 | T | A | 0.11 | 0.09 | 1.00E-15 | GCST003720 |
| Migraine | Gormley | EBI GWAS catalogue | rs1925950 | G | A | 0.35 | 0.07 | 9.00E-22 | GCST003720 |
| Migraine | Gormley | EBI GWAS catalogue | rs2078371 | C | T | 0.12 | 0.10 | 4.00E-24 | GCST003720 |
| Migraine | Gormley | EBI GWAS catalogue | rs2223089 | G | C | 0.92 | 0.07 | 3.00E-08 | GCST003720 |
| Migraine | Gormley | EBI GWAS catalogue | rs2506142 | G | A | 0.17 | 0.06 | 2.00E-09 | GCST003720 |
| Migraine | Gormley | EBI GWAS catalogue | rs28455731 | T | G | 0.16 | 0.06 | 7.00E-09 | GCST003720 |
| Migraine | Gormley | EBI GWAS catalogue | rs4081947 | G | A | 0.34 | 0.03 | 3.00E-09 | GCST003720 |
| Migraine | Gormley | EBI GWAS catalogue | rs4814864 | C | NA | 0.26 | 0.07 | 2.00E-19 | GCST003720 |
| Migraine | Gormley | EBI GWAS catalogue | rs4839827 | C | T | 0.47 | 0.05 | 6.00E-10 | GCST003720 |
| Migraine | Gormley | EBI GWAS catalogue | rs4910165 | G | C | 0.67 | 0.06 | 3.00E-11 | GCST003720 |
| Migraine | Gormley | EBI GWAS catalogue | rs561561 | A | T | 0.88 | 0.06 | 3.00E-08 | GCST003720 |
| Migraine | Gormley | EBI GWAS catalogue | rs566529 | G | T | 0.85 | 0.06 | 3.00E-09 | GCST003720 |
| Migraine | Gormley | EBI GWAS catalogue | rs6478241 | A | G | 0.36 | 0.05 | 1.00E-12 | GCST003720 |
| Migraine | Gormley | EBI GWAS catalogue | rs6693567 | C | T | 0.27 | 0.05 | 1.00E-08 | GCST003720 |
| Migraine | Gormley | EBI GWAS catalogue | rs6791480 | T | C | 0.31 | 0.04 | 8.00E-09 | GCST003720 |
| Migraine | Gormley | EBI GWAS catalogue | rs75213074 | C | T | 0.97 | 0.12 | 7.00E-09 | GCST003720 |
| Migraine | Gormley | EBI GWAS catalogue | rs75473620 | A | T | 0.96 | 0.11 | 6.00E-09 | GCST003720 |
| Migraine | Gormley | EBI GWAS catalogue | rs9349379 | A | G | 0.59 | 0.07 | 6.00E-22 | GCST003720 |
| **Trait** | **GWAS author** | **Source** | **SNP** | **Effect Allele** | **Other Allele** | **EAF** | **beta** | **p** | **MR-Base/EBI Study ID** |
| BMI | Yengo | Publication supplement | rs10007906 | A | C | 0.3603 | 0.0133 | 0.0018 | NA |
| BMI | Yengo | Publication supplement | rs10009336 | T | C | 0.1638 | -0.014 | 0.0022 | NA |
| BMI | Yengo | Publication supplement | rs1000940 | A | G | 0.7013 | -0.0154 | 0.0018 | NA |
| BMI | Yengo | Publication supplement | rs10058464 | A | C | 0.1119 | -0.0175 | 0.0027 | NA |
| BMI | Yengo | Publication supplement | rs1006353 | A | G | 0.2485 | 0.0126 | 0.0019 | NA |
| BMI | Yengo | Publication supplement | rs1006896 | A | C | 0.8939 | 0.0234 | 0.0027 | NA |
| BMI | Yengo | Publication supplement | rs10071816 | A | G | 0.1941 | 0.0123 | 0.0022 | NA |
| BMI | Yengo | Publication supplement | rs1007934 | A | G | 0.415 | -0.012 | 0.0017 | NA |
| **Trait** | **GWAS author** | **GWAS Source** | **SNP** | **Effect Allele** | **Other Allele** | **EAF** | **Beta (log odds)** | **p** | **MR-Base/EBI Study ID** |
| BMI | Yengo | Publication supplement | rs10118866 | T | G | 0.2238 | 0.012 | 0.0021 | NA |
| BMI | Yengo | Publication supplement | rs10132280 | A | C | 0.3017 | -0.0223 | 0.0018 | NA |
| BMI | Yengo | Publication supplement | rs10135922 | A | C | 0.2699 | -0.0107 | 0.0019 | NA |
| BMI | Yengo | Publication supplement | rs1014194 | A | C | 0.6434 | 0.0102 | 0.0018 | NA |
| BMI | Yengo | Publication supplement | rs10144318 | A | C | 0.489 | 0.0092 | 0.0016 | NA |
| BMI | Yengo | Publication supplement | rs10145461 | T | G | 0.4628 | -0.0142 | 0.0017 | NA |
| BMI | Yengo | Publication supplement | rs10146527 | T | C | 0.6361 | 0.0137 | 0.0017 | NA |
| BMI | Yengo | Publication supplement | rs10163018 | T | C | 0.3748 | -0.0114 | 0.0018 | NA |
| BMI | Yengo | Publication supplement | rs10176705 | T | C | 0.3825 | -0.0122 | 0.0018 | NA |
| BMI | Yengo | Publication supplement | rs10182181 | A | G | 0.5247 | -0.0325 | 0.0016 | NA |
| BMI | Yengo | Publication supplement | rs10192119 | T | G | 0.8327 | -0.0166 | 0.0022 | NA |
| BMI | Yengo | Publication supplement | rs10197031 | T | C | 0.7166 | -0.0166 | 0.0019 | NA |
| BMI | Yengo | Publication supplement | rs10198345 | T | C | 0.6711 | -0.0109 | 0.0018 | NA |
| BMI | Yengo | Publication supplement | rs1020548 | A | G | 0.8344 | -0.0132 | 0.0023 | NA |
| BMI | Yengo | Publication supplement | rs1021066 | T | G | 0.4763 | -0.0096 | 0.0017 | NA |
| BMI | Yengo | Publication supplement | rs10211055 | T | C | 0.6544 | -0.0157 | 0.0018 | NA |
| BMI | Yengo | Publication supplement | rs10243319 | T | C | 0.6061 | 0.0107 | 0.0018 | NA |
| BMI | Yengo | Publication supplement | rs10259786 | A | G | 0.6463 | 0.0114 | 0.0018 | NA |
| BMI | Yengo | Publication supplement | rs10263780 | A | G | 0.139 | -0.0157 | 0.0027 | NA |
| BMI | Yengo | Publication supplement | rs10269783 | A | G | 0.3896 | 0.0133 | 0.0017 | NA |
| BMI | Yengo | Publication supplement | rs1035010 | T | C | 0.2566 | 0.0139 | 0.002 | NA |
| BMI | Yengo | Publication supplement | rs1038088 | T | G | 0.4919 | -0.0117 | 0.0016 | NA |
| BMI | Yengo | Publication supplement | rs10408013 | T | C | 0.2908 | 0.0111 | 0.0018 | NA |
| BMI | Yengo | Publication supplement | rs10433609 | A | T | 0.8316 | 0.0162 | 0.0023 | NA |
| BMI | Yengo | Publication supplement | rs10438964 | T | C | 0.2757 | -0.0127 | 0.0019 | NA |
| BMI | Yengo | Publication supplement | rs1045411 | T | C | 0.2647 | -0.0148 | 0.0019 | NA |
| BMI | Yengo | Publication supplement | rs10460960 | A | G | 0.8876 | 0.0197 | 0.0025 | NA |
| BMI | Yengo | Publication supplement | rs10481754 | T | C | 0.4538 | -0.0099 | 0.0017 | NA |
| BMI | Yengo | Publication supplement | rs10483389 | T | C | 0.0402 | 0.0342 | 0.0042 | NA |
| **Trait** | **GWAS author** | **Source** | **SNP** | **Effect Allele** | **Other Allele** | **EAF** | **beta** | **p** | **MR-Base/EBI Study ID** |
| BMI | Yengo | Publication supplement | rs1048932 | A | C | 0.4162 | -0.016 | 0.0017 | NA |
| BMI | Yengo | Publication supplement | rs10497810 | T | C | 0.1729 | -0.0167 | 0.0022 | NA |
| BMI | Yengo | Publication supplement | rs10499275 | C | G | 0.1653 | 0.0154 | 0.0023 | NA |
| BMI | Yengo | Publication supplement | rs10510419 | T | G | 0.1416 | -0.0177 | 0.0023 | NA |
| BMI | Yengo | Publication supplement | rs10514222 | A | G | 0.906 | 0.0164 | 0.0029 | NA |
| BMI | Yengo | Publication supplement | rs10732321 | C | G | 0.1433 | 0.0145 | 0.0025 | NA |
| BMI | Yengo | Publication supplement | rs10733051 | A | G | 0.5198 | 0.0097 | 0.0016 | NA |
| BMI | Yengo | Publication supplement | rs10745785 | T | C | 0.6674 | -0.0111 | 0.0018 | NA |
| BMI | Yengo | Publication supplement | rs10757826 | A | G | 0.3252 | 0.0105 | 0.0018 | NA |
| BMI | Yengo | Publication supplement | rs1075901 | T | C | 0.4361 | -0.0121 | 0.0016 | NA |
| BMI | Yengo | Publication supplement | rs10765208 | T | G | 0.0691 | 0.0191 | 0.0035 | NA |
| BMI | Yengo | Publication supplement | rs10768994 | T | C | 0.5663 | 0.0114 | 0.0017 | NA |
| BMI | Yengo | Publication supplement | rs10779751 | A | G | 0.2744 | 0.0139 | 0.0018 | NA |
| BMI | Yengo | Publication supplement | rs10795422 | A | G | 0.3095 | -0.0139 | 0.0019 | NA |
| BMI | Yengo | Publication supplement | rs10797115 | T | C | 0.5365 | 0.0124 | 0.0017 | NA |
| BMI | Yengo | Publication supplement | rs10803762 | A | G | 0.6781 | 0.0116 | 0.0018 | NA |
| BMI | Yengo | Publication supplement | rs10811901 | A | G | 0.5591 | 0.0111 | 0.0017 | NA |
| BMI | Yengo | Publication supplement | rs10818810 | A | G | 0.3908 | 0.0126 | 0.0017 | NA |
| BMI | Yengo | Publication supplement | rs10818938 | A | G | 0.4215 | 0.0114 | 0.0017 | NA |
| BMI | Yengo | Publication supplement | rs10824218 | A | T | 0.5522 | 0.0122 | 0.0018 | NA |
| BMI | Yengo | Publication supplement | rs10830452 | A | G | 0.6712 | -0.0111 | 0.0018 | NA |
| BMI | Yengo | Publication supplement | rs10832778 | C | G | 0.3778 | -0.0125 | 0.0017 | NA |
| BMI | Yengo | Publication supplement | rs10838465 | A | C | 0.6967 | 0.014 | 0.0019 | NA |
| BMI | Yengo | Publication supplement | rs10840606 | A | G | 0.8259 | -0.0164 | 0.0024 | NA |
| BMI | Yengo | Publication supplement | rs10842240 | C | G | 0.1158 | 0.0218 | 0.0027 | NA |
| BMI | Yengo | Publication supplement | rs10846428 | T | C | 0.7693 | -0.0106 | 0.0019 | NA |
| BMI | Yengo | Publication supplement | rs10851523 | C | G | 0.2885 | -0.0108 | 0.0019 | NA |
| BMI | Yengo | Publication supplement | rs10878946 | T | C | 0.714 | -0.0141 | 0.0019 | NA |
| BMI | Yengo | Publication supplement | rs10883553 | A | C | 0.4511 | 0.0119 | 0.0018 | NA |
| **Trait** | **GWAS author** | **Source** | **SNP** | **Effect Allele** | **Other Allele** | **EAF** | **beta** | **p** | **MR-Base/EBI Study ID** |
| BMI | Yengo | Publication supplement | rs10883759 | A | G | 0.3023 | -0.0122 | 0.0018 | NA |
| BMI | Yengo | Publication supplement | rs10887578 | C | G | 0.4896 | 0.0128 | 0.0017 | NA |
| BMI | Yengo | Publication supplement | rs10889550 | A | G | 0.9553 | -0.0243 | 0.0041 | NA |
| BMI | Yengo | Publication supplement | rs10904675 | A | C | 0.2342 | -0.0117 | 0.0021 | NA |
| BMI | Yengo | Publication supplement | rs10915840 | A | G | 0.283 | -0.0118 | 0.0019 | NA |
| BMI | Yengo | Publication supplement | rs10920678 | A | G | 0.4291 | 0.0155 | 0.0016 | NA |
| BMI | Yengo | Publication supplement | rs10923724 | T | C | 0.5725 | -0.0118 | 0.0016 | NA |
| BMI | Yengo | Publication supplement | rs10929925 | A | C | 0.4301 | -0.0143 | 0.0016 | NA |
| BMI | Yengo | Publication supplement | rs10938397 | A | G | 0.5683 | -0.0324 | 0.0016 | NA |
| BMI | Yengo | Publication supplement | rs10939792 | C | G | 0.671 | -0.0152 | 0.0019 | NA |
| BMI | Yengo | Publication supplement | rs10942267 | A | G | 0.6912 | 0.0156 | 0.0019 | NA |
| BMI | Yengo | Publication supplement | rs10942476 | A | G | 0.5107 | -0.0099 | 0.0017 | NA |
| BMI | Yengo | Publication supplement | rs10962549 | T | C | 0.168 | 0.0198 | 0.0023 | NA |
| BMI | Yengo | Publication supplement | rs10971721 | T | C | 0.109 | -0.0199 | 0.0028 | NA |
| BMI | Yengo | Publication supplement | rs10975933 | C | G | 0.6556 | 0.0122 | 0.0018 | NA |
| BMI | Yengo | Publication supplement | rs10989568 | A | G | 0.4619 | 0.0107 | 0.0017 | NA |
| BMI | Yengo | Publication supplement | rs11001963 | T | C | 0.5644 | 0.0108 | 0.0018 | NA |
| BMI | Yengo | Publication supplement | rs11056875 | T | C | 0.1469 | 0.0138 | 0.0024 | NA |
| BMI | Yengo | Publication supplement | rs11066188 | A | G | 0.4181 | -0.012 | 0.0017 | NA |
| BMI | Yengo | Publication supplement | rs11074446 | T | C | 0.8694 | 0.0225 | 0.0024 | NA |
| BMI | Yengo | Publication supplement | rs11075489 | T | C | 0.4828 | -0.0111 | 0.0017 | NA |
| BMI | Yengo | Publication supplement | rs11079849 | T | C | 0.3199 | -0.0188 | 0.0019 | NA |
| BMI | Yengo | Publication supplement | rs11084553 | A | G | 0.8482 | 0.021 | 0.0024 | NA |
| BMI | Yengo | Publication supplement | rs11089885 | T | C | 0.5384 | -0.0103 | 0.0018 | NA |
| BMI | Yengo | Publication supplement | rs11096549 | T | C | 0.7112 | 0.0109 | 0.0019 | NA |
| BMI | Yengo | Publication supplement | rs11115176 | T | C | 0.7601 | 0.0121 | 0.0019 | NA |
| BMI | Yengo | Publication supplement | rs11118308 | A | G | 0.5297 | 0.0101 | 0.0016 | NA |
| BMI | Yengo | Publication supplement | rs11119208 | A | G | 0.387 | 0.0095 | 0.0017 | NA |
| BMI | Yengo | Publication supplement | rs11121210 | T | C | 0.3713 | -0.0108 | 0.0018 | NA |
| **Trait** | **GWAS author** | **Source** | **SNP** | **Effect Allele** | **Other Allele** | **EAF** | **beta** | **p** | **MR-Base/EBI Study ID** |
| BMI | Yengo | Publication supplement | rs11128904 | C | G | 0.7273 | 0.0106 | 0.0019 | NA |
| BMI | Yengo | Publication supplement | rs11138082 | T | C | 0.7971 | 0.0123 | 0.0022 | NA |
| BMI | Yengo | Publication supplement | rs11138313 | A | G | 0.9015 | 0.0169 | 0.0028 | NA |
| BMI | Yengo | Publication supplement | rs11150911 | A | C | 0.2809 | 0.0133 | 0.0018 | NA |
| BMI | Yengo | Publication supplement | rs11165643 | T | C | 0.5828 | 0.0206 | 0.0017 | NA |
| BMI | Yengo | Publication supplement | rs11170468 | A | C | 0.7674 | 0.0123 | 0.0019 | NA |
| BMI | Yengo | Publication supplement | rs1117080 | C | G | 0.7015 | 0.011 | 0.0019 | NA |
| BMI | Yengo | Publication supplement | rs11173522 | A | C | 0.2078 | 0.0128 | 0.0021 | NA |
| BMI | Yengo | Publication supplement | rs11191548 | T | C | 0.9181 | -0.0268 | 0.003 | NA |
| BMI | Yengo | Publication supplement | rs11246991 | T | C | 0.1451 | 0.0144 | 0.0026 | NA |
| BMI | Yengo | Publication supplement | rs11251352 | A | G | 0.4012 | -0.0109 | 0.0018 | NA |
| BMI | Yengo | Publication supplement | rs11264483 | C | G | 0.6123 | 0.0137 | 0.0018 | NA |
| BMI | Yengo | Publication supplement | rs1128249 | T | G | 0.4003 | 0.0113 | 0.0017 | NA |
| BMI | Yengo | Publication supplement | rs11496125 | T | C | 0.4212 | 0.0169 | 0.0017 | NA |
| BMI | Yengo | Publication supplement | rs11505821 | A | T | 0.9399 | -0.0311 | 0.0035 | NA |
| BMI | Yengo | Publication supplement | rs1150659 | A | G | 0.2254 | -0.0139 | 0.0019 | NA |
| BMI | Yengo | Publication supplement | rs11538 | A | G | 0.8195 | -0.0135 | 0.0023 | NA |
| BMI | Yengo | Publication supplement | rs1154659 | A | G | 0.2112 | -0.0119 | 0.0022 | NA |
| BMI | Yengo | Publication supplement | rs11577094 | T | C | 0.0811 | 0.0182 | 0.003 | NA |
| BMI | Yengo | Publication supplement | rs11577179 | A | G | 0.3721 | -0.0108 | 0.0017 | NA |
| BMI | Yengo | Publication supplement | rs11581304 | T | C | 0.9032 | -0.0182 | 0.003 | NA |
| BMI | Yengo | Publication supplement | rs1158684 | A | G | 0.5088 | 0.0099 | 0.0017 | NA |
| BMI | Yengo | Publication supplement | rs1158805 | A | C | 0.3766 | -0.0137 | 0.0018 | NA |
| BMI | Yengo | Publication supplement | rs11604688 | T | C | 0.5607 | -0.0103 | 0.0017 | NA |
| BMI | Yengo | Publication supplement | rs11611246 | T | G | 0.21 | 0.024 | 0.002 | NA |
| BMI | Yengo | Publication supplement | rs11614340 | T | C | 0.6908 | -0.0133 | 0.0019 | NA |
| BMI | Yengo | Publication supplement | rs11629783 | C | G | 0.7692 | 0.0146 | 0.002 | NA |
| BMI | Yengo | Publication supplement | rs11635675 | T | G | 0.6465 | 0.0124 | 0.0018 | NA |
| BMI | Yengo | Publication supplement | rs11649864 | A | G | 0.0913 | 0.0178 | 0.0031 | NA |
| **Trait** | **GWAS author** | **Source** | **SNP** | **Effect Allele** | **Other Allele** | **EAF** | **beta** | **p** | **MR-Base/EBI Study ID** |
| BMI | Yengo | Publication supplement | rs11659764 | A | T | 0.053 | -0.0253 | 0.0039 | NA |
| BMI | Yengo | Publication supplement | rs11672660 | T | C | 0.2049 | -0.034 | 0.0021 | NA |
| BMI | Yengo | Publication supplement | rs1169091 | T | C | 0.2767 | -0.0113 | 0.002 | NA |
| BMI | Yengo | Publication supplement | rs11702843 | A | G | 0.2737 | 0.0114 | 0.002 | NA |
| BMI | Yengo | Publication supplement | rs11708733 | A | G | 0.0481 | -0.0227 | 0.0041 | NA |
| BMI | Yengo | Publication supplement | rs11713193 | A | G | 0.5073 | 0.0239 | 0.0017 | NA |
| BMI | Yengo | Publication supplement | rs11736228 | A | T | 0.7413 | 0.0139 | 0.002 | NA |
| BMI | Yengo | Publication supplement | rs11738695 | A | C | 0.586 | 0.0097 | 0.0017 | NA |
| BMI | Yengo | Publication supplement | rs11739877 | T | C | 0.6118 | 0.0117 | 0.0018 | NA |
| BMI | Yengo | Publication supplement | rs11753081 | T | G | 0.8251 | 0.0138 | 0.0021 | NA |
| BMI | Yengo | Publication supplement | rs11754747 | T | C | 0.2608 | 0.0116 | 0.0019 | NA |
| BMI | Yengo | Publication supplement | rs11781222 | T | C | 0.8714 | 0.0158 | 0.0024 | NA |
| BMI | Yengo | Publication supplement | rs11790280 | T | C | 0.6138 | -0.0103 | 0.0018 | NA |
| BMI | Yengo | Publication supplement | rs11792069 | A | G | 0.83 | 0.0145 | 0.0024 | NA |
| BMI | Yengo | Publication supplement | rs11792311 | A | G | 0.2388 | -0.0144 | 0.002 | NA |
| BMI | Yengo | Publication supplement | rs11855853 | T | C | 0.2649 | -0.0145 | 0.002 | NA |
| BMI | Yengo | Publication supplement | rs11866815 | T | C | 0.2461 | -0.0156 | 0.0019 | NA |
| BMI | Yengo | Publication supplement | rs1187352 | T | C | 0.3482 | -0.0119 | 0.0018 | NA |
| BMI | Yengo | Publication supplement | rs11880870 | A | G | 0.5199 | 0.0189 | 0.0017 | NA |
| BMI | Yengo | Publication supplement | rs11889536 | A | G | 0.8507 | 0.0189 | 0.0024 | NA |
| BMI | Yengo | Publication supplement | rs11904490 | A | C | 0.3911 | -0.0099 | 0.0018 | NA |
| BMI | Yengo | Publication supplement | rs11915371 | A | C | 0.7962 | -0.0149 | 0.0021 | NA |
| BMI | Yengo | Publication supplement | rs11917965 | A | C | 0.6085 | -0.0115 | 0.0018 | NA |
| BMI | Yengo | Publication supplement | rs11945861 | A | G | 0.2369 | -0.0148 | 0.002 | NA |
| BMI | Yengo | Publication supplement | rs11951673 | T | C | 0.3941 | -0.0123 | 0.0017 | NA |
| BMI | Yengo | Publication supplement | rs1199334 | A | G | 0.1877 | 0.0142 | 0.0021 | NA |
| BMI | Yengo | Publication supplement | rs12033257 | A | G | 0.6165 | 0.0146 | 0.0018 | NA |
| BMI | Yengo | Publication supplement | rs12035149 | C | G | 0.777 | -0.0146 | 0.0021 | NA |
| BMI | Yengo | Publication supplement | rs12041258 | T | C | 0.7713 | 0.0146 | 0.002 | NA |
| **Trait** | **GWAS author** | **Source** | **SNP** | **Effect Allele** | **Other Allele** | **EAF** | **beta** | **p** | **MR-Base/EBI Study ID** |
| BMI | Yengo | Publication supplement | rs12042959 | A | G | 0.8495 | 0.0144 | 0.0024 | NA |
| BMI | Yengo | Publication supplement | rs12065553 | A | G | 0.7074 | -0.0115 | 0.0019 | NA |
| BMI | Yengo | Publication supplement | rs12101393 | C | G | 0.7803 | 0.0127 | 0.0021 | NA |
| BMI | Yengo | Publication supplement | rs12147845 | T | C | 0.1135 | 0.0199 | 0.0027 | NA |
| BMI | Yengo | Publication supplement | rs12148330 | A | T | 0.181 | 0.0142 | 0.0022 | NA |
| BMI | Yengo | Publication supplement | rs12149756 | A | G | 0.2554 | -0.0125 | 0.0019 | NA |
| BMI | Yengo | Publication supplement | rs12150665 | T | C | 0.5942 | 0.0162 | 0.0017 | NA |
| BMI | Yengo | Publication supplement | rs1218822 | A | G | 0.6663 | 0.0168 | 0.0017 | NA |
| BMI | Yengo | Publication supplement | rs12189178 | T | C | 0.0378 | 0.0364 | 0.0046 | NA |
| BMI | Yengo | Publication supplement | rs12206564 | T | C | 0.505 | -0.0113 | 0.0017 | NA |
| BMI | Yengo | Publication supplement | rs1227244 | A | G | 0.6628 | -0.0106 | 0.0019 | NA |
| BMI | Yengo | Publication supplement | rs12299814 | A | C | 0.2525 | -0.0157 | 0.002 | NA |
| BMI | Yengo | Publication supplement | rs12325419 | A | G | 0.121 | -0.0188 | 0.0027 | NA |
| BMI | Yengo | Publication supplement | rs12364470 | T | G | 0.8374 | -0.0178 | 0.0022 | NA |
| BMI | Yengo | Publication supplement | rs12369179 | T | C | 0.0878 | -0.0359 | 0.0031 | NA |
| BMI | Yengo | Publication supplement | rs12380502 | C | G | 0.4266 | -0.0098 | 0.0017 | NA |
| BMI | Yengo | Publication supplement | rs1241986 | A | G | 0.8479 | -0.0139 | 0.0024 | NA |
| BMI | Yengo | Publication supplement | rs12422552 | C | G | 0.2663 | -0.0134 | 0.002 | NA |
| BMI | Yengo | Publication supplement | rs12429545 | A | G | 0.1248 | 0.0316 | 0.0025 | NA |
| BMI | Yengo | Publication supplement | rs12439798 | T | G | 0.4228 | 0.0125 | 0.0017 | NA |
| BMI | Yengo | Publication supplement | rs12439829 | A | T | 0.3857 | -0.0109 | 0.0018 | NA |
| BMI | Yengo | Publication supplement | rs12443621 | A | G | 0.5332 | -0.0096 | 0.0017 | NA |
| BMI | Yengo | Publication supplement | rs12446632 | A | G | 0.1424 | -0.0352 | 0.0024 | NA |
| BMI | Yengo | Publication supplement | rs12448257 | A | G | 0.218 | 0.0184 | 0.002 | NA |
| BMI | Yengo | Publication supplement | rs12448738 | A | C | 0.8629 | -0.0168 | 0.0025 | NA |
| BMI | Yengo | Publication supplement | rs12449219 | C | G | 0.8642 | -0.0155 | 0.0026 | NA |
| BMI | Yengo | Publication supplement | rs12454204 | T | G | 0.2299 | 0.0172 | 0.002 | NA |
| BMI | Yengo | Publication supplement | rs12454712 | T | C | 0.6296 | -0.0144 | 0.0018 | NA |
| BMI | Yengo | Publication supplement | rs12468863 | T | C | 0.5239 | -0.0153 | 0.0016 | NA |
| **Trait** | **GWAS author** | **Source** | **SNP** | **Effect Allele** | **Other Allele** | **EAF** | **beta** | **p** | **MR-Base/EBI Study ID** |
| BMI | Yengo | Publication supplement | rs12476772 | A | C | 0.3326 | 0.0123 | 0.0018 | NA |
| BMI | Yengo | Publication supplement | rs12519552 | A | G | 0.8449 | -0.0132 | 0.0024 | NA |
| BMI | Yengo | Publication supplement | rs12519652 | T | C | 0.6546 | 0.0094 | 0.0017 | NA |
| BMI | Yengo | Publication supplement | rs12564992 | A | G | 0.8856 | -0.0196 | 0.0026 | NA |
| BMI | Yengo | Publication supplement | rs12589208 | A | G | 0.8224 | -0.0126 | 0.0023 | NA |
| BMI | Yengo | Publication supplement | rs12593036 | A | G | 0.7007 | 0.0154 | 0.0019 | NA |
| BMI | Yengo | Publication supplement | rs12595158 | T | C | 0.0236 | -0.0394 | 0.0054 | NA |
| BMI | Yengo | Publication supplement | rs12597712 | C | G | 0.4201 | -0.0109 | 0.0017 | NA |
| BMI | Yengo | Publication supplement | rs12602912 | T | C | 0.2048 | 0.0176 | 0.0021 | NA |
| BMI | Yengo | Publication supplement | rs1260326 | T | C | 0.4027 | -0.0105 | 0.0017 | NA |
| BMI | Yengo | Publication supplement | rs12615778 | A | G | 0.6942 | 0.0104 | 0.0019 | NA |
| BMI | Yengo | Publication supplement | rs12620249 | A | G | 0.8501 | -0.0135 | 0.0025 | NA |
| BMI | Yengo | Publication supplement | rs12628891 | T | C | 0.3169 | -0.0112 | 0.0019 | NA |
| BMI | Yengo | Publication supplement | rs12629015 | A | G | 0.8148 | 0.0135 | 0.0023 | NA |
| BMI | Yengo | Publication supplement | rs12630999 | A | G | 0.7467 | 0.0175 | 0.0019 | NA |
| BMI | Yengo | Publication supplement | rs1263618 | T | C | 0.6858 | -0.012 | 0.0017 | NA |
| BMI | Yengo | Publication supplement | rs12651833 | C | G | 0.8049 | 0.013 | 0.0022 | NA |
| BMI | Yengo | Publication supplement | rs12675063 | A | T | 0.8869 | -0.0156 | 0.0026 | NA |
| BMI | Yengo | Publication supplement | rs12680842 | A | G | 0.6795 | 0.0133 | 0.0018 | NA |
| BMI | Yengo | Publication supplement | rs12705977 | T | G | 0.4551 | 0.0132 | 0.0017 | NA |
| BMI | Yengo | Publication supplement | rs1275691 | A | G | 0.4559 | -0.0094 | 0.0016 | NA |
| BMI | Yengo | Publication supplement | rs12759296 | T | G | 0.6891 | 0.0104 | 0.0018 | NA |
| BMI | Yengo | Publication supplement | rs12762034 | T | C | 0.9242 | -0.024 | 0.0032 | NA |
| BMI | Yengo | Publication supplement | rs12776880 | A | T | 0.6828 | 0.0128 | 0.0019 | NA |
| BMI | Yengo | Publication supplement | rs1277723 | A | G | 0.7756 | 0.0113 | 0.002 | NA |
| BMI | Yengo | Publication supplement | rs12779328 | T | C | 0.2833 | 0.0105 | 0.0019 | NA |
| BMI | Yengo | Publication supplement | rs1285245 | C | G | 0.3669 | -0.0121 | 0.0018 | NA |
| BMI | Yengo | Publication supplement | rs12868881 | A | T | 0.42 | 0.0128 | 0.0017 | NA |
| BMI | Yengo | Publication supplement | rs12885454 | A | C | 0.3431 | -0.0185 | 0.0017 | NA |
| **Trait** | **GWAS author** | **Source** | **SNP** | **Effect Allele** | **Other Allele** | **EAF** | **beta** | **p** | **MR-Base/EBI Study ID** |
| BMI | Yengo | Publication supplement | rs12888545 | A | G | 0.7481 | -0.0136 | 0.002 | NA |
| BMI | Yengo | Publication supplement | rs12905439 | C | G | 0.6607 | 0.0118 | 0.0018 | NA |
| BMI | Yengo | Publication supplement | rs12922346 | C | G | 0.2657 | 0.0136 | 0.002 | NA |
| BMI | Yengo | Publication supplement | rs12939549 | A | G | 0.5665 | 0.018 | 0.0016 | NA |
| BMI | Yengo | Publication supplement | rs12953970 | A | G | 0.1833 | 0.0125 | 0.0023 | NA |
| BMI | Yengo | Publication supplement | rs1296328 | A | C | 0.4343 | 0.0179 | 0.0018 | NA |
| BMI | Yengo | Publication supplement | rs12964689 | A | G | 0.5176 | 0.0203 | 0.0017 | NA |
| BMI | Yengo | Publication supplement | rs12989476 | T | C | 0.3521 | 0.013 | 0.0018 | NA |
| BMI | Yengo | Publication supplement | rs13001304 | T | C | 0.6881 | 0.0104 | 0.0019 | NA |
| BMI | Yengo | Publication supplement | rs13012099 | A | G | 0.3406 | -0.014 | 0.0018 | NA |
| BMI | Yengo | Publication supplement | rs13021737 | A | G | 0.1681 | -0.0574 | 0.0021 | NA |
| BMI | Yengo | Publication supplement | rs13034320 | C | G | 0.2589 | 0.0114 | 0.0021 | NA |
| BMI | Yengo | Publication supplement | rs1304070 | A | G | 0.7615 | 0.0126 | 0.002 | NA |
| BMI | Yengo | Publication supplement | rs13047416 | C | G | 0.6231 | 0.0154 | 0.0018 | NA |
| BMI | Yengo | Publication supplement | rs13063194 | T | C | 0.7938 | -0.0144 | 0.0021 | NA |
| BMI | Yengo | Publication supplement | rs13072095 | T | C | 0.6656 | 0.0095 | 0.0017 | NA |
| BMI | Yengo | Publication supplement | rs13105058 | T | C | 0.3248 | 0.0109 | 0.0018 | NA |
| BMI | Yengo | Publication supplement | rs13107325 | T | C | 0.0737 | 0.047 | 0.0032 | NA |
| BMI | Yengo | Publication supplement | rs13110266 | A | G | 0.4065 | -0.0117 | 0.0017 | NA |
| BMI | Yengo | Publication supplement | rs13153166 | A | C | 0.9548 | 0.0232 | 0.004 | NA |
| BMI | Yengo | Publication supplement | rs13159555 | A | G | 0.7863 | -0.0119 | 0.0021 | NA |
| BMI | Yengo | Publication supplement | rs13168288 | A | G | 0.7497 | 0.0133 | 0.002 | NA |
| BMI | Yengo | Publication supplement | rs13174863 | A | G | 0.8452 | -0.0192 | 0.0023 | NA |
| BMI | Yengo | Publication supplement | rs13184896 | T | G | 0.4346 | -0.0133 | 0.0016 | NA |
| BMI | Yengo | Publication supplement | rs13191362 | A | G | 0.8802 | 0.0236 | 0.0025 | NA |
| BMI | Yengo | Publication supplement | rs13201877 | A | G | 0.8601 | -0.0152 | 0.0024 | NA |
| BMI | Yengo | Publication supplement | rs1320903 | A | G | 0.3174 | 0.0216 | 0.0018 | NA |
| BMI | Yengo | Publication supplement | rs13209872 | C | G | 0.3403 | -0.0152 | 0.0018 | NA |
| BMI | Yengo | Publication supplement | rs13209968 | C | G | 0.5177 | 0.0114 | 0.0017 | NA |
| **Trait** | **GWAS author** | **Source** | **SNP** | **Effect Allele** | **Other Allele** | **EAF** | **beta** | **p** | **MR-Base/EBI Study ID** |
| BMI | Yengo | Publication supplement | rs13227658 | T | C | 0.5534 | -0.0157 | 0.0017 | NA |
| BMI | Yengo | Publication supplement | rs13250058 | T | G | 0.6771 | 0.0112 | 0.0018 | NA |
| BMI | Yengo | Publication supplement | rs13263601 | A | C | 0.6522 | -0.0154 | 0.0018 | NA |
| BMI | Yengo | Publication supplement | rs13266989 | C | G | 0.5986 | 0.011 | 0.0018 | NA |
| BMI | Yengo | Publication supplement | rs13267015 | T | C | 0.2788 | 0.0105 | 0.0019 | NA |
| BMI | Yengo | Publication supplement | rs13290794 | A | G | 0.3582 | -0.0141 | 0.0018 | NA |
| BMI | Yengo | Publication supplement | rs13292976 | T | C | 0.4458 | 0.0131 | 0.0017 | NA |
| BMI | Yengo | Publication supplement | rs13298062 | A | G | 0.798 | 0.0137 | 0.0022 | NA |
| BMI | Yengo | Publication supplement | rs1330052 | C | G | 0.6496 | -0.0132 | 0.0018 | NA |
| BMI | Yengo | Publication supplement | rs13329567 | T | C | 0.2308 | -0.0293 | 0.002 | NA |
| BMI | Yengo | Publication supplement | rs1336486 | T | G | 0.6795 | -0.0141 | 0.0018 | NA |
| BMI | Yengo | Publication supplement | rs13380104 | T | C | 0.4179 | -0.0136 | 0.0017 | NA |
| BMI | Yengo | Publication supplement | rs13417156 | T | C | 0.5639 | -0.0144 | 0.0017 | NA |
| BMI | Yengo | Publication supplement | rs13425435 | A | C | 0.6674 | 0.012 | 0.0018 | NA |
| BMI | Yengo | Publication supplement | rs13432055 | T | C | 0.7141 | -0.0117 | 0.0018 | NA |
| BMI | Yengo | Publication supplement | rs1345942 | T | C | 0.6282 | -0.0104 | 0.0018 | NA |
| BMI | Yengo | Publication supplement | rs1358980 | T | C | 0.4777 | -0.0125 | 0.0017 | NA |
| BMI | Yengo | Publication supplement | rs1361739 | A | G | 0.3264 | -0.0176 | 0.0018 | NA |
| BMI | Yengo | Publication supplement | rs1363695 | T | C | 0.2322 | -0.013 | 0.0021 | NA |
| BMI | Yengo | Publication supplement | rs1365466 | T | C | 0.7406 | -0.0137 | 0.0019 | NA |
| BMI | Yengo | Publication supplement | rs1371108 | A | C | 0.3247 | 0.0119 | 0.0018 | NA |
| BMI | Yengo | Publication supplement | rs138289 | A | T | 0.5171 | 0.0103 | 0.0017 | NA |
| BMI | Yengo | Publication supplement | rs1394879 | C | G | 0.417 | 0.0101 | 0.0017 | NA |
| BMI | Yengo | Publication supplement | rs1399471 | C | G | 0.2608 | -0.0131 | 0.002 | NA |
| BMI | Yengo | Publication supplement | rs1402025 | T | C | 0.7733 | -0.0121 | 0.0021 | NA |
| BMI | Yengo | Publication supplement | rs1409818 | T | C | 0.1156 | 0.0201 | 0.0029 | NA |
| BMI | Yengo | Publication supplement | rs1412235 | C | G | 0.3175 | 0.0246 | 0.0017 | NA |
| BMI | Yengo | Publication supplement | rs1420341 | T | C | 0.8202 | -0.0138 | 0.0023 | NA |
| BMI | Yengo | Publication supplement | rs1421334 | A | C | 0.4569 | 0.0125 | 0.0018 | NA |
| **Trait** | **GWAS author** | **Source** | **SNP** | **Effect Allele** | **Other Allele** | **EAF** | **beta** | **p** | **MR-Base/EBI Study ID** |
| BMI | Yengo | Publication supplement | rs1430387 | T | C | 0.5705 | 0.0114 | 0.0017 | NA |
| BMI | Yengo | Publication supplement | rs1436344 | C | G | 0.5922 | 0.0141 | 0.0017 | NA |
| BMI | Yengo | Publication supplement | rs1445305 | A | G | 0.1045 | -0.0161 | 0.0029 | NA |
| BMI | Yengo | Publication supplement | rs1452075 | T | C | 0.7277 | 0.0141 | 0.0018 | NA |
| BMI | Yengo | Publication supplement | rs1454687 | C | G | 0.4773 | 0.0202 | 0.0017 | NA |
| BMI | Yengo | Publication supplement | rs1455137 | A | C | 0.6197 | -0.0106 | 0.0017 | NA |
| BMI | Yengo | Publication supplement | rs1465900 | A | C | 0.7812 | 0.0125 | 0.002 | NA |
| BMI | Yengo | Publication supplement | rs1467693 | A | T | 0.2916 | 0.0114 | 0.0019 | NA |
| BMI | Yengo | Publication supplement | rs1476322 | A | G | 0.569 | 0.0101 | 0.0017 | NA |
| BMI | Yengo | Publication supplement | rs1477199 | A | G | 0.8549 | -0.0228 | 0.0024 | NA |
| BMI | Yengo | Publication supplement | rs1485038 | T | C | 0.7965 | 0.0143 | 0.0022 | NA |
| BMI | Yengo | Publication supplement | rs1496742 | A | G | 0.5156 | 0.0094 | 0.0017 | NA |
| BMI | Yengo | Publication supplement | rs1503526 | T | C | 0.5162 | -0.014 | 0.0017 | NA |
| BMI | Yengo | Publication supplement | rs1511471 | A | G | 0.3174 | 0.0113 | 0.0019 | NA |
| BMI | Yengo | Publication supplement | rs1512914 | T | G | 0.6111 | -0.0126 | 0.0018 | NA |
| BMI | Yengo | Publication supplement | rs1521527 | C | G | 0.5324 | -0.0121 | 0.0017 | NA |
| BMI | Yengo | Publication supplement | rs1522569 | T | G | 0.8181 | 0.0164 | 0.0022 | NA |
| BMI | Yengo | Publication supplement | rs1523768 | A | G | 0.6798 | -0.011 | 0.0017 | NA |
| BMI | Yengo | Publication supplement | rs1524277 | T | C | 0.519 | -0.0106 | 0.0018 | NA |
| BMI | Yengo | Publication supplement | rs1526665 | T | C | 0.4065 | -0.0117 | 0.0018 | NA |
| BMI | Yengo | Publication supplement | rs1528435 | T | C | 0.6331 | 0.0164 | 0.0017 | NA |
| BMI | Yengo | Publication supplement | rs1544459 | T | C | 0.5464 | -0.0103 | 0.0016 | NA |
| BMI | Yengo | Publication supplement | rs1554194 | C | G | 0.5288 | 0.0103 | 0.0017 | NA |
| BMI | Yengo | Publication supplement | rs1554790 | C | G | 0.5181 | -0.0129 | 0.0017 | NA |
| BMI | Yengo | Publication supplement | rs155510 | T | G | 0.8088 | 0.0165 | 0.0021 | NA |
| BMI | Yengo | Publication supplement | rs1584121 | A | G | 0.1907 | -0.0126 | 0.0022 | NA |
| BMI | Yengo | Publication supplement | rs159032 | T | C | 0.2439 | 0.0129 | 0.002 | NA |
| BMI | Yengo | Publication supplement | rs1596299 | T | C | 0.6815 | 0.0102 | 0.0019 | NA |
| BMI | Yengo | Publication supplement | rs1608445 | A | G | 0.435 | -0.0104 | 0.0018 | NA |
| **Trait** | **GWAS author** | **Source** | **SNP** | **Effect Allele** | **Other Allele** | **EAF** | **beta** | **p** | **MR-Base/EBI Study ID** |
| BMI | Yengo | Publication supplement | rs1625427 | T | C | 0.6504 | 0.013 | 0.0018 | NA |
| BMI | Yengo | Publication supplement | rs1657930 | A | G | 0.8028 | -0.0123 | 0.0022 | NA |
| BMI | Yengo | Publication supplement | rs1658820 | T | G | 0.2468 | 0.0141 | 0.0021 | NA |
| BMI | Yengo | Publication supplement | rs1668633 | T | C | 0.5738 | 0.01 | 0.0017 | NA |
| BMI | Yengo | Publication supplement | rs16833232 | T | C | 0.3123 | -0.0109 | 0.0019 | NA |
| BMI | Yengo | Publication supplement | rs16846136 | A | C | 0.2677 | 0.0129 | 0.0019 | NA |
| BMI | Yengo | Publication supplement | rs16851483 | T | G | 0.0693 | 0.0369 | 0.0035 | NA |
| BMI | Yengo | Publication supplement | rs16867703 | T | G | 0.6344 | -0.0137 | 0.0018 | NA |
| BMI | Yengo | Publication supplement | rs16871902 | A | G | 0.4877 | 0.0125 | 0.0017 | NA |
| BMI | Yengo | Publication supplement | rs16882001 | A | G | 0.9494 | -0.0269 | 0.004 | NA |
| BMI | Yengo | Publication supplement | rs1689437 | A | G | 0.9335 | -0.0251 | 0.0034 | NA |
| BMI | Yengo | Publication supplement | rs16903285 | T | C | 0.8593 | -0.0331 | 0.0026 | NA |
| BMI | Yengo | Publication supplement | rs16906845 | A | G | 0.0668 | -0.0225 | 0.0038 | NA |
| BMI | Yengo | Publication supplement | rs16907751 | T | C | 0.1047 | -0.0209 | 0.003 | NA |
| BMI | Yengo | Publication supplement | rs16916303 | A | G | 0.8752 | 0.0156 | 0.0028 | NA |
| BMI | Yengo | Publication supplement | rs16932761 | A | G | 0.2542 | -0.014 | 0.002 | NA |
| BMI | Yengo | Publication supplement | rs16940823 | A | C | 0.1905 | -0.0146 | 0.0023 | NA |
| BMI | Yengo | Publication supplement | rs16942944 | A | G | 0.2953 | 0.0108 | 0.0019 | NA |
| BMI | Yengo | Publication supplement | rs16943356 | A | G | 0.0678 | -0.0198 | 0.0033 | NA |
| BMI | Yengo | Publication supplement | rs16965225 | T | G | 0.0674 | 0.0216 | 0.0034 | NA |
| BMI | Yengo | Publication supplement | rs16966801 | A | G | 0.7984 | -0.0156 | 0.0022 | NA |
| BMI | Yengo | Publication supplement | rs16982345 | A | G | 0.2487 | 0.0122 | 0.002 | NA |
| BMI | Yengo | Publication supplement | rs17001561 | A | G | 0.1573 | 0.0151 | 0.0023 | NA |
| BMI | Yengo | Publication supplement | rs17014375 | T | G | 0.8652 | -0.0172 | 0.0025 | NA |
| BMI | Yengo | Publication supplement | rs17019087 | T | C | 0.6397 | -0.0109 | 0.0018 | NA |
| BMI | Yengo | Publication supplement | rs17056301 | T | C | 0.7364 | -0.0118 | 0.002 | NA |
| BMI | Yengo | Publication supplement | rs17066842 | A | G | 0.0406 | -0.0676 | 0.0042 | NA |
| BMI | Yengo | Publication supplement | rs17069831 | T | C | 0.2795 | -0.0109 | 0.0019 | NA |
| BMI | Yengo | Publication supplement | rs1707322 | A | G | 0.2937 | -0.0128 | 0.0018 | NA |
| **Trait** | **GWAS author** | **Source** | **SNP** | **Effect Allele** | **Other Allele** | **EAF** | **beta** | **p** | **MR-Base/EBI Study ID** |
| BMI | Yengo | Publication supplement | rs17094222 | T | C | 0.7961 | -0.0181 | 0.002 | NA |
| BMI | Yengo | Publication supplement | rs17105272 | T | C | 0.3186 | 0.011 | 0.0019 | NA |
| BMI | Yengo | Publication supplement | rs17120344 | A | G | 0.0592 | 0.0224 | 0.0037 | NA |
| BMI | Yengo | Publication supplement | rs17201143 | T | C | 0.3124 | -0.0103 | 0.0019 | NA |
| BMI | Yengo | Publication supplement | rs17203016 | A | G | 0.804 | -0.015 | 0.002 | NA |
| BMI | Yengo | Publication supplement | rs17207196 | T | C | 0.4118 | -0.0221 | 0.0018 | NA |
| BMI | Yengo | Publication supplement | rs17236194 | T | C | 0.8655 | -0.0153 | 0.0024 | NA |
| BMI | Yengo | Publication supplement | rs17272434 | A | G | 0.312 | -0.0105 | 0.0018 | NA |
| BMI | Yengo | Publication supplement | rs1730859 | A | G | 0.6584 | -0.0118 | 0.0017 | NA |
| BMI | Yengo | Publication supplement | rs17309825 | T | C | 0.9602 | -0.0312 | 0.0042 | NA |
| BMI | Yengo | Publication supplement | rs17327461 | T | C | 0.4498 | 0.0125 | 0.0016 | NA |
| BMI | Yengo | Publication supplement | rs17387761 | A | G | 0.278 | -0.0106 | 0.0019 | NA |
| BMI | Yengo | Publication supplement | rs17391694 | T | C | 0.1189 | 0.0317 | 0.0025 | NA |
| BMI | Yengo | Publication supplement | rs17405819 | T | C | 0.699 | 0.0215 | 0.0018 | NA |
| BMI | Yengo | Publication supplement | rs17424278 | A | C | 0.8946 | -0.017 | 0.0028 | NA |
| BMI | Yengo | Publication supplement | rs17446091 | T | C | 0.7926 | -0.0123 | 0.002 | NA |
| BMI | Yengo | Publication supplement | rs17448885 | C | G | 0.6537 | 0.0105 | 0.0018 | NA |
| BMI | Yengo | Publication supplement | rs17450772 | T | C | 0.8875 | -0.0189 | 0.003 | NA |
| BMI | Yengo | Publication supplement | rs17513613 | T | C | 0.6764 | -0.0186 | 0.0018 | NA |
| BMI | Yengo | Publication supplement | rs175165 | T | G | 0.6059 | 0.0103 | 0.0018 | NA |
| BMI | Yengo | Publication supplement | rs17531363 | A | C | 0.6967 | 0.0133 | 0.0019 | NA |
| BMI | Yengo | Publication supplement | rs17535749 | A | G | 0.1023 | 0.015 | 0.0027 | NA |
| BMI | Yengo | Publication supplement | rs17538472 | T | C | 0.1908 | 0.0129 | 0.0022 | NA |
| BMI | Yengo | Publication supplement | rs17551974 | A | C | 0.1782 | -0.0141 | 0.0022 | NA |
| BMI | Yengo | Publication supplement | rs17573940 | A | G | 0.7081 | -0.0113 | 0.0018 | NA |
| BMI | Yengo | Publication supplement | rs17599948 | A | G | 0.8331 | 0.013 | 0.0022 | NA |
| BMI | Yengo | Publication supplement | rs17608150 | T | C | 0.0754 | 0.0196 | 0.0031 | NA |
| BMI | Yengo | Publication supplement | rs17619973 | A | G | 0.9254 | 0.02 | 0.0032 | NA |
| BMI | Yengo | Publication supplement | rs17636031 | T | C | 0.7299 | -0.016 | 0.0019 | NA |
| **Trait** | **GWAS author** | **Source** | **SNP** | **Effect Allele** | **Other Allele** | **EAF** | **beta** | **p** | **MR-Base/EBI Study ID** |
| BMI | Yengo | Publication supplement | rs17681708 | T | C | 0.6877 | -0.0106 | 0.0018 | NA |
| BMI | Yengo | Publication supplement | rs17709991 | T | C | 0.7319 | -0.011 | 0.0018 | NA |
| BMI | Yengo | Publication supplement | rs17720922 | T | C | 0.19 | 0.0131 | 0.0022 | NA |
| BMI | Yengo | Publication supplement | rs17724992 | A | G | 0.7404 | 0.0183 | 0.0019 | NA |
| BMI | Yengo | Publication supplement | rs17757975 | T | C | 0.8518 | 0.0143 | 0.0024 | NA |
| BMI | Yengo | Publication supplement | rs17767510 | C | G | 0.1697 | 0.0129 | 0.0023 | NA |
| BMI | Yengo | Publication supplement | rs17776719 | A | G | 0.854 | -0.0167 | 0.0025 | NA |
| BMI | Yengo | Publication supplement | rs17789218 | T | C | 0.7608 | -0.013 | 0.0019 | NA |
| BMI | Yengo | Publication supplement | rs17806379 | T | C | 0.1789 | -0.0258 | 0.0022 | NA |
| BMI | Yengo | Publication supplement | rs17820822 | T | G | 0.6446 | 0.0143 | 0.0018 | NA |
| BMI | Yengo | Publication supplement | rs1784460 | A | T | 0.4035 | 0.0132 | 0.0018 | NA |
| BMI | Yengo | Publication supplement | rs1787267 | C | G | 0.0627 | -0.0237 | 0.0036 | NA |
| BMI | Yengo | Publication supplement | rs1814170 | A | T | 0.8946 | 0.0203 | 0.0029 | NA |
| BMI | Yengo | Publication supplement | rs1829130 | T | C | 0.678 | -0.011 | 0.0019 | NA |
| BMI | Yengo | Publication supplement | rs1830074 | T | C | 0.712 | -0.0115 | 0.0019 | NA |
| BMI | Yengo | Publication supplement | rs1840969 | A | T | 0.591 | -0.0106 | 0.0018 | NA |
| BMI | Yengo | Publication supplement | rs1852006 | A | G | 0.3519 | -0.0156 | 0.0018 | NA |
| BMI | Yengo | Publication supplement | rs1853639 | A | G | 0.6363 | -0.0111 | 0.0018 | NA |
| BMI | Yengo | Publication supplement | rs1863652 | A | G | 0.3449 | -0.0115 | 0.0018 | NA |
| BMI | Yengo | Publication supplement | rs1865341 | T | C | 0.758 | 0.0128 | 0.002 | NA |
| BMI | Yengo | Publication supplement | rs1865989 | A | T | 0.37 | -0.01 | 0.0018 | NA |
| BMI | Yengo | Publication supplement | rs1876359 | T | C | 0.3604 | 0.013 | 0.0017 | NA |
| BMI | Yengo | Publication supplement | rs1884389 | T | C | 0.4289 | -0.0103 | 0.0017 | NA |
| BMI | Yengo | Publication supplement | rs1884897 | A | G | 0.3692 | -0.0194 | 0.0017 | NA |
| BMI | Yengo | Publication supplement | rs1885728 | A | G | 0.6787 | 0.0108 | 0.0019 | NA |
| BMI | Yengo | Publication supplement | rs1895957 | T | G | 0.2219 | -0.0171 | 0.0021 | NA |
| BMI | Yengo | Publication supplement | rs189843 | C | G | 0.5557 | -0.0098 | 0.0017 | NA |
| BMI | Yengo | Publication supplement | rs1899689 | T | C | 0.3986 | 0.0117 | 0.0016 | NA |
| BMI | Yengo | Publication supplement | rs1899898 | T | C | 0.6799 | 0.0113 | 0.0018 | NA |
| **Trait** | **GWAS author** | **Source** | **SNP** | **Effect Allele** | **Other Allele** | **EAF** | **beta** | **p** | **MR-Base/EBI Study ID** |
| BMI | Yengo | Publication supplement | rs1903579 | C | G | 0.5502 | 0.0111 | 0.0017 | NA |
| BMI | Yengo | Publication supplement | rs1912631 | A | G | 0.4088 | -0.0119 | 0.0018 | NA |
| BMI | Yengo | Publication supplement | rs1927790 | T | C | 0.5891 | -0.0148 | 0.0016 | NA |
| BMI | Yengo | Publication supplement | rs1928295 | T | C | 0.5539 | 0.0141 | 0.0016 | NA |
| BMI | Yengo | Publication supplement | rs1937684 | A | T | 0.6592 | 0.0112 | 0.0018 | NA |
| BMI | Yengo | Publication supplement | rs1941213 | A | C | 0.7236 | 0.0117 | 0.002 | NA |
| BMI | Yengo | Publication supplement | rs1941697 | A | G | 0.4538 | 0.0123 | 0.0017 | NA |
| BMI | Yengo | Publication supplement | rs1951455 | T | C | 0.2747 | -0.0145 | 0.0019 | NA |
| BMI | Yengo | Publication supplement | rs1955540 | T | C | 0.1922 | -0.0158 | 0.0022 | NA |
| BMI | Yengo | Publication supplement | rs1956153 | A | T | 0.8213 | -0.013 | 0.0022 | NA |
| BMI | Yengo | Publication supplement | rs1964927 | A | G | 0.3617 | -0.0124 | 0.0018 | NA |
| BMI | Yengo | Publication supplement | rs1982441 | T | G | 0.1381 | 0.0175 | 0.0026 | NA |
| BMI | Yengo | Publication supplement | rs1993709 | A | G | 0.1823 | -0.0331 | 0.0021 | NA |
| BMI | Yengo | Publication supplement | rs2009416 | T | C | 0.361 | -0.0121 | 0.0018 | NA |
| BMI | Yengo | Publication supplement | rs2010281 | A | G | 0.3552 | -0.0161 | 0.0017 | NA |
| BMI | Yengo | Publication supplement | rs2012502 | A | C | 0.3717 | 0.0113 | 0.0017 | NA |
| BMI | Yengo | Publication supplement | rs2023671 | T | G | 0.7479 | -0.0122 | 0.002 | NA |
| BMI | Yengo | Publication supplement | rs2029331 | C | G | 0.2447 | -0.014 | 0.002 | NA |
| BMI | Yengo | Publication supplement | rs2033529 | A | G | 0.7064 | -0.0205 | 0.0018 | NA |
| BMI | Yengo | Publication supplement | rs2035831 | C | G | 0.339 | -0.0108 | 0.0018 | NA |
| BMI | Yengo | Publication supplement | rs2051559 | T | C | 0.8692 | -0.0176 | 0.0026 | NA |
| BMI | Yengo | Publication supplement | rs2063177 | A | G | 0.3541 | 0.012 | 0.0018 | NA |
| BMI | Yengo | Publication supplement | rs2065418 | T | G | 0.6377 | 0.0166 | 0.0018 | NA |
| BMI | Yengo | Publication supplement | rs2074881 | T | C | 0.1243 | -0.0155 | 0.0024 | NA |
| BMI | Yengo | Publication supplement | rs2075650 | A | G | 0.8606 | 0.0244 | 0.0023 | NA |
| BMI | Yengo | Publication supplement | rs208015 | T | C | 0.0784 | 0.0356 | 0.0034 | NA |
| BMI | Yengo | Publication supplement | rs2080454 | A | C | 0.6211 | -0.0129 | 0.0017 | NA |
| BMI | Yengo | Publication supplement | rs2100814 | A | G | 0.4103 | 0.0113 | 0.0017 | NA |
| BMI | Yengo | Publication supplement | rs2119753 | A | G | 0.611 | 0.0102 | 0.0018 | NA |
| **Trait** | **GWAS author** | **Source** | **SNP** | **Effect Allele** | **Other Allele** | **EAF** | **beta** | **p** | **MR-Base/EBI Study ID** |
| BMI | Yengo | Publication supplement | rs2122042 | T | G | 0.2056 | 0.0235 | 0.002 | NA |
| BMI | Yengo | Publication supplement | rs213533 | A | C | 0.857 | -0.0156 | 0.0025 | NA |
| BMI | Yengo | Publication supplement | rs2143253 | A | G | 0.1189 | -0.0188 | 0.0026 | NA |
| BMI | Yengo | Publication supplement | rs2143624 | A | G | 0.3682 | 0.0098 | 0.0017 | NA |
| BMI | Yengo | Publication supplement | rs2155645 | T | C | 0.2608 | -0.0116 | 0.0019 | NA |
| BMI | Yengo | Publication supplement | rs215634 | A | G | 0.3788 | 0.0152 | 0.0018 | NA |
| BMI | Yengo | Publication supplement | rs2160077 | A | G | 0.4294 | 0.0093 | 0.0016 | NA |
| BMI | Yengo | Publication supplement | rs2161097 | T | C | 0.4436 | 0.0096 | 0.0017 | NA |
| BMI | Yengo | Publication supplement | rs2162524 | T | C | 0.6679 | -0.0155 | 0.0018 | NA |
| BMI | Yengo | Publication supplement | rs2163188 | C | G | 0.474 | 0.0131 | 0.0017 | NA |
| BMI | Yengo | Publication supplement | rs2170382 | T | C | 0.1203 | 0.0172 | 0.0027 | NA |
| BMI | Yengo | Publication supplement | rs2172211 | A | G | 0.7392 | -0.0101 | 0.0018 | NA |
| BMI | Yengo | Publication supplement | rs2174307 | C | G | 0.4067 | 0.0121 | 0.0017 | NA |
| BMI | Yengo | Publication supplement | rs217433 | T | C | 0.7947 | -0.0117 | 0.0021 | NA |
| BMI | Yengo | Publication supplement | rs2174367 | T | G | 0.3136 | -0.0121 | 0.0019 | NA |
| BMI | Yengo | Publication supplement | rs217671 | A | G | 0.7281 | -0.0144 | 0.0019 | NA |
| BMI | Yengo | Publication supplement | rs2185027 | A | C | 0.7032 | -0.0135 | 0.0018 | NA |
| BMI | Yengo | Publication supplement | rs2190788 | T | G | 0.3106 | 0.0141 | 0.0019 | NA |
| BMI | Yengo | Publication supplement | rs2192158 | A | G | 0.4601 | 0.0129 | 0.0017 | NA |
| BMI | Yengo | Publication supplement | rs2195086 | T | G | 0.8411 | -0.0133 | 0.0023 | NA |
| BMI | Yengo | Publication supplement | rs2198679 | A | G | 0.5324 | 0.0107 | 0.0017 | NA |
| BMI | Yengo | Publication supplement | rs2224272 | T | G | 0.2278 | 0.0118 | 0.0021 | NA |
| BMI | Yengo | Publication supplement | rs2228213 | A | G | 0.3481 | -0.0139 | 0.0017 | NA |
| BMI | Yengo | Publication supplement | rs223051 | T | C | 0.6759 | 0.0112 | 0.0018 | NA |
| BMI | Yengo | Publication supplement | rs223391 | A | G | 0.1947 | -0.0131 | 0.0021 | NA |
| BMI | Yengo | Publication supplement | rs2233984 | T | C | 0.0686 | -0.0184 | 0.0033 | NA |
| BMI | Yengo | Publication supplement | rs2235564 | T | C | 0.3466 | 0.0131 | 0.0018 | NA |
| BMI | Yengo | Publication supplement | rs2246012 | T | C | 0.8372 | -0.0158 | 0.0022 | NA |
| BMI | Yengo | Publication supplement | rs2246664 | A | G | 0.3661 | 0.0137 | 0.0018 | NA |
| **Trait** | **GWAS author** | **Source** | **SNP** | **Effect Allele** | **Other Allele** | **EAF** | **beta** | **p** | **MR-Base/EBI Study ID** |
| BMI | Yengo | Publication supplement | rs2254542 | A | C | 0.0423 | -0.0241 | 0.004 | NA |
| BMI | Yengo | Publication supplement | rs2270778 | T | C | 0.5839 | -0.0097 | 0.0017 | NA |
| BMI | Yengo | Publication supplement | rs2271189 | A | G | 0.4051 | -0.0144 | 0.0018 | NA |
| BMI | Yengo | Publication supplement | rs2274550 | T | C | 0.1402 | -0.014 | 0.0025 | NA |
| BMI | Yengo | Publication supplement | rs2279620 | C | G | 0.1039 | 0.017 | 0.0028 | NA |
| BMI | Yengo | Publication supplement | rs2281819 | A | T | 0.2302 | -0.016 | 0.002 | NA |
| BMI | Yengo | Publication supplement | rs2282231 | T | C | 0.2249 | 0.0165 | 0.0021 | NA |
| BMI | Yengo | Publication supplement | rs2282802 | A | G | 0.5554 | 0.0109 | 0.0017 | NA |
| BMI | Yengo | Publication supplement | rs2283093 | T | C | 0.2066 | 0.0127 | 0.0021 | NA |
| BMI | Yengo | Publication supplement | rs2289379 | T | C | 0.398 | -0.0135 | 0.0018 | NA |
| BMI | Yengo | Publication supplement | rs2293605 | T | C | 0.1307 | -0.0167 | 0.0027 | NA |
| BMI | Yengo | Publication supplement | rs2304130 | A | G | 0.9147 | 0.0176 | 0.003 | NA |
| BMI | Yengo | Publication supplement | rs2307111 | T | C | 0.6038 | 0.0265 | 0.0016 | NA |
| BMI | Yengo | Publication supplement | rs2317299 | T | C | 0.4403 | 0.0106 | 0.0017 | NA |
| BMI | Yengo | Publication supplement | rs2322622 | T | C | 0.6299 | -0.0094 | 0.0017 | NA |
| BMI | Yengo | Publication supplement | rs2357760 | A | G | 0.6754 | 0.0145 | 0.0017 | NA |
| BMI | Yengo | Publication supplement | rs2362777 | T | G | 0.3614 | -0.0102 | 0.0018 | NA |
| BMI | Yengo | Publication supplement | rs236527 | C | G | 0.6956 | -0.0107 | 0.0019 | NA |
| BMI | Yengo | Publication supplement | rs2365389 | T | C | 0.4143 | -0.0174 | 0.0017 | NA |
| BMI | Yengo | Publication supplement | rs2367112 | T | G | 0.5081 | 0.0119 | 0.0016 | NA |
| BMI | Yengo | Publication supplement | rs2371767 | C | G | 0.2769 | 0.0107 | 0.0018 | NA |
| BMI | Yengo | Publication supplement | rs2396625 | A | T | 0.4338 | -0.0162 | 0.0018 | NA |
| BMI | Yengo | Publication supplement | rs2423668 | T | C | 0.4495 | 0.0105 | 0.0019 | NA |
| BMI | Yengo | Publication supplement | rs2425840 | A | C | 0.5941 | -0.0119 | 0.0018 | NA |
| BMI | Yengo | Publication supplement | rs2429150 | A | C | 0.5836 | -0.0111 | 0.0018 | NA |
| BMI | Yengo | Publication supplement | rs243387 | A | G | 0.4578 | -0.0101 | 0.0017 | NA |
| BMI | Yengo | Publication supplement | rs2440452 | T | G | 0.1339 | 0.0142 | 0.0026 | NA |
| BMI | Yengo | Publication supplement | rs2448241 | A | G | 0.0971 | -0.0176 | 0.003 | NA |
| BMI | Yengo | Publication supplement | rs2450444 | A | G | 0.349 | -0.0107 | 0.0017 | NA |
| **Trait** | **GWAS author** | **Source** | **SNP** | **Effect Allele** | **Other Allele** | **EAF** | **beta** | **p** | **MR-Base/EBI Study ID** |
| BMI | Yengo | Publication supplement | rs2451746 | C | G | 0.631 | 0.0111 | 0.0018 | NA |
| BMI | Yengo | Publication supplement | rs2467594 | A | G | 0.3635 | -0.0122 | 0.0018 | NA |
| BMI | Yengo | Publication supplement | rs2470520 | T | C | 0.6034 | -0.0109 | 0.0017 | NA |
| BMI | Yengo | Publication supplement | rs2477017 | A | G | 0.3813 | 0.0104 | 0.0018 | NA |
| BMI | Yengo | Publication supplement | rs2478879 | A | G | 0.6054 | 0.0159 | 0.0018 | NA |
| BMI | Yengo | Publication supplement | rs2479958 | A | G | 0.4925 | 0.0154 | 0.0018 | NA |
| BMI | Yengo | Publication supplement | rs248139 | A | G | 0.1933 | 0.0133 | 0.0022 | NA |
| BMI | Yengo | Publication supplement | rs2481665 | T | C | 0.5592 | 0.0161 | 0.0016 | NA |
| BMI | Yengo | Publication supplement | rs2489676 | T | G | 0.4507 | -0.01 | 0.0017 | NA |
| BMI | Yengo | Publication supplement | rs249292 | T | C | 0.6895 | 0.012 | 0.0019 | NA |
| BMI | Yengo | Publication supplement | rs2516739 | A | G | 0.2171 | -0.0159 | 0.0021 | NA |
| BMI | Yengo | Publication supplement | rs2527366 | A | G | 0.6589 | -0.0107 | 0.0018 | NA |
| BMI | Yengo | Publication supplement | rs252749 | A | G | 0.2422 | -0.0115 | 0.002 | NA |
| BMI | Yengo | Publication supplement | rs2543132 | C | G | 0.8134 | 0.0146 | 0.0022 | NA |
| BMI | Yengo | Publication supplement | rs254800 | T | G | 0.5208 | 0.0098 | 0.0017 | NA |
| BMI | Yengo | Publication supplement | rs2600226 | T | C | 0.6697 | -0.0116 | 0.0019 | NA |
| BMI | Yengo | Publication supplement | rs2605603 | A | G | 0.4887 | -0.0103 | 0.0016 | NA |
| BMI | Yengo | Publication supplement | rs2616192 | T | G | 0.6775 | 0.0125 | 0.0019 | NA |
| BMI | Yengo | Publication supplement | rs2619976 | T | C | 0.4132 | 0.0104 | 0.0018 | NA |
| BMI | Yengo | Publication supplement | rs263041 | A | G | 0.3669 | 0.0124 | 0.0018 | NA |
| BMI | Yengo | Publication supplement | rs2682406 | A | T | 0.4174 | -0.0132 | 0.0018 | NA |
| BMI | Yengo | Publication supplement | rs2694047 | A | G | 0.253 | -0.0188 | 0.002 | NA |
| BMI | Yengo | Publication supplement | rs2717926 | T | C | 0.6624 | 0.0097 | 0.0017 | NA |
| BMI | Yengo | Publication supplement | rs2718786 | A | G | 0.3808 | 0.0112 | 0.0018 | NA |
| BMI | Yengo | Publication supplement | rs2731277 | T | C | 0.7464 | -0.0135 | 0.002 | NA |
| BMI | Yengo | Publication supplement | rs2732275 | A | G | 0.616 | 0.0104 | 0.0018 | NA |
| BMI | Yengo | Publication supplement | rs2733287 | A | C | 0.5171 | -0.0157 | 0.0017 | NA |
| BMI | Yengo | Publication supplement | rs273504 | A | G | 0.5734 | -0.0153 | 0.0018 | NA |
| BMI | Yengo | Publication supplement | rs273697 | A | G | 0.4779 | -0.0098 | 0.0017 | NA |
| **Trait** | **GWAS author** | **Source** | **SNP** | **Effect Allele** | **Other Allele** | **EAF** | **beta** | **p** | **MR-Base/EBI Study ID** |
| BMI | Yengo | Publication supplement | rs2744974 | T | C | 0.338 | 0.0249 | 0.0018 | NA |
| BMI | Yengo | Publication supplement | rs2815324 | T | C | 0.7835 | -0.0129 | 0.002 | NA |
| BMI | Yengo | Publication supplement | rs2820311 | A | G | 0.6631 | -0.0235 | 0.0018 | NA |
| BMI | Yengo | Publication supplement | rs2832283 | A | G | 0.2208 | 0.0115 | 0.002 | NA |
| BMI | Yengo | Publication supplement | rs2836961 | A | C | 0.6165 | -0.0102 | 0.0017 | NA |
| BMI | Yengo | Publication supplement | rs2837398 | A | C | 0.5958 | -0.0114 | 0.0018 | NA |
| BMI | Yengo | Publication supplement | rs284227 | T | C | 0.7448 | -0.0147 | 0.0019 | NA |
| BMI | Yengo | Publication supplement | rs284262 | T | C | 0.4533 | 0.0099 | 0.0018 | NA |
| BMI | Yengo | Publication supplement | rs2850969 | T | C | 0.8573 | -0.0165 | 0.0024 | NA |
| BMI | Yengo | Publication supplement | rs2866816 | T | C | 0.7393 | 0.0129 | 0.002 | NA |
| BMI | Yengo | Publication supplement | rs2874800 | A | G | 0.3213 | -0.0118 | 0.0019 | NA |
| BMI | Yengo | Publication supplement | rs2875762 | C | G | 0.2473 | 0.0139 | 0.002 | NA |
| BMI | Yengo | Publication supplement | rs2890652 | T | C | 0.8241 | -0.017 | 0.0023 | NA |
| BMI | Yengo | Publication supplement | rs2907948 | A | G | 0.2427 | -0.0141 | 0.0019 | NA |
| BMI | Yengo | Publication supplement | rs2923774 | A | G | 0.6589 | 0.0105 | 0.0018 | NA |
| BMI | Yengo | Publication supplement | rs2931434 | T | C | 0.3168 | -0.0104 | 0.0018 | NA |
| BMI | Yengo | Publication supplement | rs294704 | T | G | 0.7239 | -0.0113 | 0.0019 | NA |
| BMI | Yengo | Publication supplement | rs2959592 | T | C | 0.8045 | 0.0118 | 0.0021 | NA |
| BMI | Yengo | Publication supplement | rs298563 | T | C | 0.1807 | 0.0127 | 0.0023 | NA |
| BMI | Yengo | Publication supplement | rs29941 | A | G | 0.3135 | -0.0152 | 0.0018 | NA |
| BMI | Yengo | Publication supplement | rs3005710 | A | G | 0.2117 | 0.0117 | 0.0021 | NA |
| BMI | Yengo | Publication supplement | rs3007105 | T | C | 0.4697 | 0.0142 | 0.0017 | NA |
| BMI | Yengo | Publication supplement | rs3209570 | A | G | 0.3748 | -0.0143 | 0.0018 | NA |
| BMI | Yengo | Publication supplement | rs323742 | A | T | 0.9721 | -0.041 | 0.0056 | NA |
| BMI | Yengo | Publication supplement | rs326889 | T | C | 0.3928 | -0.0129 | 0.0018 | NA |
| BMI | Yengo | Publication supplement | rs329122 | A | G | 0.4226 | -0.0125 | 0.0017 | NA |
| BMI | Yengo | Publication supplement | rs329651 | T | G | 0.8055 | 0.0164 | 0.0021 | NA |
| BMI | Yengo | Publication supplement | rs331949 | T | C | 0.6266 | -0.011 | 0.0017 | NA |
| BMI | Yengo | Publication supplement | rs33436 | A | G | 0.3706 | -0.0125 | 0.0018 | NA |
| **Trait** | **GWAS author** | **Source** | **SNP** | **Effect Allele** | **Other Allele** | **EAF** | **beta** | **p** | **MR-Base/EBI Study ID** |
| BMI | Yengo | Publication supplement | rs33485 | T | C | 0.7309 | -0.0158 | 0.002 | NA |
| BMI | Yengo | Publication supplement | rs349088 | A | C | 0.4976 | -0.0128 | 0.0017 | NA |
| BMI | Yengo | Publication supplement | rs355777 | C | G | 0.4106 | 0.0153 | 0.0017 | NA |
| BMI | Yengo | Publication supplement | rs3736485 | A | G | 0.4557 | 0.0134 | 0.0016 | NA |
| BMI | Yengo | Publication supplement | rs3764835 | A | G | 0.1528 | -0.0141 | 0.0024 | NA |
| BMI | Yengo | Publication supplement | rs3766430 | T | C | 0.5591 | -0.0113 | 0.0016 | NA |
| BMI | Yengo | Publication supplement | rs3769948 | A | G | 0.7534 | 0.0125 | 0.002 | NA |
| BMI | Yengo | Publication supplement | rs3781099 | T | C | 0.082 | 0.021 | 0.0031 | NA |
| BMI | Yengo | Publication supplement | rs3800229 | T | G | 0.7123 | 0.0175 | 0.0018 | NA |
| BMI | Yengo | Publication supplement | rs3803286 | A | G | 0.3431 | 0.0181 | 0.0018 | NA |
| BMI | Yengo | Publication supplement | rs3806116 | T | G | 0.3832 | 0.0104 | 0.0017 | NA |
| BMI | Yengo | Publication supplement | rs3807566 | T | G | 0.443 | -0.0127 | 0.0017 | NA |
| BMI | Yengo | Publication supplement | rs3807875 | A | G | 0.6287 | -0.0094 | 0.0017 | NA |
| BMI | Yengo | Publication supplement | rs3810291 | A | G | 0.6703 | 0.0274 | 0.0018 | NA |
| BMI | Yengo | Publication supplement | rs3811514 | T | C | 0.5123 | 0.0121 | 0.0017 | NA |
| BMI | Yengo | Publication supplement | rs3813680 | A | G | 0.8438 | 0.0144 | 0.0024 | NA |
| BMI | Yengo | Publication supplement | rs3814883 | T | C | 0.4764 | 0.0232 | 0.0017 | NA |
| BMI | Yengo | Publication supplement | rs3819811 | A | G | 0.3172 | -0.0103 | 0.0019 | NA |
| BMI | Yengo | Publication supplement | rs3826705 | T | C | 0.8794 | -0.0157 | 0.0027 | NA |
| BMI | Yengo | Publication supplement | rs3829849 | T | C | 0.3589 | 0.0098 | 0.0017 | NA |
| BMI | Yengo | Publication supplement | rs3844598 | A | G | 0.479 | -0.0095 | 0.0017 | NA |
| BMI | Yengo | Publication supplement | rs3845802 | T | G | 0.514 | -0.0115 | 0.0016 | NA |
| BMI | Yengo | Publication supplement | rs3849570 | A | C | 0.3414 | 0.0132 | 0.0017 | NA |
| BMI | Yengo | Publication supplement | rs3851083 | A | G | 0.4325 | -0.0102 | 0.0016 | NA |
| BMI | Yengo | Publication supplement | rs3852012 | A | G | 0.6876 | -0.0131 | 0.0018 | NA |
| BMI | Yengo | Publication supplement | rs3887080 | A | G | 0.1214 | 0.0181 | 0.0026 | NA |
| BMI | Yengo | Publication supplement | rs3902951 | T | G | 0.7545 | -0.0134 | 0.002 | NA |
| BMI | Yengo | Publication supplement | rs3926926 | A | G | 0.1873 | -0.0128 | 0.0022 | NA |
| BMI | Yengo | Publication supplement | rs3930349 | A | C | 0.2196 | -0.0144 | 0.0021 | NA |
| **Trait** | **GWAS author** | **Source** | **SNP** | **Effect Allele** | **Other Allele** | **EAF** | **beta** | **p** | **MR-Base/EBI Study ID** |
| BMI | Yengo | Publication supplement | rs3957285 | A | G | 0.5261 | 0.0144 | 0.0018 | NA |
| BMI | Yengo | Publication supplement | rs4012234 | T | G | 0.4076 | -0.0141 | 0.0018 | NA |
| BMI | Yengo | Publication supplement | rs40245 | A | T | 0.3591 | 0.011 | 0.0018 | NA |
| BMI | Yengo | Publication supplement | rs4072917 | A | G | 0.4694 | 0.0115 | 0.0018 | NA |
| BMI | Yengo | Publication supplement | rs4077093 | T | G | 0.217 | 0.0128 | 0.0022 | NA |
| BMI | Yengo | Publication supplement | rs4082793 | T | C | 0.421 | -0.0122 | 0.0018 | NA |
| BMI | Yengo | Publication supplement | rs4148155 | A | G | 0.8873 | 0.0188 | 0.0026 | NA |
| BMI | Yengo | Publication supplement | rs4148866 | T | C | 0.4068 | 0.0098 | 0.0018 | NA |
| BMI | Yengo | Publication supplement | rs420158 | T | C | 0.734 | -0.0119 | 0.0019 | NA |
| BMI | Yengo | Publication supplement | rs4237643 | T | G | 0.3062 | 0.0223 | 0.0019 | NA |
| BMI | Yengo | Publication supplement | rs424539 | C | G | 0.6113 | -0.011 | 0.0018 | NA |
| BMI | Yengo | Publication supplement | rs427943 | A | C | 0.4331 | -0.017 | 0.0017 | NA |
| BMI | Yengo | Publication supplement | rs429343 | A | G | 0.4187 | 0.015 | 0.0017 | NA |
| BMI | Yengo | Publication supplement | rs4307239 | A | G | 0.5422 | -0.0115 | 0.0017 | NA |
| BMI | Yengo | Publication supplement | rs4372836 | T | C | 0.3034 | 0.0142 | 0.0018 | NA |
| BMI | Yengo | Publication supplement | rs4430672 | T | C | 0.1996 | 0.0127 | 0.0022 | NA |
| BMI | Yengo | Publication supplement | rs4482463 | A | C | 0.9213 | -0.0331 | 0.0033 | NA |
| BMI | Yengo | Publication supplement | rs4483850 | A | T | 0.509 | 0.0162 | 0.0017 | NA |
| BMI | Yengo | Publication supplement | rs4516268 | A | C | 0.1925 | -0.0217 | 0.0021 | NA |
| BMI | Yengo | Publication supplement | rs4518345 | A | G | 0.2842 | -0.0117 | 0.0019 | NA |
| BMI | Yengo | Publication supplement | rs4521182 | T | C | 0.31 | 0.0104 | 0.0019 | NA |
| BMI | Yengo | Publication supplement | rs4523552 | T | C | 0.2229 | 0.0137 | 0.0021 | NA |
| BMI | Yengo | Publication supplement | rs4523610 | T | C | 0.771 | -0.0123 | 0.002 | NA |
| BMI | Yengo | Publication supplement | rs4538727 | A | G | 0.401 | -0.0099 | 0.0018 | NA |
| BMI | Yengo | Publication supplement | rs455527 | T | C | 0.9441 | 0.0199 | 0.0035 | NA |
| BMI | Yengo | Publication supplement | rs4556997 | A | C | 0.1349 | 0.0197 | 0.0024 | NA |
| BMI | Yengo | Publication supplement | rs459552 | A | T | 0.7691 | -0.0126 | 0.002 | NA |
| BMI | Yengo | Publication supplement | rs460799 | A | G | 0.2681 | -0.0118 | 0.0019 | NA |
| BMI | Yengo | Publication supplement | rs4639527 | A | G | 0.6988 | -0.0172 | 0.0019 | NA |
| **Trait** | **GWAS author** | **Source** | **SNP** | **Effect Allele** | **Other Allele** | **EAF** | **beta** | **p** | **MR-Base/EBI Study ID** |
| BMI | Yengo | Publication supplement | rs4653017 | T | C | 0.6818 | 0.0122 | 0.0018 | NA |
| BMI | Yengo | Publication supplement | rs4670627 | T | C | 0.5629 | 0.0105 | 0.0017 | NA |
| BMI | Yengo | Publication supplement | rs4671328 | T | G | 0.4467 | 0.0219 | 0.0017 | NA |
| BMI | Yengo | Publication supplement | rs4673553 | T | G | 0.5438 | -0.0142 | 0.0017 | NA |
| BMI | Yengo | Publication supplement | rs4676084 | A | G | 0.4101 | 0.0098 | 0.0017 | NA |
| BMI | Yengo | Publication supplement | rs4677812 | A | C | 0.2806 | -0.0136 | 0.0019 | NA |
| BMI | Yengo | Publication supplement | rs4682718 | A | G | 0.82 | -0.0154 | 0.0023 | NA |
| BMI | Yengo | Publication supplement | rs4700646 | A | G | 0.474 | 0.0109 | 0.0017 | NA |
| BMI | Yengo | Publication supplement | rs4704513 | C | G | 0.8189 | -0.0125 | 0.0022 | NA |
| BMI | Yengo | Publication supplement | rs4711986 | A | G | 0.3541 | 0.0143 | 0.0018 | NA |
| BMI | Yengo | Publication supplement | rs4717623 | T | C | 0.2551 | -0.0135 | 0.002 | NA |
| BMI | Yengo | Publication supplement | rs4718966 | T | C | 0.4183 | 0.0127 | 0.0018 | NA |
| BMI | Yengo | Publication supplement | rs4722398 | T | C | 0.1336 | 0.0158 | 0.0025 | NA |
| BMI | Yengo | Publication supplement | rs4722672 | T | C | 0.8151 | -0.0151 | 0.0021 | NA |
| BMI | Yengo | Publication supplement | rs472611 | A | G | 0.6616 | -0.0101 | 0.0018 | NA |
| BMI | Yengo | Publication supplement | rs4740383 | A | G | 0.416 | 0.0126 | 0.0018 | NA |
| BMI | Yengo | Publication supplement | rs4740619 | T | C | 0.5479 | 0.0186 | 0.0016 | NA |
| BMI | Yengo | Publication supplement | rs4745794 | A | G | 0.4843 | -0.0114 | 0.0017 | NA |
| BMI | Yengo | Publication supplement | rs4757144 | A | G | 0.5878 | 0.0169 | 0.0018 | NA |
| BMI | Yengo | Publication supplement | rs4757638 | A | G | 0.5896 | 0.0101 | 0.0018 | NA |
| BMI | Yengo | Publication supplement | rs4759075 | T | C | 0.5986 | 0.0112 | 0.0017 | NA |
| BMI | Yengo | Publication supplement | rs4783241 | C | G | 0.4938 | -0.0108 | 0.0017 | NA |
| BMI | Yengo | Publication supplement | rs4796243 | A | G | 0.3029 | -0.0124 | 0.0019 | NA |
| BMI | Yengo | Publication supplement | rs4802778 | A | G | 0.5745 | -0.0116 | 0.0018 | NA |
| BMI | Yengo | Publication supplement | rs4814512 | A | C | 0.782 | 0.0133 | 0.0021 | NA |
| BMI | Yengo | Publication supplement | rs4820408 | T | G | 0.408 | 0.0151 | 0.0017 | NA |
| BMI | Yengo | Publication supplement | rs4835778 | A | G | 0.8203 | -0.0127 | 0.0023 | NA |
| BMI | Yengo | Publication supplement | rs483752 | T | C | 0.7415 | 0.0121 | 0.0022 | NA |
| BMI | Yengo | Publication supplement | rs4856794 | T | G | 0.6514 | -0.011 | 0.0018 | NA |
| **Trait** | **GWAS author** | **Source** | **SNP** | **Effect Allele** | **Other Allele** | **EAF** | **beta** | **p** | **MR-Base/EBI Study ID** |
| BMI | Yengo | Publication supplement | rs4858193 | T | C | 0.7221 | 0.0129 | 0.0019 | NA |
| BMI | Yengo | Publication supplement | rs4860782 | A | C | 0.1902 | -0.0122 | 0.0022 | NA |
| BMI | Yengo | Publication supplement | rs486359 | C | G | 0.4853 | 0.0112 | 0.0017 | NA |
| BMI | Yengo | Publication supplement | rs4864201 | T | C | 0.3531 | 0.0141 | 0.0017 | NA |
| BMI | Yengo | Publication supplement | rs4865796 | A | G | 0.6908 | -0.0103 | 0.0018 | NA |
| BMI | Yengo | Publication supplement | rs4877313 | A | T | 0.7817 | 0.0129 | 0.0021 | NA |
| BMI | Yengo | Publication supplement | rs4880341 | T | C | 0.5606 | -0.0118 | 0.0017 | NA |
| BMI | Yengo | Publication supplement | rs4886869 | A | G | 0.3952 | -0.01 | 0.0018 | NA |
| BMI | Yengo | Publication supplement | rs4889606 | A | G | 0.6199 | 0.0202 | 0.0017 | NA |
| BMI | Yengo | Publication supplement | rs4906908 | T | G | 0.4747 | -0.0103 | 0.0017 | NA |
| BMI | Yengo | Publication supplement | rs4911442 | A | G | 0.8728 | 0.015 | 0.0024 | NA |
| BMI | Yengo | Publication supplement | rs4912637 | A | G | 0.3461 | -0.0103 | 0.0018 | NA |
| BMI | Yengo | Publication supplement | rs4916229 | C | G | 0.9073 | -0.0183 | 0.0029 | NA |
| BMI | Yengo | Publication supplement | rs491711 | A | C | 0.684 | 0.0115 | 0.0019 | NA |
| BMI | Yengo | Publication supplement | rs4929923 | T | C | 0.3624 | -0.0181 | 0.0017 | NA |
| BMI | Yengo | Publication supplement | rs4936175 | T | C | 0.5555 | -0.0122 | 0.0017 | NA |
| BMI | Yengo | Publication supplement | rs4936671 | C | G | 0.3672 | 0.0105 | 0.0018 | NA |
| BMI | Yengo | Publication supplement | rs4953577 | T | C | 0.5525 | 0.0099 | 0.0017 | NA |
| BMI | Yengo | Publication supplement | rs4954638 | A | C | 0.7508 | 0.0118 | 0.002 | NA |
| BMI | Yengo | Publication supplement | rs4963120 | T | C | 0.5654 | 0.013 | 0.0018 | NA |
| BMI | Yengo | Publication supplement | rs4969387 | C | G | 0.7463 | -0.0145 | 0.002 | NA |
| BMI | Yengo | Publication supplement | rs498240 | A | G | 0.0656 | -0.0267 | 0.0033 | NA |
| BMI | Yengo | Publication supplement | rs4985155 | A | G | 0.6631 | 0.012 | 0.0017 | NA |
| BMI | Yengo | Publication supplement | rs4986044 | T | C | 0.4687 | -0.0164 | 0.0016 | NA |
| BMI | Yengo | Publication supplement | rs4989244 | A | G | 0.4112 | -0.0113 | 0.0017 | NA |
| BMI | Yengo | Publication supplement | rs506338 | T | C | 0.2984 | -0.0134 | 0.0018 | NA |
| BMI | Yengo | Publication supplement | rs535533 | T | C | 0.594 | -0.0104 | 0.0018 | NA |
| BMI | Yengo | Publication supplement | rs536445 | T | C | 0.5293 | 0.014 | 0.0017 | NA |
| BMI | Yengo | Publication supplement | rs538579 | C | G | 0.3228 | 0.0137 | 0.0019 | NA |
| **Trait** | **GWAS author** | **Source** | **SNP** | **Effect Allele** | **Other Allele** | **EAF** | **beta** | **p** | **MR-Base/EBI Study ID** |
| BMI | Yengo | Publication supplement | rs543874 | A | G | 0.8048 | -0.0475 | 0.002 | NA |
| BMI | Yengo | Publication supplement | rs573455 | A | G | 0.453 | -0.0093 | 0.0016 | NA |
| BMI | Yengo | Publication supplement | rs5742914 | T | C | 0.1355 | 0.0175 | 0.0027 | NA |
| BMI | Yengo | Publication supplement | rs5751239 | T | C | 0.5171 | 0.0109 | 0.0017 | NA |
| BMI | Yengo | Publication supplement | rs577525 | T | C | 0.4324 | -0.0166 | 0.0017 | NA |
| BMI | Yengo | Publication supplement | rs591088 | T | C | 0.5589 | -0.0097 | 0.0017 | NA |
| BMI | Yengo | Publication supplement | rs592483 | T | C | 0.5716 | -0.0147 | 0.0017 | NA |
| BMI | Yengo | Publication supplement | rs6011457 | A | T | 0.4975 | -0.0116 | 0.0017 | NA |
| BMI | Yengo | Publication supplement | rs6014523 | T | C | 0.8032 | 0.0149 | 0.0022 | NA |
| BMI | Yengo | Publication supplement | rs6019482 | T | C | 0.1654 | -0.0178 | 0.0023 | NA |
| BMI | Yengo | Publication supplement | rs6023633 | A | G | 0.763 | -0.0138 | 0.0021 | NA |
| BMI | Yengo | Publication supplement | rs6047046 | A | G | 0.5139 | 0.0098 | 0.0017 | NA |
| BMI | Yengo | Publication supplement | rs6050446 | A | G | 0.03 | -0.0343 | 0.0047 | NA |
| BMI | Yengo | Publication supplement | rs6056413 | A | T | 0.1711 | 0.0127 | 0.0022 | NA |
| BMI | Yengo | Publication supplement | rs610634 | T | C | 0.8179 | 0.0138 | 0.0023 | NA |
| BMI | Yengo | Publication supplement | rs6121381 | A | T | 0.8469 | -0.0147 | 0.0024 | NA |
| BMI | Yengo | Publication supplement | rs6138482 | T | C | 0.1974 | 0.0147 | 0.002 | NA |
| BMI | Yengo | Publication supplement | rs621042 | A | C | 0.451 | -0.0107 | 0.0017 | NA |
| BMI | Yengo | Publication supplement | rs6235 | C | G | 0.7298 | -0.0175 | 0.0019 | NA |
| BMI | Yengo | Publication supplement | rs6265 | T | C | 0.1951 | -0.0412 | 0.0021 | NA |
| BMI | Yengo | Publication supplement | rs6419734 | T | C | 0.1521 | 0.0174 | 0.0025 | NA |
| BMI | Yengo | Publication supplement | rs6433243 | T | C | 0.3465 | 0.0102 | 0.0018 | NA |
| BMI | Yengo | Publication supplement | rs6445538 | T | C | 0.7646 | -0.0149 | 0.002 | NA |
| BMI | Yengo | Publication supplement | rs6448587 | A | C | 0.8109 | 0.0167 | 0.0023 | NA |
| BMI | Yengo | Publication supplement | rs6449532 | T | C | 0.3545 | -0.0127 | 0.0018 | NA |
| BMI | Yengo | Publication supplement | rs6461115 | A | G | 0.7715 | 0.0144 | 0.0019 | NA |
| BMI | Yengo | Publication supplement | rs6471941 | A | G | 0.1684 | 0.0156 | 0.0021 | NA |
| BMI | Yengo | Publication supplement | rs6477694 | T | C | 0.6441 | -0.0123 | 0.0017 | NA |
| BMI | Yengo | Publication supplement | rs6482729 | T | C | 0.6615 | -0.0102 | 0.0019 | NA |
| **Trait** | **GWAS author** | **Source** | **SNP** | **Effect Allele** | **Other Allele** | **EAF** | **beta** | **p** | **MR-Base/EBI Study ID** |
| BMI | Yengo | Publication supplement | rs6497676 | A | G | 0.6417 | -0.0098 | 0.0018 | NA |
| BMI | Yengo | Publication supplement | rs6512302 | C | G | 0.7511 | 0.0142 | 0.002 | NA |
| BMI | Yengo | Publication supplement | rs653264 | A | G | 0.5306 | -0.0099 | 0.0017 | NA |
| BMI | Yengo | Publication supplement | rs6545709 | A | G | 0.099 | -0.0203 | 0.003 | NA |
| BMI | Yengo | Publication supplement | rs6545714 | A | G | 0.6139 | -0.0191 | 0.0017 | NA |
| BMI | Yengo | Publication supplement | rs6548221 | A | G | 0.2246 | 0.0151 | 0.002 | NA |
| BMI | Yengo | Publication supplement | rs6548834 | A | G | 0.3622 | 0.0136 | 0.0018 | NA |
| BMI | Yengo | Publication supplement | rs6556301 | T | G | 0.3596 | -0.0111 | 0.0018 | NA |
| BMI | Yengo | Publication supplement | rs6561710 | A | G | 0.5831 | -0.0105 | 0.0018 | NA |
| BMI | Yengo | Publication supplement | rs6561766 | A | G | 0.119 | 0.0168 | 0.0028 | NA |
| BMI | Yengo | Publication supplement | rs6564360 | A | G | 0.8095 | -0.0135 | 0.0022 | NA |
| BMI | Yengo | Publication supplement | rs657452 | A | G | 0.3784 | 0.0188 | 0.0017 | NA |
| BMI | Yengo | Publication supplement | rs6574695 | C | G | 0.8659 | -0.0146 | 0.0026 | NA |
| BMI | Yengo | Publication supplement | rs6587552 | A | G | 0.2409 | 0.0173 | 0.002 | NA |
| BMI | Yengo | Publication supplement | rs6591407 | A | C | 0.1861 | -0.0118 | 0.0021 | NA |
| BMI | Yengo | Publication supplement | rs6594967 | T | C | 0.6726 | 0.0109 | 0.0018 | NA |
| BMI | Yengo | Publication supplement | rs6595205 | C | G | 0.4695 | 0.0114 | 0.0016 | NA |
| BMI | Yengo | Publication supplement | rs6606686 | C | G | 0.6903 | -0.0136 | 0.0018 | NA |
| BMI | Yengo | Publication supplement | rs663129 | A | G | 0.2301 | 0.0545 | 0.0019 | NA |
| BMI | Yengo | Publication supplement | rs6661316 | T | C | 0.5765 | 0.0106 | 0.0016 | NA |
| BMI | Yengo | Publication supplement | rs6662747 | A | G | 0.5311 | -0.0109 | 0.0017 | NA |
| BMI | Yengo | Publication supplement | rs6676084 | T | C | 0.3091 | -0.0122 | 0.0018 | NA |
| BMI | Yengo | Publication supplement | rs6690764 | A | G | 0.7916 | -0.0154 | 0.0022 | NA |
| BMI | Yengo | Publication supplement | rs6691857 | T | C | 0.0315 | -0.0347 | 0.0051 | NA |
| BMI | Yengo | Publication supplement | rs6692586 | A | G | 0.168 | 0.0192 | 0.0023 | NA |
| BMI | Yengo | Publication supplement | rs6707445 | A | G | 0.4543 | 0.0128 | 0.0017 | NA |
| BMI | Yengo | Publication supplement | rs6710871 | A | G | 0.1418 | 0.0179 | 0.0024 | NA |
| BMI | Yengo | Publication supplement | rs6712 | C | G | 0.1368 | 0.0138 | 0.0025 | NA |
| BMI | Yengo | Publication supplement | rs6713781 | C | G | 0.4136 | -0.0123 | 0.0018 | NA |
| **Trait** | **GWAS author** | **Source** | **SNP** | **Effect Allele** | **Other Allele** | **EAF** | **beta** | **p** | **MR-Base/EBI Study ID** |
| BMI | Yengo | Publication supplement | rs6738445 | T | C | 0.2836 | -0.0133 | 0.0018 | NA |
| BMI | Yengo | Publication supplement | rs6764533 | A | G | 0.359 | 0.0116 | 0.0018 | NA |
| BMI | Yengo | Publication supplement | rs676749 | A | T | 0.502 | -0.0104 | 0.0017 | NA |
| BMI | Yengo | Publication supplement | rs6767619 | C | G | 0.345 | 0.0119 | 0.0018 | NA |
| BMI | Yengo | Publication supplement | rs6777784 | T | G | 0.6221 | 0.0105 | 0.0018 | NA |
| BMI | Yengo | Publication supplement | rs6781254 | T | C | 0.3034 | 0.0112 | 0.0018 | NA |
| BMI | Yengo | Publication supplement | rs6804181 | A | T | 0.8213 | 0.0153 | 0.0023 | NA |
| BMI | Yengo | Publication supplement | rs6804842 | A | G | 0.428 | -0.0156 | 0.0017 | NA |
| BMI | Yengo | Publication supplement | rs6807940 | C | G | 0.4018 | -0.0102 | 0.0018 | NA |
| BMI | Yengo | Publication supplement | rs6819344 | A | C | 0.4005 | 0.0103 | 0.0017 | NA |
| BMI | Yengo | Publication supplement | rs6827083 | A | G | 0.5691 | -0.0097 | 0.0016 | NA |
| BMI | Yengo | Publication supplement | rs6841761 | T | G | 0.5252 | -0.0131 | 0.0016 | NA |
| BMI | Yengo | Publication supplement | rs6849518 | T | C | 0.1263 | 0.0173 | 0.0026 | NA |
| BMI | Yengo | Publication supplement | rs6850639 | T | C | 0.2084 | 0.0124 | 0.0021 | NA |
| BMI | Yengo | Publication supplement | rs6852276 | A | G | 0.5982 | -0.0108 | 0.0017 | NA |
| BMI | Yengo | Publication supplement | rs6864049 | A | G | 0.4606 | -0.0125 | 0.0017 | NA |
| BMI | Yengo | Publication supplement | rs6870983 | T | C | 0.2177 | -0.0204 | 0.002 | NA |
| BMI | Yengo | Publication supplement | rs6879326 | T | C | 0.4953 | -0.0098 | 0.0017 | NA |
| BMI | Yengo | Publication supplement | rs6898812 | T | G | 0.4411 | -0.0104 | 0.0018 | NA |
| BMI | Yengo | Publication supplement | rs6901756 | T | C | 0.8779 | 0.0146 | 0.0025 | NA |
| BMI | Yengo | Publication supplement | rs6921533 | T | C | 0.2882 | 0.0118 | 0.0019 | NA |
| BMI | Yengo | Publication supplement | rs6930066 | T | C | 0.3433 | 0.0104 | 0.0018 | NA |
| BMI | Yengo | Publication supplement | rs6968554 | A | G | 0.3602 | -0.01 | 0.0017 | NA |
| BMI | Yengo | Publication supplement | rs6985109 | A | G | 0.5338 | -0.0177 | 0.0017 | NA |
| BMI | Yengo | Publication supplement | rs7006629 | T | C | 0.5258 | 0.0109 | 0.0017 | NA |
| BMI | Yengo | Publication supplement | rs7009017 | C | G | 0.1203 | 0.0148 | 0.0027 | NA |
| BMI | Yengo | Publication supplement | rs7024334 | T | G | 0.2258 | 0.0138 | 0.002 | NA |
| BMI | Yengo | Publication supplement | rs7025938 | C | G | 0.6813 | -0.0166 | 0.0019 | NA |
| BMI | Yengo | Publication supplement | rs702820 | T | C | 0.8844 | -0.017 | 0.0027 | NA |
| **Trait** | **GWAS author** | **Source** | **SNP** | **Effect Allele** | **Other Allele** | **EAF** | **beta** | **p** | **MR-Base/EBI Study ID** |
| BMI | Yengo | Publication supplement | rs7083450 | T | C | 0.8388 | 0.0159 | 0.0023 | NA |
| BMI | Yengo | Publication supplement | rs7102454 | T | C | 0.6565 | -0.0158 | 0.0018 | NA |
| BMI | Yengo | Publication supplement | rs7117238 | A | G | 0.168 | -0.0131 | 0.0022 | NA |
| BMI | Yengo | Publication supplement | rs7123876 | T | C | 0.754 | -0.0119 | 0.0019 | NA |
| BMI | Yengo | Publication supplement | rs7124681 | A | C | 0.4133 | 0.0263 | 0.0016 | NA |
| BMI | Yengo | Publication supplement | rs7133378 | A | G | 0.3269 | 0.0125 | 0.0018 | NA |
| BMI | Yengo | Publication supplement | rs7134628 | A | G | 0.1027 | 0.0172 | 0.0028 | NA |
| BMI | Yengo | Publication supplement | rs7138803 | A | G | 0.3772 | 0.03 | 0.0017 | NA |
| BMI | Yengo | Publication supplement | rs7144011 | T | G | 0.2136 | 0.0282 | 0.002 | NA |
| BMI | Yengo | Publication supplement | rs7147503 | T | C | 0.3697 | -0.0119 | 0.0018 | NA |
| BMI | Yengo | Publication supplement | rs7164727 | T | C | 0.681 | 0.0182 | 0.0017 | NA |
| BMI | Yengo | Publication supplement | rs7172627 | A | G | 0.5281 | -0.0117 | 0.0017 | NA |
| BMI | Yengo | Publication supplement | rs7181498 | T | C | 0.3691 | 0.0163 | 0.0018 | NA |
| BMI | Yengo | Publication supplement | rs7181610 | A | T | 0.8585 | 0.0145 | 0.0025 | NA |
| BMI | Yengo | Publication supplement | rs7186893 | T | G | 0.2686 | -0.0141 | 0.002 | NA |
| BMI | Yengo | Publication supplement | rs7195386 | T | C | 0.5014 | 0.0133 | 0.0017 | NA |
| BMI | Yengo | Publication supplement | rs719802 | T | C | 0.3803 | 0.0101 | 0.0018 | NA |
| BMI | Yengo | Publication supplement | rs7206395 | A | G | 0.1984 | 0.0123 | 0.0022 | NA |
| BMI | Yengo | Publication supplement | rs7206608 | C | G | 0.6854 | -0.0132 | 0.0019 | NA |
| BMI | Yengo | Publication supplement | rs7209235 | A | G | 0.695 | -0.0111 | 0.0019 | NA |
| BMI | Yengo | Publication supplement | rs7217226 | T | G | 0.6404 | -0.013 | 0.0017 | NA |
| BMI | Yengo | Publication supplement | rs7220138 | C | G | 0.696 | -0.0123 | 0.0019 | NA |
| BMI | Yengo | Publication supplement | rs7235205 | A | G | 0.7343 | -0.0141 | 0.0019 | NA |
| BMI | Yengo | Publication supplement | rs7235563 | C | G | 0.642 | 0.0098 | 0.0018 | NA |
| BMI | Yengo | Publication supplement | rs7239114 | A | G | 0.5403 | 0.0124 | 0.0017 | NA |
| BMI | Yengo | Publication supplement | rs7243357 | T | G | 0.8272 | 0.0194 | 0.0021 | NA |
| BMI | Yengo | Publication supplement | rs7249143 | T | G | 0.3082 | 0.0126 | 0.0019 | NA |
| BMI | Yengo | Publication supplement | rs7254892 | A | G | 0.0362 | 0.0281 | 0.0048 | NA |
| BMI | Yengo | Publication supplement | rs7313220 | A | G | 0.4861 | 0.0122 | 0.0017 | NA |
| **Trait** | **GWAS author** | **Source** | **SNP** | **Effect Allele** | **Other Allele** | **EAF** | **beta** | **p** | **MR-Base/EBI Study ID** |
| BMI | Yengo | Publication supplement | rs731834 | A | C | 0.5197 | 0.0107 | 0.0018 | NA |
| BMI | Yengo | Publication supplement | rs7332115 | T | G | 0.6266 | 0.0159 | 0.0017 | NA |
| BMI | Yengo | Publication supplement | rs7334078 | T | C | 0.7118 | 0.0121 | 0.0019 | NA |
| BMI | Yengo | Publication supplement | rs733594 | T | C | 0.718 | 0.0138 | 0.0018 | NA |
| BMI | Yengo | Publication supplement | rs740157 | A | G | 0.4322 | 0.0116 | 0.0016 | NA |
| BMI | Yengo | Publication supplement | rs7421089 | T | C | 0.2888 | 0.0135 | 0.0019 | NA |
| BMI | Yengo | Publication supplement | rs7488867 | T | C | 0.2639 | -0.0204 | 0.002 | NA |
| BMI | Yengo | Publication supplement | rs7498665 | A | G | 0.5962 | -0.0271 | 0.0017 | NA |
| BMI | Yengo | Publication supplement | rs7512146 | T | G | 0.5084 | -0.0094 | 0.0017 | NA |
| BMI | Yengo | Publication supplement | rs7519259 | A | G | 0.5356 | 0.0125 | 0.0017 | NA |
| BMI | Yengo | Publication supplement | rs7534091 | A | G | 0.7493 | -0.0113 | 0.0019 | NA |
| BMI | Yengo | Publication supplement | rs7535528 | A | G | 0.3741 | -0.0152 | 0.0018 | NA |
| BMI | Yengo | Publication supplement | rs7536433 | T | C | 0.2059 | -0.0156 | 0.002 | NA |
| BMI | Yengo | Publication supplement | rs7550711 | T | C | 0.0306 | 0.0649 | 0.005 | NA |
| BMI | Yengo | Publication supplement | rs7551507 | T | C | 0.5633 | -0.0184 | 0.0016 | NA |
| BMI | Yengo | Publication supplement | rs755407 | T | C | 0.2816 | 0.0116 | 0.0018 | NA |
| BMI | Yengo | Publication supplement | rs7557796 | T | C | 0.3476 | 0.016 | 0.0018 | NA |
| BMI | Yengo | Publication supplement | rs7560871 | A | G | 0.0733 | 0.0218 | 0.0034 | NA |
| BMI | Yengo | Publication supplement | rs7561278 | T | C | 0.786 | 0.0159 | 0.0021 | NA |
| BMI | Yengo | Publication supplement | rs7565976 | A | G | 0.3269 | -0.0114 | 0.0019 | NA |
| BMI | Yengo | Publication supplement | rs756717 | A | G | 0.3973 | -0.0148 | 0.0017 | NA |
| BMI | Yengo | Publication supplement | rs7567655 | A | G | 0.969 | -0.027 | 0.0045 | NA |
| BMI | Yengo | Publication supplement | rs7573263 | T | C | 0.4092 | 0.0142 | 0.0018 | NA |
| BMI | Yengo | Publication supplement | rs7578575 | A | T | 0.2974 | 0.013 | 0.0019 | NA |
| BMI | Yengo | Publication supplement | rs7599312 | A | G | 0.2652 | -0.0186 | 0.0019 | NA |
| BMI | Yengo | Publication supplement | rs7600699 | C | G | 0.8421 | -0.0149 | 0.0024 | NA |
| BMI | Yengo | Publication supplement | rs7601895 | C | G | 0.7043 | 0.0149 | 0.0019 | NA |
| BMI | Yengo | Publication supplement | rs7607351 | T | C | 0.5807 | 0.0119 | 0.0017 | NA |
| BMI | Yengo | Publication supplement | rs7607369 | A | G | 0.4387 | 0.0117 | 0.0016 | NA |
| **Trait** | **GWAS author** | **Source** | **SNP** | **Effect Allele** | **Other Allele** | **EAF** | **beta** | **p** | **MR-Base/EBI Study ID** |
| BMI | Yengo | Publication supplement | rs761423 | T | C | 0.55 | 0.0113 | 0.0017 | NA |
| BMI | Yengo | Publication supplement | rs761798 | T | C | 0.3427 | -0.0105 | 0.0018 | NA |
| BMI | Yengo | Publication supplement | rs762147 | A | G | 0.2708 | -0.0115 | 0.0019 | NA |
| BMI | Yengo | Publication supplement | rs7630080 | A | G | 0.9238 | 0.0168 | 0.0031 | NA |
| BMI | Yengo | Publication supplement | rs7630302 | C | G | 0.0997 | -0.0212 | 0.0029 | NA |
| BMI | Yengo | Publication supplement | rs7636868 | A | G | 0.6918 | -0.0112 | 0.0019 | NA |
| BMI | Yengo | Publication supplement | rs7640424 | T | C | 0.2969 | -0.0136 | 0.0018 | NA |
| BMI | Yengo | Publication supplement | rs7652415 | T | C | 0.1417 | 0.0156 | 0.0026 | NA |
| BMI | Yengo | Publication supplement | rs765875 | T | C | 0.4808 | -0.0121 | 0.0017 | NA |
| BMI | Yengo | Publication supplement | rs7674623 | T | C | 0.1976 | 0.0135 | 0.0022 | NA |
| BMI | Yengo | Publication supplement | rs7683836 | A | G | 0.5405 | -0.0114 | 0.0017 | NA |
| BMI | Yengo | Publication supplement | rs7685048 | T | C | 0.4654 | -0.0101 | 0.0017 | NA |
| BMI | Yengo | Publication supplement | rs7694732 | A | G | 0.5622 | 0.0099 | 0.0017 | NA |
| BMI | Yengo | Publication supplement | rs769674 | A | T | 0.6781 | 0.0135 | 0.0018 | NA |
| BMI | Yengo | Publication supplement | rs7715256 | T | G | 0.5781 | -0.0166 | 0.0016 | NA |
| BMI | Yengo | Publication supplement | rs7730004 | T | C | 0.6693 | 0.0148 | 0.0018 | NA |
| BMI | Yengo | Publication supplement | rs7730898 | A | G | 0.729 | 0.0168 | 0.0018 | NA |
| BMI | Yengo | Publication supplement | rs774211 | T | C | 0.8431 | 0.0139 | 0.0022 | NA |
| BMI | Yengo | Publication supplement | rs7748777 | A | G | 0.4587 | 0.0105 | 0.0016 | NA |
| BMI | Yengo | Publication supplement | rs7760082 | A | G | 0.6646 | -0.0122 | 0.0018 | NA |
| BMI | Yengo | Publication supplement | rs7761673 | A | T | 0.2058 | -0.0126 | 0.0021 | NA |
| BMI | Yengo | Publication supplement | rs7770443 | A | C | 0.1534 | 0.0149 | 0.0025 | NA |
| BMI | Yengo | Publication supplement | rs7779498 | T | C | 0.0386 | -0.0249 | 0.0045 | NA |
| BMI | Yengo | Publication supplement | rs7780752 | T | C | 0.64 | -0.0139 | 0.0018 | NA |
| BMI | Yengo | Publication supplement | rs7784465 | T | C | 0.8607 | -0.0164 | 0.0025 | NA |
| BMI | Yengo | Publication supplement | rs779206 | A | G | 0.7336 | -0.0127 | 0.002 | NA |
| BMI | Yengo | Publication supplement | rs7801551 | T | C | 0.6432 | 0.0102 | 0.0018 | NA |
| BMI | Yengo | Publication supplement | rs7802342 | T | G | 0.7104 | -0.0121 | 0.0019 | NA |
| BMI | Yengo | Publication supplement | rs7805441 | T | C | 0.5092 | 0.0109 | 0.0017 | NA |
| **Trait** | **GWAS author** | **Source** | **SNP** | **Effect Allele** | **Other Allele** | **EAF** | **beta** | **p** | **MR-Base/EBI Study ID** |
| BMI | Yengo | Publication supplement | rs7826312 | T | C | 0.4121 | -0.0104 | 0.0017 | NA |
| BMI | Yengo | Publication supplement | rs784944 | A | G | 0.2511 | 0.0122 | 0.0019 | NA |
| BMI | Yengo | Publication supplement | rs7865157 | T | C | 0.1068 | 0.0177 | 0.0028 | NA |
| BMI | Yengo | Publication supplement | rs7869771 | A | C | 0.7353 | 0.014 | 0.0019 | NA |
| BMI | Yengo | Publication supplement | rs7871866 | C | G | 0.1531 | 0.0187 | 0.0024 | NA |
| BMI | Yengo | Publication supplement | rs7874154 | T | C | 0.5021 | -0.0127 | 0.0017 | NA |
| BMI | Yengo | Publication supplement | rs7899106 | A | G | 0.9522 | -0.0331 | 0.0037 | NA |
| BMI | Yengo | Publication supplement | rs7903146 | T | C | 0.2912 | -0.0181 | 0.0018 | NA |
| BMI | Yengo | Publication supplement | rs7925214 | T | C | 0.5133 | 0.0147 | 0.0018 | NA |
| BMI | Yengo | Publication supplement | rs7925748 | A | G | 0.5978 | -0.0122 | 0.0018 | NA |
| BMI | Yengo | Publication supplement | rs7929418 | T | C | 0.3866 | 0.0113 | 0.0018 | NA |
| BMI | Yengo | Publication supplement | rs7933205 | A | G | 0.2115 | -0.0116 | 0.0021 | NA |
| BMI | Yengo | Publication supplement | rs7941030 | T | C | 0.6141 | -0.0112 | 0.0017 | NA |
| BMI | Yengo | Publication supplement | rs7947143 | A | G | 0.1625 | -0.018 | 0.0024 | NA |
| BMI | Yengo | Publication supplement | rs7950748 | A | T | 0.7451 | -0.0118 | 0.002 | NA |
| BMI | Yengo | Publication supplement | rs7973955 | A | G | 0.2847 | -0.0126 | 0.0019 | NA |
| BMI | Yengo | Publication supplement | rs7987314 | T | G | 0.8821 | -0.0146 | 0.0026 | NA |
| BMI | Yengo | Publication supplement | rs8016771 | T | G | 0.9125 | -0.019 | 0.0031 | NA |
| BMI | Yengo | Publication supplement | rs802460 | T | C | 0.6555 | 0.0107 | 0.0018 | NA |
| BMI | Yengo | Publication supplement | rs8033995 | C | G | 0.0937 | -0.0166 | 0.0029 | NA |
| BMI | Yengo | Publication supplement | rs8036040 | A | C | 0.4932 | 0.0109 | 0.0017 | NA |
| BMI | Yengo | Publication supplement | rs8047395 | A | G | 0.5061 | 0.0642 | 0.0017 | NA |
| BMI | Yengo | Publication supplement | rs8061382 | A | T | 0.036 | -0.0307 | 0.0049 | NA |
| BMI | Yengo | Publication supplement | rs8067737 | T | C | 0.1101 | 0.0166 | 0.003 | NA |
| BMI | Yengo | Publication supplement | rs8070454 | T | C | 0.3869 | -0.0098 | 0.0017 | NA |
| BMI | Yengo | Publication supplement | rs8071182 | A | G | 0.1735 | 0.0133 | 0.0022 | NA |
| BMI | Yengo | Publication supplement | rs8081039 | T | C | 0.0573 | 0.0233 | 0.0038 | NA |
| BMI | Yengo | Publication supplement | rs8088123 | A | C | 0.0769 | -0.0262 | 0.0033 | NA |
| BMI | Yengo | Publication supplement | rs8089514 | A | T | 0.359 | 0.0126 | 0.0019 | NA |
| **Trait** | **GWAS author** | **Source** | **SNP** | **Effect Allele** | **Other Allele** | **EAF** | **beta** | **p** | **MR-Base/EBI Study ID** |
| BMI | Yengo | Publication supplement | rs8097672 | A | T | 0.8472 | -0.02 | 0.0025 | NA |
| BMI | Yengo | Publication supplement | rs8123881 | A | G | 0.8701 | -0.0196 | 0.0024 | NA |
| BMI | Yengo | Publication supplement | rs8126575 | T | G | 0.8602 | 0.0149 | 0.0025 | NA |
| BMI | Yengo | Publication supplement | rs818524 | T | C | 0.3061 | -0.0106 | 0.0019 | NA |
| BMI | Yengo | Publication supplement | rs8192675 | T | C | 0.7112 | -0.0152 | 0.0018 | NA |
| BMI | Yengo | Publication supplement | rs820071 | T | G | 0.3485 | -0.0121 | 0.0018 | NA |
| BMI | Yengo | Publication supplement | rs823074 | T | C | 0.5884 | 0.0112 | 0.0017 | NA |
| BMI | Yengo | Publication supplement | rs825680 | A | T | 0.5827 | 0.0104 | 0.0018 | NA |
| BMI | Yengo | Publication supplement | rs833831 | T | G | 0.8716 | 0.016 | 0.0027 | NA |
| BMI | Yengo | Publication supplement | rs845084 | A | G | 0.2678 | 0.014 | 0.002 | NA |
| BMI | Yengo | Publication supplement | rs849135 | A | G | 0.4914 | 0.0109 | 0.0016 | NA |
| BMI | Yengo | Publication supplement | rs852056 | T | C | 0.2416 | 0.0128 | 0.002 | NA |
| BMI | Yengo | Publication supplement | rs857601 | T | G | 0.7189 | 0.0104 | 0.0019 | NA |
| BMI | Yengo | Publication supplement | rs879620 | T | C | 0.6179 | 0.0231 | 0.0018 | NA |
| BMI | Yengo | Publication supplement | rs881301 | T | C | 0.5818 | -0.0097 | 0.0017 | NA |
| BMI | Yengo | Publication supplement | rs889398 | T | C | 0.4247 | -0.0196 | 0.0016 | NA |
| BMI | Yengo | Publication supplement | rs895330 | C | G | 0.8076 | 0.0201 | 0.0023 | NA |
| BMI | Yengo | Publication supplement | rs896183 | A | G | 0.4159 | 0.0102 | 0.0017 | NA |
| BMI | Yengo | Publication supplement | rs901630 | T | C | 0.3973 | -0.0146 | 0.0017 | NA |
| BMI | Yengo | Publication supplement | rs903959 | A | T | 0.3965 | 0.0106 | 0.0018 | NA |
| BMI | Yengo | Publication supplement | rs905938 | T | C | 0.7325 | -0.0149 | 0.0019 | NA |
| BMI | Yengo | Publication supplement | rs9077 | A | G | 0.33 | -0.0138 | 0.0019 | NA |
| BMI | Yengo | Publication supplement | rs925421 | A | G | 0.2657 | 0.0116 | 0.002 | NA |
| BMI | Yengo | Publication supplement | rs9267677 | T | C | 0.8988 | -0.0207 | 0.0028 | NA |
| BMI | Yengo | Publication supplement | rs9294260 | A | G | 0.4731 | 0.0147 | 0.0016 | NA |
| BMI | Yengo | Publication supplement | rs9296723 | T | C | 0.6384 | -0.0106 | 0.0018 | NA |
| BMI | Yengo | Publication supplement | rs9299 | T | C | 0.6471 | 0.0121 | 0.0018 | NA |
| BMI | Yengo | Publication supplement | rs930295 | A | C | 0.1583 | 0.0211 | 0.0023 | NA |
| BMI | Yengo | Publication supplement | rs9318380 | A | G | 0.1567 | 0.0143 | 0.0024 | NA |
| **Trait** | **GWAS author** | **Source** | **SNP** | **Effect Allele** | **Other Allele** | **EAF** | **beta** | **p** | **MR-Base/EBI Study ID** |
| BMI | Yengo | Publication supplement | rs9318686 | T | C | 0.6625 | -0.0105 | 0.0018 | NA |
| BMI | Yengo | Publication supplement | rs9332817 | C | G | 0.0256 | -0.0382 | 0.006 | NA |
| BMI | Yengo | Publication supplement | rs934515 | A | G | 0.1178 | 0.0185 | 0.0027 | NA |
| BMI | Yengo | Publication supplement | rs9349239 | A | G | 0.492 | 0.0122 | 0.0017 | NA |
| BMI | Yengo | Publication supplement | rs9356132 | T | C | 0.6834 | -0.0099 | 0.0018 | NA |
| BMI | Yengo | Publication supplement | rs9361779 | A | C | 0.5572 | 0.011 | 0.0017 | NA |
| BMI | Yengo | Publication supplement | rs936227 | A | G | 0.3892 | -0.0118 | 0.0017 | NA |
| BMI | Yengo | Publication supplement | rs9367368 | T | C | 0.6967 | 0.0121 | 0.0018 | NA |
| BMI | Yengo | Publication supplement | rs9388766 | T | C | 0.2878 | 0.0109 | 0.0018 | NA |
| BMI | Yengo | Publication supplement | rs9397927 | C | G | 0.737 | -0.0119 | 0.002 | NA |
| BMI | Yengo | Publication supplement | rs9408882 | A | G | 0.4594 | -0.0093 | 0.0016 | NA |
| BMI | Yengo | Publication supplement | rs9426003 | A | G | 0.2952 | -0.0116 | 0.0019 | NA |
| BMI | Yengo | Publication supplement | rs945211 | C | G | 0.6363 | 0.013 | 0.0018 | NA |
| BMI | Yengo | Publication supplement | rs9458814 | T | C | 0.7668 | -0.0115 | 0.002 | NA |
| BMI | Yengo | Publication supplement | rs9463175 | T | C | 0.3483 | -0.0108 | 0.0017 | NA |
| BMI | Yengo | Publication supplement | rs946824 | T | C | 0.141 | 0.0206 | 0.0026 | NA |
| BMI | Yengo | Publication supplement | rs9475173 | A | G | 0.6535 | 0.0108 | 0.0019 | NA |
| BMI | Yengo | Publication supplement | rs9478671 | A | G | 0.7913 | -0.012 | 0.0021 | NA |
| BMI | Yengo | Publication supplement | rs9489622 | A | G | 0.516 | 0.0103 | 0.0017 | NA |
| BMI | Yengo | Publication supplement | rs9507983 | T | C | 0.6072 | -0.0156 | 0.0018 | NA |
| BMI | Yengo | Publication supplement | rs9514131 | T | G | 0.1207 | -0.0154 | 0.0026 | NA |
| BMI | Yengo | Publication supplement | rs9527455 | A | C | 0.2323 | -0.0117 | 0.002 | NA |
| BMI | Yengo | Publication supplement | rs9530843 | A | C | 0.5558 | 0.0128 | 0.0018 | NA |
| BMI | Yengo | Publication supplement | rs9538141 | A | G | 0.509 | 0.0164 | 0.0017 | NA |
| BMI | Yengo | Publication supplement | rs954018 | A | G | 0.3053 | -0.013 | 0.0018 | NA |
| BMI | Yengo | Publication supplement | rs9540493 | A | G | 0.4419 | 0.0139 | 0.0017 | NA |
| BMI | Yengo | Publication supplement | rs9547153 | A | G | 0.6161 | -0.0098 | 0.0017 | NA |
| BMI | Yengo | Publication supplement | rs9571687 | A | C | 0.329 | -0.0129 | 0.0018 | NA |
| BMI | Yengo | Publication supplement | rs9615905 | T | C | 0.45 | 0.011 | 0.0017 | NA |
| **Trait** | **GWAS author** | **Source** | **SNP** | **Effect Allele** | **Other Allele** | **EAF** | **beta** | **p** | **MR-Base/EBI Study ID** |
| BMI | Yengo | Publication supplement | rs961917 | C | G | 0.7245 | 0.0116 | 0.002 | NA |
| BMI | Yengo | Publication supplement | rs9644681 | A | G | 0.3471 | 0.0103 | 0.0018 | NA |
| BMI | Yengo | Publication supplement | rs9650755 | A | G | 0.7336 | -0.0154 | 0.002 | NA |
| BMI | Yengo | Publication supplement | rs965961 | A | G | 0.1027 | 0.0172 | 0.0028 | NA |
| BMI | Yengo | Publication supplement | rs9688431 | T | C | 0.9397 | 0.0231 | 0.0035 | NA |
| BMI | Yengo | Publication supplement | rs972283 | A | G | 0.4849 | 0.0096 | 0.0016 | NA |
| BMI | Yengo | Publication supplement | rs977540 | A | G | 0.7617 | 0.014 | 0.0019 | NA |
| BMI | Yengo | Publication supplement | rs977747 | T | G | 0.4051 | 0.0169 | 0.0017 | NA |
| BMI | Yengo | Publication supplement | rs9806058 | A | T | 0.877 | 0.0164 | 0.0026 | NA |
| BMI | Yengo | Publication supplement | rs9814557 | A | G | 0.6776 | -0.01 | 0.0018 | NA |
| BMI | Yengo | Publication supplement | rs9814633 | A | G | 0.3434 | 0.0122 | 0.0018 | NA |
| BMI | Yengo | Publication supplement | rs9816226 | A | T | 0.1801 | -0.0323 | 0.0021 | NA |
| BMI | Yengo | Publication supplement | rs9817583 | A | G | 0.1703 | 0.0137 | 0.0023 | NA |
| BMI | Yengo | Publication supplement | rs9827823 | T | C | 0.8511 | 0.0193 | 0.0024 | NA |
| BMI | Yengo | Publication supplement | rs9845966 | T | G | 0.4521 | 0.0105 | 0.0017 | NA |
| BMI | Yengo | Publication supplement | rs9848399 | A | G | 0.8754 | 0.0164 | 0.0026 | NA |
| BMI | Yengo | Publication supplement | rs987237 | A | G | 0.8197 | -0.0409 | 0.0021 | NA |
| BMI | Yengo | Publication supplement | rs9904177 | A | G | 0.7262 | -0.0117 | 0.002 | NA |
| BMI | Yengo | Publication supplement | rs9921416 | T | C | 0.4407 | -0.01 | 0.0018 | NA |
| BMI | Yengo | Publication supplement | rs9951893 | T | C | 0.5249 | -0.0115 | 0.0017 | NA |
| BMI | Yengo | Publication supplement | rs995258 | A | C | 0.5779 | 0.0144 | 0.0017 | NA |
| BMI | Yengo | Publication supplement | rs9964756 | T | G | 0.1033 | -0.0164 | 0.0028 | NA |
| BMI | Yengo | Publication supplement | rs9965170 | A | G | 0.429 | -0.0097 | 0.0017 | NA |
| BMI | Yengo | Publication supplement | rs998732 | A | G | 0.8422 | 0.0171 | 0.0022 | NA |
| BMI | Yengo | Publication supplement | rs9989141 | T | C | 0.6387 | 0.0162 | 0.0017 | NA |
| BMI | Yengo | Publication supplement | rs999889 | A | G | 0.2818 | -0.0108 | 0.0019 | NA |
| ^1^Associations based on Europeans only. ^2^Associations based on sample including 23andMe. | | | | | | | | | |

| **Supplementary Table 5: Descriptive Characteristics of Analytic Sample: unimputed data^1^** | | | | | |
| --- | --- | --- | --- | --- | --- |
| **Continuous variables** | **Total observations** | **mean** | **SD** | **min** | **max** |
| Maternal  age | 5,689 | 28.32 | 4.66 | 15.00 | 44.00 |
| SDQ hyperactivity score^2^ at 10 (114 months) | 4,248 | 2.86 | 2.20 | 0.00 | 10.00 |
| SDQ hyperactivity score^2^ at 13 (156 months) | 3,959 | 2.89 | 2.20 | 0.00 | 10.00 |
| MFQ score^3^ at 10 (127 months) | 4,432 | 3.97 | 3.44 | 0.00 | 21.00 |
| MFQ score^3^ at 13 (154 months) | 4,160 | 3.95 | 3.88 | 0.00 | 24.00 |
| SCDC score^4^ at 10 (120 months) | 4,372 | 2.23 | 3.40 | 0.00 | 24.00 |
| SCDC score^4^ at 13 (156 months) | 4,093 | 2.49 | 3.54 | 0.00 | 24.00 |
| BMI^5^ z-score at 10 (127 months) | 4,468 | 0.31 | 1.15 | -3.49 | 3.61 |
| BMI^5^ z-score at 13 (154 months) | 4,116 | 0.36 | 1.18 | -3.75 | 3.74 |
| GCSE capped points score | 6,113 | 332.34 | 87.36 | 0.00 | 540.00 |
| Absenteeism at year 11 (age 15-16), % | 5,702 | 7.63 | 8.77 | 0.00 | 98.59 |
| Absenteeism at year 10 (age 14-15), % | 4,520 | 6.51 | 7.56 | 0.00 | 84.54 |
| Absenteeism at key stage 4 (age 14-16), % | 4,472 | 6.91 | 7.09 | 0.00 | 74.61 |
| **Categorical variables** | **Total observations** | **Category** | | **N** | **%** |
| gender | 6,113 | male | | 3056 | 49.99 |
|  |  | female | | 3057 | 50.01 |
| maternal educational qualifications | 5,527 | CSE or less | | 880 | 15.92 |
|  |  | vocational | | 519 | 9.39 |
|  |  | O level | | 2000 | 36.19 |
|  |  | A level | | 1,365 | 24.70 |
|  |  | Degree | | 763 | 13.80 |
| maternal housing tenure in pregnancy | 5,964 | mortgage/owned outright | | 4,800 | 80.48 |
|  |  | council rented | | 607 | 10.18 |
|  |  | private/other rented | | 405 | 6.79 |
|  |  | other | | 152 | 2.55 |
| maternal parity at child’s birth | 5,597 | 0 | | 2,519 | 45.01 |
|  |  | 1 | | 2,030 | 36.27 |
|  |  | 2 | | 778 | 13.90 |
|  |  | 3+ | | 270 | 4.82 |
| mother smoked during pregnancy | 5,698 | no | | 4,404 | 77.29 |
|  |  | yes | | 1,294 | 22.71 |
| migraines at 10 | 4,354 | no | | 4,164 | 95.64 |
|  |  | yes | | 190 | 4.36 |
| asthma at 10 (128 months) | 4,342 | no | | 3,835 | 88.32 |
|  |  | yes | | 507 | 11.68 |
| asthma at 13 (157 months) | 4,013 | no | | 3,544 | 88.31 |
|  |  | yes | | 469 | 11.69 |
| school type^6^ at key stage 4 (age 14-16) | 6,113 | mainstream state | | 5,693 | 93.13 |
|  |  | independent | | 333 | 5.45 |
|  |  | other | | 87 | 1.42 |
| ^1^Analysis was restricted to unrelated ALSPAC participants with genetic data and GCSE records, N=6,113. ^2^SDQ=Strengths and Difficulties Questionnaire, for ADHD symptoms.  ^3^MFQ=Mood and Feelings Questionnaire, for depressive symptoms. ^4^SCDC=Social Communication Disorder Checklist, for autistic social traits. ^5^Using 1990 UK Growth Reference. Values represent standard deviation difference from age- and gender-specific reference mean. ^6^Mainstream state schools: community, voluntary controlled or aided, foundation, city technology college, academy. Other schools: community special, pupil referral unit, further education college. | | | | | |

| **Supplementary Table 6: Association of GCSEs and absenteeism at key stage 4 (age 14-16) with covariates** | | | | | |
| --- | --- | --- | --- | --- | --- |
|  |  | GCSE points | | Absenteeism at age 14-16 | |
|  |  | Beta | CI | % change | CI |
| Gender | male | Ref |  | Ref |  |
|  | female | 26.6 | 21.0,32.2 | 9.9 | 5.1, 14.9 |
| *Associations below are gender-adjusted* | |  |  |  |  |
| maternal educational qualifications | CSE or less | Ref |  | Ref |  |
|  | vocational | 21.5 | 10.9,32.1 | -10.2 | -18.0,-1.70 |
|  | O level | 52.6 | 44.8,60.5 | -21.8 | -27.3,-15.9 |
|  | A level | 80.8 | 71.2,90.3 | -24.8 | -31.2,-17.9 |
|  | Degree | 116.9 | 106.5,127.3 | -30.3 | -38.7,-20.8 |
| maternal housing tenure in pregnancy | mortgage/owned outright | Ref |  | Ref |  |
|  | council rented | -92.8 | -103.6,-81.9 | 53.1 | 39.8,67.7 |
|  | private/other rented | -41.5 | -51.8,-31.3 | 29.2 | 18.2,41.3 |
|  | other | -26.7 | -41.4,-12.0 | 11.3 | -3.7,28.6 |
| maternal parity at child’s birth | 0 | Ref |  | Ref |  |
|  | 1 | -9.6 | -14.8,-4.4 | 8 | 3.6,12.6 |
|  | 2 | -28.5 | -35.5,-21.4 | 22.2 | 14.2,30.7 |
|  | 3+ | -43.8 | -57.5,-30.0 | 34 | 20.2,49.5 |
| mother smoked during pregnancy | no | Ref |  | Ref |  |
|  | yes | -48.2 | -55.5,-40.8 | 29.8 | 21.2,38.9 |
| school type^1^ at key stage 4 (age 14-16) | mainstream state | Ref |  | Ref |  |
|  | independent | 62.9 | 45.5,80.4 | 64.2 | -0.80,171.8 |
|  | other | -214.0 | -247.2,-180.8 | 103.4 | 52.1,172.1 |
| Maternal age (per year) | | 4.7 | 4.1,5.3 | -1.2 | -1.1 |
| School absence (per extra day of school/year^2^) | | -2.7 | -3.3,-2.0 |  |  |
| ^1^Mainstream state schools: community, voluntary controlled or aided, foundation, city technology college, academy. Other schools: community special, pupil referral unit, further education college.  ^2^Assuming 190 days in a school year, standard for UK state schools. | | | | | |

| **Supplementary Table 7: School type checks: health at 10 and 13 to GCSE points score and absenteeism at age 14-16^1^** | | | | | | | | | | |
| --- | --- | --- | --- | --- | --- | --- | --- | --- | --- | --- |
|  |  | **Mainstream state schools** | | | **Independent schools** | | | **Other schools** | |  |
|  |  | GCSE points (range 0-540) | | | GCSE points (range 0-540) | | | GCSE points (range 0-540) | | |
|  | Age | Beta | lci | uci | Beta | lci | uci | Beta | lci | uci |
| Standardized values of SDQ-HI score^2^ | 10 | -22.69 | -25.15 | -20.24 | -12.32 | -19.02 | -5.63 | -34.68 | -59.59 | -9.77 |
| Standardized values of MFQ score^3^ | 10 | -12.76 | -15.21 | -10.30 | -7.44 | -14.41 | -0.48 | -22.34 | -42.71 | -1.98 |
| Standardized values of SCDC score^4^ | 10 | -13.90 | -16.61 | -11.20 | -5.88 | -13.59 | 1.83 | -19.67 | -37.39 | -1.96 |
| Mother report age 10: child gets migraines | 10 | -2.68 | -14.80 | 9.44 | -20.42 | -48.26 | 7.42 | -11.14 | -98.98 | 76.69 |
| Asthma in past 12 months | 10 | 1.75 | -5.27 | 8.77 | 5.62 | -12.53 | 23.76 | 33.89 | -40.66 | 108.44 |
| BMI z-score | 10 | -1.98 | -3.78 | -0.19 | -2.39 | -9.33 | 4.55 | -4.71 | -23.71 | 14.28 |
| Standardized values of SDQ-HI score^2^ | 13 | -25.31 | -27.80 | -22.83 | -17.44 | -24.71 | -10.17 | -36.91 | -61.56 | -12.27 |
| Standardized values of MFQ score^3^ | 13 | -4.34 | -6.82 | -1.85 | -0.31 | -5.71 | 5.08 | -10.07 | -29.19 | 9.05 |
| Standardized values of SCDC score^4^ | 13 | -17.82 | -20.39 | -15.24 | -10.07 | -20.39 | 0.24 | -20.16 | -41.45 | 1.13 |
| Asthma in past 12 months | 13 | -0.26 | -7.93 | 7.41 | 9.02 | -8.39 | 26.43 | 27.36 | -36.06 | 90.79 |
| BMI z-score | 13 | -3.68 | -5.51 | -1.86 | -5.50 | -12.06 | 1.06 | -10.24 | -30.21 | 9.72 |
|  |  | **Mainstream state schools** | | | **Independent schools** | | | **Other schools** | |  |
|  |  | Absence at key stage 4 (14-16) | | | Absence at key stage 4 (14-16) | | | Absence at key stage 4 (14-16) | | |
|  | Age | % | lci | uci | % | lci | uci | % | lci | uci |
| Standardized values of SDQ-HI score^2^ | 10 | 6.05 | 3.47 | 8.70 | 0.37 | -10.81 | 12.95 | -2.44 | -26.17 | 28.92 |
| Standardized values of MFQ score^3^ | 10 | 5.82 | 3.10 | 8.62 | 4.07 | -8.32 | 18.14 | -0.8 | -19.91 | 22.88 |
| Standardized values of SCDC score^4^ | 10 | 9.08 | 6.21 | 12.02 | 4.62 | -10.28 | 22.00 | 4.73 | -13.26 | 26.45 |
| Mother report age 10: child gets migraines | 10 | 12.77 | 1.51 | 25.28 | 26.25 | -19.68 | 98.46 | -4.57 | -60.4 | 129.99 |
| Asthma in past 12 months | 10 | 11.87 | 4.29 | 20.01 | 12.9 | -16.75 | 53.11 | 26.48 | -42.59 | 178.64 |
| BMI z-score | 10 | 2.41 | 0.60 | 4.26 | 2.96 | -7.38 | 14.45 | 7.57 | -16.11 | 37.94 |
| Standardized values of SDQ-HI score^2^ | 13 | 8.86 | 6.24 | 11.54 | 4.09 | -7.64 | 17.31 | 3.92 | -21.32 | 37.26 |
| Standardized values of MFQ score^3^ | 13 | 6.91 | 4.54 | 9.33 | 5.71 | -4.75 | 17.31 | 2.88 | -15.26 | 24.91 |
| Standardized values of SCDC score^4^ | 13 | 12.87 | 9.63 | 16.21 | 8.62 | -6.78 | 26.56 | 7.71 | -12.91 | 33.22 |
| Asthma in past 12 months | 13 | 9.28 | 1.54 | 17.60 | 9.02 | -17.14 | 43.45 | 44.77 | -28.11 | 191.56 |
| BMI z-score, using UK 1990 growth reference chart | 13 | 3.69 | 1.89 | 5.52 | 4.85 | -5.12 | 15.88 | 9.13 | -13.65 | 37.92 |
| ^1^Adjusted for gender, maternal education, maternal housing tenure, maternal age, maternal parity, whether smoked in pregnancy. Coefficients for log-transformed days were exponentiated to obtain % change. ^2^SDQ-HI: Strengths and Difficulties Questionnaire hyperactivity subscale. ^3^Mood and Feelings Questionnaire. ^4^Social Communication Disorders Checklist. | | | | | | | | | | |

| **Supplementary Table 8: School type checks: associations of health conditions with educational attainment - mediation by school absence at age 14-16** | | | | | | | | | | | | |
| --- | --- | --- | --- | --- | --- | --- | --- | --- | --- | --- | --- | --- |
|  |  |  | **Mainstream state schools only^1^** | | | | | **Independent schools** | | | | |
| Exposure | Age |  | Beta^2^ | LCI | UCI | p | % | Beta^2^ | LCI | UCI | p | % |
| SDQ-HI score^3^ for ADHD | 10 | direct | -21.40 | -23.70 | -19.10 | <0.001 |  | -12.31 | -19.22 | -5.41 | <0.001 |  |
|  |  | indirect | -1.90 | -2.97 | -0.83 | <0.001 |  | -0.06 | -2.82 | 2.69 | 0.96 |  |
|  |  | total | -23.30 | -25.73 | -20.87 | <0.001 | 8.16 | -12.38 | -19.47 | -5.29 | <0.001 | 0.52 |
| MFQ score^4^ for depressive symptoms | 10 | direct | -12.33 | -14.98 | -9.69 | <0.001 |  | -7.38 | -14.34 | -0.41 | 0.04 |  |
|  |  | indirect | -1.89 | -3.05 | -0.73 | 0.001 |  | -0.88 | -3.81 | 2.05 | 0.56 |  |
|  |  | total | -14.22 | -17.00 | -11.45 | <0.001 | 13.29 | -8.26 | -15.41 | -1.10 | 0.02 | 10.65 |
| SCDC score^5^ for autistic social traits | 10 | direct | -14.28 | -16.84 | -11.72 | <0.001 |  | -5.54 | -13.86 | 2.78 | 0.19 |  |
|  |  | indirect | -2.84 | -4.05 | -1.63 | <0.001 |  | -1.00 | -4.30 | 2.30 | 0.55 |  |
|  |  | total | -17.12 | -19.77 | -14.47 | <0.001 | 16.59 | -6.54 | -15.11 | 2.04 | 0.14 | 15.29 |
| Asthma in past 12 months | 10 | direct | 5.77 | -0.37 | 11.91 | 0.07 |  | 8.23 | -9.23 | 25.69 | 0.36 |  |
|  |  | indirect | -3.91 | -6.47 | -1.34 | 0.003 |  | -2.85 | -9.13 | 3.44 | 0.37 |  |
|  |  | total | 1.86 | -4.58 | 8.31 | 0.57 | -209.91 | 5.38 | -12.67 | 23.43 | 0.56 | -52.91 |
| BMI z-score^6^ | 10 | direct | -1.49 | -3.53 | 0.54 | 0.15 |  | -1.58 | -7.80 | 4.65 | 0.62 |  |
|  |  | indirect | -0.82 | -1.78 | 0.13 | 0.09 |  | -0.69 | -3.30 | 1.92 | 0.61 |  |
|  |  | total | -2.32 | -4.42 | -0.22 | 0.03 | 35.56 | -2.26 | -8.66 | 4.13 | 0.49 | 30.38 |
| Migraines at 10 | 10 | direct | 1.29 | -7.71 | 10.28 | 0.78 |  | -16.21 | -41.04 | 8.62 | 0.20 |  |
|  |  | indirect | -4.17 | -7.79 | -0.55 | 0.02 |  | -5.26 | -13.84 | 3.32 | 0.23 |  |
|  |  | total | -2.88 | -12.36 | 6.59 | 0.55 | 144.55 | -21.47 | -47.08 | 4.14 | 0.10 | 24.50 |
| SDQ-HI score^3^ for ADHD | 13 | direct | -23.80 | -26.08 | -21.52 | <0.001 |  | -16.11 | -22.87 | -9.35 | <0.001 |  |
|  |  | indirect | -2.63 | -3.70 | -1.55 | <0.001 |  | -0.85 | -3.58 | 1.89 | 0.54 |  |
|  |  | total | -26.42 | -28.81 | -24.04 | <0.001 | 9.94 | -16.96 | -23.91 | -10.00 | 0.00 | 5.00 |
| MFQ score^4^ for depressive symptoms | 13 | direct | -3.22 | -5.82 | -0.62 | 0.02 |  | 0.94 | -5.25 | 7.12 | 0.77 |  |
|  |  | indirect | -2.29 | -3.46 | -1.12 | <0.001 |  | -1.28 | -3.89 | 1.33 | 0.34 |  |
|  |  | total | -5.52 | -8.22 | -2.81 | <0.001 | 41.53 | -0.35 | -6.71 | 6.01 | 0.92 | 370.19 |

|  |  |  | **Mainstream state schools only^1^** | | | | | **Independent schools** | | | | |
| --- | --- | --- | --- | --- | --- | --- | --- | --- | --- | --- | --- | --- |
| Exposure | Age |  | Beta^2^ | LCI | UCI | p | % | Beta^2^ | LCI | UCI | p | % |
| SCDC score^5^ for autistic social traits | 13 | direct | -18.39 | -21.06 | -15.71 | <0.001 |  | -9.32 | -18.08 | -0.55 | 0.04 |  |
|  |  | indirect | -3.75 | -4.97 | -2.52 | <0.001 |  | -1.83 | -5.23 | 1.57 | 0.29 |  |
|  |  | total | -22.13 | -24.88 | -19.38 | <0.001 | 16.92 | -11.15 | -20.20 | -2.10 | 0.02 | 16.43 |
| Asthma in past 12 months | 13 | direct | 2.70 | -3.59 | 8.98 | 0.40 |  | 10.70 | -6.30 | 27.71 | 0.22 |  |
|  |  | indirect | -3.08 | -5.73 | -0.43 | 0.02 |  | -1.99 | -7.90 | 3.92 | 0.51 |  |
|  |  | total | -0.38 | -6.96 | 6.20 | 0.91 | 804.64 | 8.71 | -8.95 | 26.37 | 0.33 | -22.87 |
| BMI z-score^6^ | 13 | direct | -2.73 | -4.77 | -0.69 | 0.01 |  | -4.24 | -10.26 | 1.78 | 0.17 |  |
|  |  | indirect | -1.24 | -2.18 | -0.31 | 0.01 |  | -1.10 | -3.64 | 1.44 | 0.40 |  |
|  |  | total | -3.97 | -6.10 | -1.85 | <0.001 | 31.32 | -5.34 | -11.49 | 0.82 | 0.09 | 20.57 |
| ^2^For binary exposures, coefficients represent change associated with presence vs absence of the health condition. For continuous exposures, coefficients represent change associated with a 1SD increase from sample mean.^3^SDQ-HI: Strengths and Difficulties Questionnaire hyperactivity subscale. ^4^MFQ: Mood and Feelings Questionnaire. ^5^SCDC: Social Communication Disorders Checklist. ^6^Using 1990 UK Growth Reference. | | | | | | | | | | | | |

| **Supplementary Table 8 (continued): School type checks: associations of child health with GCSE points score - mediation by absenteeism at key stage 4 (age 14-16)** | | | | | | | |
| --- | --- | --- | --- | --- | --- | --- | --- |
|  |  |  | **Other schools** | |  |  |  |
| Exposure | Age |  | Beta^1^ | LCI | UCI | p | % |
| SDQ-HI score^2^ for ADHD | 10 | direct | -38.57 | -57.54 | -19.60 | <0.001 |  |
|  |  | indirect | 0.64 | -4.32 | 5.60 | 0.80 |  |
|  |  | total | -37.93 | -57.32 | -18.54 | <0.001 | -1.69 |
| MFQ score^3^ for depressive symptoms | 10 | direct | -21.82 | -38.43 | -5.21 | 0.01 |  |
|  |  | indirect | 0.37 | -3.94 | 4.68 | 0.87 |  |
|  |  | total | -21.46 | -38.31 | -4.60 | 0.01 | -1.70 |
| SCDC score^4^ for autistic social traits | 10 | direct | -17.68 | -31.03 | -4.33 | 0.01 |  |
|  |  | indirect | -0.63 | -3.77 | 2.51 | 0.70 |  |
|  |  | total | -18.30 | -31.80 | -4.81 | 0.01 | 3.43 |
| Asthma in past 12 months | 10 | direct | 32.53 | -29.23 | 94.28 | 0.30 |  |
|  |  | indirect | -4.86 | -20.35 | 10.64 | 0.54 |  |
|  |  | total | 27.67 | -34.79 | 90.13 | 0.39 | -17.55 |
| BMI z-score^5^ | 10 | direct | -4.63 | -23.02 | 13.77 | 0.62 |  |
|  |  | indirect | -1.26 | -5.86 | 3.34 | 0.59 |  |
|  |  | total | -5.88 | -24.45 | 12.68 | 0.53 | 21.37 |
| Migraines at 10 | 10 | direct | -10.41 | -84.35 | 63.53 | 0.78 |  |
|  |  | indirect | 1.38 | -15.24 | 18.00 | 0.87 |  |
|  |  | total | -9.03 | -84.08 | 66.01 | 0.81 | -15.28 |
| SDQ-HI score^2^ for ADHD | 13 | direct | -39.93 | -57.23 | -22.63 | 0.00 |  |
|  |  | indirect | -0.43 | -4.62 | 3.77 | 0.84 |  |
|  |  | total | -40.36 | -57.91 | -22.81 | <0.001 | 1.06 |
| MFQ score^3^ for depressive symptoms | 13 | direct | -13.24 | -30.03 | 3.56 | 0.12 |  |
|  |  | indirect | -0.41 | -4.37 | 3.55 | 0.84 |  |
|  |  | total | -13.64 | -30.65 | 3.36 | 0.12 | 3.00 |
| SCDC score^4^ for autistic social traits | 13 | direct | -18.17 | -32.84 | -3.51 | 0.02 |  |
|  |  | indirect | -0.98 | -4.57 | 2.61 | 0.59 |  |
|  |  | total | -19.15 | -33.87 | -4.43 | 0.01 | 5.12 |
| Asthma in past 12 months | 13 | direct | 33.25 | -21.34 | 87.84 | 0.23 |  |
|  |  | indirect | -7.88 | -23.82 | 8.06 | 0.33 |  |
|  |  | total | 25.37 | -29.43 | 80.18 | 0.36 | -31.05 |
| BMI z-score^5^ | 13 | direct | -9.25 | -27.02 | 8.51 | 0.31 |  |
|  |  | indirect | -1.42 | -5.90 | 3.06 | 0.54 |  |
|  |  | total | -10.67 | -28.53 | 7.18 | 0.24 | 13.30 |
| ^1^For binary exposures, coefficients represent change associated with presence vs absence of the health condition. For continuous exposures, coefficients represent change associated with a 1SD increase from sample mean.^2^SDQ-HI: Strengths and Difficulties Questionnaire hyperactivity subscale. ^3^MFQ: Mood and Feelings Questionnaire. ^4^SCDC: Social Communication Disorders Checklist. ^5^Using 1990 UK Growth Reference. | | | | | | | |

| **Supplementary Table 9: Prediction of phenotypes by polygenic scores in ALSPAC** | | |
| --- | --- | --- |
|  | **R2 or pseudo-R2** | |
|  | Age 10 | Age 13 |
| ADHD (SDQ-HI score) | 0.0012 | 0.0011 |
| ASD (SCSC score) | 0.0001 | 0.0001 |
| Depressive symptoms (MFQ score) | 0.0001 | 0.0005 |
| Asthma | 0.0061 | 0.0075 |
| Migraine | 0.0074 |  |
| BMI z-score | 0.0742 | 0.077 |
|  | **F-statistic^1^ from first stage of 2SLS model** | |
|  | Age 10 | Age 13 |
| ADHD (SDQ-HI score) | 6.34 | 6.7 |
| ASD (SCSC score) | 0.66 | 0.94 |
| Depressive symptoms (MFQ score) | 0.48 | 5.19 |
| Asthma | 27.73 | 32.73 |
| Migraine | 19.16 |  |
| BMI z-score | 494.45 | 517.91 |
| **^1^**Calculated within each imputed dataset and combined across imputations. | | |

| **Supplementary Table 10: School type checks: association of polygenic scores^1^ with GCSE points score and absenteeism at age 14-16** | | | | | | | | | |
| --- | --- | --- | --- | --- | --- | --- | --- | --- | --- |
|  | **Mainstream state schools^2^** | | | **Independent schools** | | | **Other schools^3^** | | |
|  | GCSE points score | | | GCSE points score | | | GCSE points score | | |
|  | Beta | LCI | UCI | Beta | LCI | UCI | Beta | LCI | UCI |
| Standardized ADHD PGS | -2.41 | -4.35 | -0.47 | -0.65 | -6.32 | 5.01 | -0.55 | -25.36 | 24.26 |
| Standardized depression PGS | 0.71 | -1.83 | 3.24 | 4.54 | -0.55 | 9.62 | -3.97 | -24.32 | 16.38 |
| Standardized ASD PGS | -2.06 | -4.10 | -0.01 | 1.53 | -4.11 | 7.16 | -4.24 | -27.63 | 19.14 |
| Standardized migraine PGS | -1.54 | -3.67 | 0.59 | 1.55 | -4.13 | 7.24 | 20.32 | -12.26 | 52.89 |
| Standardized asthma PGS | -0.92 | -3.03 | 1.18 | 3.11 | -4.50 | 10.72 | 13.85 | -15.74 | 43.43 |
| Standardized BMI PGS | -5.31 | -7.58 | -3.04 | -4.39 | -11.26 | 2.48 | 15.91 | -12.62 | 44.45 |
|  | School absence at 14-16 | | | School absence at 14-16 | | | School absence at 14-16 | | |
|  | % | LCI | UCI | % | LCI | UCI | % | LCI | UCI |
| Standardized ADHD PGS | 0.05 | -1.86 | 1.99 | -1.49 | -10.09 | 7.93 | -10.31 | -29.46 | 14.04 |
| Standardized depression PGS | -0.68 | -2.76 | 1.44 | -1.77 | -10.61 | 7.94 | -0.81 | -21.42 | 25.20 |
| Standardized ASD PGS | -0.23 | -2.24 | 1.83 | -1.21 | -12.09 | 11.00 | -19.77 | -38.74 | 5.08 |
| Standardized migraine PGS | 1.17 | -0.79 | 3.16 | 1.34 | -8.48 | 12.22 | 4.00 | -16.85 | 30.06 |
| Standardized asthma PGS | 1.11 | -0.64 | 2.90 | 0.17 | -8.98 | 10.24 | 8.49 | -17.47 | 42.63 |
| Standardized BMI PGS | 3.02 | 0.90 | 5.18 | 1.54 | -7.58 | 11.56 | 6.56 | -15.74 | 34.77 |
| ^1^Adjusted for gender and PC1-PC20. Coefficients for log-transformed days were exponentiated to obtain % change. ^2^Mainstream state schools: community, voluntary controlled or aided, foundation, city technology college, academy. ^3^Other schools: community special, pupil referral unit, further education college | | | | | | | | | |

| **Supplementary Table 11: Robustness checks for individual-participant analyses in ALSPAC** **using Two-Sample methodology^1^** | | | | | | | | | | | | | | | |
| --- | --- | --- | --- | --- | --- | --- | --- | --- | --- | --- | --- | --- | --- | --- | --- |
| **Outcome: GCSE point score** | | | | | | | | | | | | | | | |
|  | IVW | | | MR-Egger | | | | | | MR-median | | | MR-modal | | |
|  | Beta | se | p | Beta: slope | se | p | Beta: intercept | se | p | Beta | se | p | Beta | se | p |
| ADHD | -16.29 | 6.65 | 0.01 | -0.76 | 35.41 | 0.98 | -1.51 | 3.22 | 0.64 | -14.48 | 9.05 | 0.11 | -11.76 | 12.03 | 0.33 |
| ASD | -11.41 | 6.29 | 0.07 | 23.29 | 18.42 | 0.21 | -3.37 | 1.72 | 0.05 | -13.42 | 8.32 | 0.11 | -16.19 | 12.58 | 0.20 |
| Depressive symptoms | 5.17 | 11.89 | 0.66 | -62.32 | 79.66 | 0.43 | 2.17 | 2.52 | 0.39 | -12.28 | 13.80 | 0.37 | -39.71 | 33.08 | 0.23 |
| Asthma | -2.91 | 3.88 | 0.45 | -84.57 | 48.43 | 0.08 | 12.59 | 7.46 | 0.09 | -0.07 | 5.79 | 0.99 | 3.79 | 8.91 | 0.67 |
| Migraine | -1.14 | 4.74 | 0.81 | -3.53 | 14.97 | 0.81 | -0.01 | 0.96 | 1.00 | -4.39 | 7.70 | 0.57 | 10.06 | 13.56 | 0.46 |
| BMI | -18.95 | 4.18 | <0.001 | -3.44 | 12.35 | 0.78 | -0.23 | 0.18 | 0.21 | -13.27 | 6.33 | 0.04 | -3.33 | 16.32 | 0.84 |
| **Outcome: school absence at age 14-16** | | | | | |  |  |  |  |  |  |  |  |  |  |
|  | IVW | | | MR-Egger | | | | | | MR-median | | | MR-modal | | |
|  | Beta | se | p | Beta: slope | se | p | Beta: intercept | se | p | Beta | se | p | Beta | se | p |
| ADHD | -0.02 | 0.06 | 0.78 | -0.28 | 0.28 | 0.32 | 0.03 | 0.03 | 0.32 | 0.03 | 0.08 | 0.68 | 0.06 | 0.11 | 0.58 |
| ASD | -0.02 | 0.06 | 0.76 | -0.14 | 0.17 | 0.39 | 0.01 | 0.02 | 0.44 | -0.08 | 0.07 | 0.29 | -0.08 | 0.09 | 0.37 |
| Depressive symptoms | -0.06 | 0.08 | 0.46 | 0.60 | 0.50 | 0.23 | -0.02 | 0.02 | 0.20 | -0.04 | 0.11 | 0.67 | 0.02 | 0.22 | 0.91 |
| Asthma | 0.05 | 0.06 | 0.44 | 1.16 | 0.45 | 0.01 | -0.17 | 0.07 | 0.01 | 0.08 | 0.06 | 0.15 | 0.13 | 0.08 | 0.10 |
| Migraine | 0.01 | 0.05 | 0.81 | 0.03 | 0.15 | 0.87 | 0.003 | 0.01 | 0.74 | 0.05 | 0.07 | 0.50 | 0.18 | 0.14 | 0.22 |
| BMI | 0.10 | 0.03 | 0.004 | -0.14 | 0.10 | 0.17 | 0.004 | 0.001 | 0.02 | 0.05 | 0.06 | 0.37 | -0.08 | 0.15 | 0.60 |
| ^1^SNP-exposure betas taken from GWAS listed in Supplementary Table 2. SNP-outcome betas calculated in ALSPAC, by regressing GCSE points score on each SNP adjusted for gender and PC1-20. | | | | | | | | | | | | | | | |

| **Supplementary Table 12: SNP-specific ratio estimates for the effect of BMI on school absence** | | | |
| --- | --- | --- | --- |
| **rsid** | **Beta** | **se** | **p (difference)^1^** |
| rs10007906 | 3.935823 | 1.218904 | 0.001638 |
| rs11713193 | 1.880117 | 0.570883 | 0.00181 |
| rs12564992 | -2.89008 | 1.024175 | 0.003567 |
| rs6849518 | 3.479809 | 1.166919 | 0.003749 |
| rs4148866 | -4.83294 | 1.707125 | 0.003892 |
| rs2235564 | -3.3195 | 1.214682 | 0.004941 |
| rs1431659 | -1.92448 | 0.759155 | 0.007838 |
| rs2307111 | 1.324492 | 0.487676 | 0.011966 |
| rs7865157 | 3.000093 | 1.182623 | 0.014103 |
| rs1412235 | -1.25719 | 0.562438 | 0.016314 |
| rs1528435 | 2.326729 | 0.929967 | 0.016524 |
| rs610634 | -3.24047 | 1.403545 | 0.017476 |
| rs4989244 | -3.12085 | 1.35695 | 0.01779 |
| rs9571687 | -2.67441 | 1.187261 | 0.019671 |
| rs13174863 | 2.455598 | 1.02004 | 0.020779 |
| rs7560871 | -2.74365 | 1.23257 | 0.021277 |
| rs7421089 | 2.656891 | 1.119193 | 0.02219 |
| rs7123876 | 3.38832 | 1.45851 | 0.024027 |
| rs9267677 | 2.772204 | 1.189684 | 0.024538 |
| rs2122042 | 1.750721 | 0.737546 | 0.025013 |
| rs543874 | -0.63947 | 0.327144 | 0.025303 |
| rs7903146 | -1.75649 | 0.834232 | 0.026495 |
| rs12065553 | 2.864311 | 1.2528 | 0.027183 |
| rs6449532 | -2.15415 | 1.02103 | 0.027618 |
| rs2304130 | -2.84217 | 1.339081 | 0.02827 |
| rs7947143 | -2.13493 | 1.026423 | 0.029825 |
| rs11264483 | 2.212124 | 0.97527 | 0.030123 |
| rs6433243 | 3.519606 | 1.582467 | 0.030545 |
| rs4518345 | -2.69856 | 1.297785 | 0.031346 |
| rs2923774 | -2.81834 | 1.357162 | 0.031812 |
| rs8081039 | -2.52887 | 1.256867 | 0.036823 |
| rs3852012 | 2.741575 | 1.271665 | 0.037557 |
| rs7138803 | -0.9432 | 0.49914 | 0.03776 |
| rs9540493 | -2.11043 | 1.067117 | 0.038763 |
| rs11084553 | -1.70584 | 0.881253 | 0.041031 |
| rs1884389 | -2.80985 | 1.424492 | 0.041411 |
| rs1865341 | -2.39295 | 1.221879 | 0.041723 |
| rs13107325 | 1.410892 | 0.645047 | 0.041784 |
| rs538579 | -1.99553 | 1.028416 | 0.042082 |
| rs17105272 | -2.52948 | 1.304122 | 0.044157 |
| rs12905439 | -2.55046 | 1.315172 | 0.044258 |
| rs6056413 | 3.023543 | 1.460113 | 0.045021 |
| rs17720922 | -2.67251 | 1.390623 | 0.046558 |
| rs1526665 | 2.563852 | 1.240864 | 0.046804 |
| rs17538472 | -2.73407 | 1.428858 | 0.047687 |
| **rsid** | **Beta** | **se** | **p (difference)^1^** |
| rs11739877 | 2.533089 | 1.2373 | 0.04896 |
| rs1277723 | -2.79585 | 1.471992 | 0.049517 |
| rs7209235 | 2.984838 | 1.489921 | 0.052574 |
| rs4936175 | 2.56416 | 1.272992 | 0.052603 |
| rs17056301 | 2.599977 | 1.293237 | 0.052925 |
| rs17094222 | 2.152788 | 1.065272 | 0.053634 |
| rs10198345 | 2.887837 | 1.464213 | 0.056625 |
| rs12629015 | -2.48591 | 1.362182 | 0.058107 |
| rs4757638 | 2.781073 | 1.420548 | 0.058809 |
| rs592483 | 1.955053 | 0.98812 | 0.060068 |
| rs2619976 | -2.48508 | 1.393016 | 0.063972 |
| rs2423668 | -2.16046 | 1.221261 | 0.064747 |
| rs13425435 | -2.29194 | 1.313186 | 0.069083 |
| rs11074446 | 1.94559 | 1.017771 | 0.069327 |
| rs1330052 | -1.86867 | 1.082625 | 0.069691 |
| rs10929925 | 1.913076 | 1.024424 | 0.076266 |
| rs6050446 | -1.94086 | 1.14938 | 0.07649 |
| rs3769948 | 2.313271 | 1.256015 | 0.077619 |
| rs9299 | -2.22815 | 1.326486 | 0.079844 |
| rs1158684 | 3.009186 | 1.668152 | 0.080814 |
| rs2281819 | -1.64103 | 0.996847 | 0.081579 |
| rs10842240 | 1.750332 | 0.94996 | 0.081795 |
| rs7685048 | -2.79779 | 1.665093 | 0.082282 |
| rs2161097 | 2.750787 | 1.532941 | 0.083382 |
| rs3813680 | -1.96071 | 1.189286 | 0.083859 |
| rs4835778 | -2.21204 | 1.339625 | 0.084997 |
| rs10497810 | 1.934294 | 1.071095 | 0.086271 |
| rs40245 | 2.296635 | 1.284823 | 0.086863 |
| rs2450444 | 2.39678 | 1.347082 | 0.087744 |
| rs1285245 | -1.93959 | 1.201655 | 0.090385 |
| rs7674623 | -2.08276 | 1.288248 | 0.090888 |
| rs4523610 | 2.397825 | 1.365314 | 0.091912 |
| rs7601895 | -1.39257 | 0.883927 | 0.092394 |
| rs10269783 | 1.833102 | 1.036833 | 0.094035 |
| rs33485 | -1.86935 | 1.179963 | 0.095917 |
| rs9388766 | 2.378582 | 1.38461 | 0.099346 |
| rs4523552 | 2.414952 | 1.410635 | 0.100296 |
| rs13110266 | 2.128596 | 1.238657 | 0.100934 |
| rs4911442 | -2.21164 | 1.407907 | 0.101291 |
| rs16871902 | 2.004883 | 1.168397 | 0.102456 |
| rs2035831 | -2.1802 | 1.396576 | 0.103226 |
| rs2744974 | 0.937944 | 0.518159 | 0.104921 |
| rs12888545 | -2.05892 | 1.332954 | 0.106057 |
| rs9989141 | 1.507817 | 0.874544 | 0.106717 |
| rs11129662 | 2.022116 | 1.195162 | 0.107194 |
| rs6850639 | -2.26245 | 1.470529 | 0.108841 |
| rs9848399 | -1.71316 | 1.132599 | 0.110332 |
| **rsid** | **Beta** | **se** | **p (difference)^1^** |
| rs17066842 | -0.86475 | 0.604316 | 0.112406 |
| rs459552 | 2.264694 | 1.368333 | 0.113099 |
| rs4718966 | -1.52303 | 1.021577 | 0.113186 |
| rs2600226 | -1.88292 | 1.250496 | 0.113654 |
| rs8047395 | -0.26218 | 0.225343 | 0.115807 |
| rs12964689 | -0.85682 | 0.609596 | 0.118576 |
| rs16867703 | -1.47817 | 1.010025 | 0.119294 |
| rs9514131 | 2.196075 | 1.34871 | 0.119567 |
| rs954018 | -1.85705 | 1.255885 | 0.120039 |
| rs1903579 | -1.61955 | 1.104315 | 0.120464 |
| rs2009416 | 1.893381 | 1.157531 | 0.12064 |
| rs13105058 | 2.190954 | 1.356305 | 0.122562 |
| rs3764835 | 2.130897 | 1.326404 | 0.125121 |
| rs7730898 | 1.384823 | 0.844029 | 0.127071 |
| rs9688431 | 1.955114 | 1.231006 | 0.131132 |
| rs12477088 | -1.45974 | 1.030143 | 0.131178 |
| rs9530843 | 1.938059 | 1.221514 | 0.131703 |
| rs12519652 | 2.388028 | 1.52027 | 0.131745 |
| rs13001304 | -1.98998 | 1.388661 | 0.133161 |
| rs17391694 | -0.76969 | 0.578128 | 0.134915 |
| rs3930349 | -1.89486 | 1.335081 | 0.136024 |
| rs1035010 | 1.760911 | 1.127096 | 0.139813 |
| rs16942944 | 2.265303 | 1.475243 | 0.141543 |
| rs3807875 | 2.318355 | 1.516208 | 0.142825 |
| rs7217226 | -1.76693 | 1.273603 | 0.143671 |
| rs6561766 | -1.73875 | 1.26043 | 0.145619 |
| rs896183 | 2.081654 | 1.364601 | 0.145765 |
| rs10920678 | -1.17435 | 0.873698 | 0.146228 |
| rs12589208 | -2.02796 | 1.464532 | 0.14708 |
| rs6545714 | 1.109923 | 0.699973 | 0.147947 |
| rs7206608 | -1.44805 | 1.073176 | 0.150403 |
| rs7235205 | -1.65286 | 1.218234 | 0.151268 |
| rs769674 | 1.790848 | 1.182406 | 0.151919 |
| rs12429545 | -0.74084 | 0.584734 | 0.153032 |
| rs11246991 | 2.096246 | 1.41312 | 0.157051 |
| rs12380502 | 2.342071 | 1.587831 | 0.157299 |
| rs17757975 | 1.939289 | 1.303512 | 0.157479 |
| rs7124681 | -0.62522 | 0.509572 | 0.157829 |
| rs1436344 | 1.449746 | 0.962256 | 0.159736 |
| rs10768994 | 1.67002 | 1.12207 | 0.160877 |
| rs12519552 | -1.79209 | 1.347092 | 0.161153 |
| rs4796243 | 2.077927 | 1.4147 | 0.161353 |
| rs9356132 | 2.108069 | 1.442611 | 0.163215 |
| rs17450772 | 1.666513 | 1.126189 | 0.163351 |
| rs1038088 | -1.70712 | 1.294604 | 0.163812 |
| rs1865989 | 2.23352 | 1.535123 | 0.163906 |
| rs2010281 | 1.261887 | 0.840272 | 0.165633 |
| **rsid** | **Beta** | **se** | **p (difference)^1^** |
| rs6023633 | -1.68319 | 1.288717 | 0.167537 |
| rs10438964 | 1.668838 | 1.140348 | 0.168007 |
| rs9318686 | 2.255251 | 1.575452 | 0.170616 |
| rs10939792 | 1.338328 | 0.906953 | 0.171058 |
| rs13159555 | -1.87419 | 1.44686 | 0.173398 |
| rs12454712 | -1.25321 | 0.997072 | 0.176228 |
| rs6561710 | 1.888019 | 1.325099 | 0.176405 |
| rs7535528 | 1.169292 | 0.796448 | 0.178184 |
| rs12705977 | -1.5072 | 1.191353 | 0.178557 |
| rs6713781 | -1.48476 | 1.175701 | 0.178941 |
| rs2124499 | 1.651081 | 1.157744 | 0.179399 |
| rs9478671 | 2.18649 | 1.557248 | 0.179566 |
| rs3736485 | 1.475767 | 1.030307 | 0.180753 |
| rs208015 | -0.86682 | 0.721583 | 0.182545 |
| rs6019482 | 1.552928 | 1.093815 | 0.183086 |
| rs1544459 | 1.868281 | 1.33491 | 0.184444 |
| rs4700646 | -1.59639 | 1.276503 | 0.185045 |
| rs1830074 | 1.81716 | 1.300092 | 0.1857 |
| rs213533 | -1.71652 | 1.374061 | 0.187256 |
| rs4740619 | 1.063588 | 0.733436 | 0.187558 |
| rs6662747 | 1.756505 | 1.264631 | 0.189336 |
| rs4430672 | 2.005742 | 1.456133 | 0.189809 |
| rs7534091 | -1.88747 | 1.521462 | 0.192446 |
| rs4860782 | 1.979472 | 1.444969 | 0.192543 |
| rs12602912 | 1.361564 | 0.97072 | 0.1926 |
| rs2832283 | 1.92968 | 1.409697 | 0.193475 |
| rs10887578 | -1.55095 | 1.267398 | 0.193923 |
| rs8123881 | -1.18308 | 0.98552 | 0.194572 |
| rs961917 | 1.804244 | 1.324337 | 0.197243 |
| rs10176705 | -1.48079 | 1.224401 | 0.197967 |
| rs2075650 | 1.328663 | 0.957341 | 0.198175 |
| rs2198679 | 1.79087 | 1.319665 | 0.199185 |
| rs17207196 | 0.922079 | 0.644437 | 0.200514 |
| rs10259786 | 1.867655 | 1.387515 | 0.201794 |
| rs7334078 | -1.30371 | 1.100624 | 0.203659 |
| rs13021737 | -0.32231 | 0.328378 | 0.204982 |
| rs6591407 | -1.7151 | 1.429094 | 0.205157 |
| rs1895957 | -0.99236 | 0.858996 | 0.205471 |
| rs2616192 | -1.53783 | 1.295542 | 0.207404 |
| rs2732275 | 1.894045 | 1.428806 | 0.208372 |
| rs10408013 | 1.870272 | 1.41248 | 0.209203 |
| rs8036040 | -1.60792 | 1.357649 | 0.209586 |
| rs4740383 | -1.26085 | 1.08826 | 0.212668 |
| rs9077 | -1.10736 | 0.970612 | 0.215306 |
| rs16943356 | -1.61997 | 1.391827 | 0.217738 |
| rs2317299 | 1.584531 | 1.208722 | 0.218327 |
| rs12759296 | 2.059 | 1.594404 | 0.218366 |
| **rsid** | **Beta** | **se** | **p (difference)^1^** |
| rs4012234 | 1.282569 | 0.964828 | 0.219054 |
| rs987237 | -0.44472 | 0.439587 | 0.219957 |
| rs4745794 | 1.878369 | 1.458252 | 0.221739 |
| rs11855853 | -1.14463 | 1.019699 | 0.223964 |
| rs3766430 | 1.733053 | 1.346012 | 0.224053 |
| rs10846428 | 2.262776 | 1.782332 | 0.22419 |
| rs2682406 | 1.311974 | 1.004127 | 0.226176 |
| rs2012502 | 1.384102 | 1.064235 | 0.226385 |
| rs17806379 | -0.90935 | 0.832227 | 0.227392 |
| rs12779328 | 1.913977 | 1.508849 | 0.228375 |
| rs13240600 | 1.16073 | 0.889083 | 0.231434 |
| rs159032 | -1.2848 | 1.156105 | 0.232518 |
| rs591088 | -1.54895 | 1.380851 | 0.233677 |
| rs779206 | -1.49024 | 1.334279 | 0.234637 |
| rs11096549 | 2.000212 | 1.610848 | 0.23727 |
| rs3849570 | 1.345276 | 1.058883 | 0.238326 |
| rs2931434 | -1.69672 | 1.521344 | 0.238746 |
| rs1158805 | 1.353665 | 1.070866 | 0.240463 |
| rs12448257 | -0.88839 | 0.840012 | 0.241596 |
| rs676749 | -1.64622 | 1.49159 | 0.242902 |
| rs7652415 | 1.84734 | 1.500897 | 0.243393 |
| rs2448241 | 1.669191 | 1.353208 | 0.245162 |
| rs12468863 | 1.095197 | 0.862142 | 0.24684 |
| rs7805441 | 1.365576 | 1.096425 | 0.247129 |
| rs7134628 | 1.606485 | 1.304833 | 0.247192 |
| rs1707322 | 1.481151 | 1.199565 | 0.248405 |
| rs427943 | -0.9712 | 0.924594 | 0.248691 |
| rs2074881 | 1.613877 | 1.318742 | 0.249895 |
| rs6767619 | -1.44774 | 1.342089 | 0.250172 |
| rs6852276 | 1.914472 | 1.583427 | 0.250903 |
| rs2543132 | 1.469722 | 1.196205 | 0.251001 |
| rs6661316 | -1.49413 | 1.391872 | 0.253394 |
| rs1154659 | -1.57558 | 1.463862 | 0.253607 |
| rs1263618 | 1.553087 | 1.275966 | 0.253651 |
| rs811054 | 1.316413 | 1.068741 | 0.253735 |
| rs12149756 | 1.62159 | 1.338341 | 0.254483 |
| rs17535749 | 1.963146 | 1.638359 | 0.254544 |
| rs10811901 | -1.34433 | 1.270313 | 0.257013 |
| rs11505821 | 1.379515 | 1.134213 | 0.258009 |
| rs10878946 | -1.19419 | 1.140487 | 0.25813 |
| rs17272434 | -1.43251 | 1.357779 | 0.260406 |
| rs17695092 | -1.64183 | 1.547384 | 0.261504 |
| rs13432055 | -1.38553 | 1.320479 | 0.262015 |
| rs1829130 | 1.582904 | 1.335537 | 0.265726 |
| rs3781099 | 1.474685 | 1.238888 | 0.265964 |
| rs12776880 | -1.22482 | 1.190334 | 0.267334 |
| rs7512146 | 1.522849 | 1.28623 | 0.267464 |
| **rsid** | **Beta** | **se** | **p (difference)^1^** |
| rs3209570 | -1.05787 | 1.040805 | 0.267818 |
| rs6548834 | 1.338162 | 1.12214 | 0.268539 |
| rs577525 | 1.109515 | 0.919696 | 0.270791 |
| rs2489676 | -1.39307 | 1.355369 | 0.272055 |
| rs3844598 | 1.597273 | 1.368964 | 0.27295 |
| rs11738695 | 1.864411 | 1.616671 | 0.27413 |
| rs9814633 | 1.372846 | 1.169421 | 0.275105 |
| rs1496742 | 1.662584 | 1.436372 | 0.275568 |
| rs4711986 | -0.92112 | 0.93702 | 0.277992 |
| rs1485038 | -1.1708 | 1.169956 | 0.279085 |
| rs7950748 | -1.30594 | 1.295878 | 0.279464 |
| rs11904490 | -1.39416 | 1.378159 | 0.279703 |
| rs11121210 | 1.559493 | 1.357037 | 0.280993 |
| rs16982345 | 1.734048 | 1.519131 | 0.281037 |
| rs10481754 | 1.639829 | 1.433249 | 0.281555 |
| rs11170468 | -1.30299 | 1.300369 | 0.282142 |
| rs9814557 | 1.512353 | 1.32083 | 0.283744 |
| rs17636031 | 1.129549 | 0.963723 | 0.283823 |
| rs1227244 | -1.46583 | 1.457835 | 0.28413 |
| rs12147845 | -1.18287 | 1.2002 | 0.286796 |
| rs460799 | -1.12558 | 1.153601 | 0.289817 |
| rs9827823 | 1.154651 | 1.000052 | 0.290071 |
| rs2143624 | 1.787174 | 1.599842 | 0.290588 |
| rs1199334 | -1.16952 | 1.197799 | 0.290891 |
| rs1336486 | -0.93008 | 0.971033 | 0.290918 |
| rs4856794 | 1.624716 | 1.452352 | 0.292674 |
| rs2837398 | 1.35196 | 1.194359 | 0.293198 |
| rs175165 | -1.46394 | 1.487058 | 0.294282 |
| rs11138082 | -1.48503 | 1.511235 | 0.295581 |
| rs2269828 | -1.36377 | 1.396346 | 0.295951 |
| rs936227 | -1.2205 | 1.259832 | 0.296185 |
| rs2809395 | -1.27599 | 1.314007 | 0.296565 |
| rs11945861 | 1.426152 | 1.276691 | 0.29765 |
| rs2477017 | -1.43236 | 1.468786 | 0.298191 |
| rs1657930 | 1.729576 | 1.576656 | 0.300275 |
| rs1275691 | 1.748898 | 1.596577 | 0.300657 |
| rs6710871 | 1.18851 | 1.056701 | 0.301448 |
| rs10904675 | -1.35508 | 1.404923 | 0.301792 |
| rs7780752 | -0.99005 | 1.054773 | 0.303395 |
| rs298563 | -1.47215 | 1.526552 | 0.304403 |
| rs9361779 | -1.49225 | 1.547226 | 0.304747 |
| rs17201143 | 1.65412 | 1.521203 | 0.305838 |
| rs903959 | -1.21697 | 1.28624 | 0.307503 |
| rs4858887 | 1.286468 | 1.169949 | 0.309111 |
| rs13209872 | 1.203881 | 1.094383 | 0.311621 |
| rs7144011 | 0.669473 | 0.567539 | 0.313145 |
| rs2371767 | 1.566436 | 1.462608 | 0.314867 |
| **rsid** | **Beta** | **se** | **p (difference)^1^** |
| rs2440452 | 1.563338 | 1.465618 | 0.316882 |
| rs11119208 | 1.766572 | 1.669546 | 0.317122 |
| rs1402025 | 1.434128 | 1.338295 | 0.317536 |
| rs12868881 | 1.174173 | 1.079426 | 0.318122 |
| rs6898812 | 1.539252 | 1.447037 | 0.31872 |
| rs16846136 | 1.446369 | 1.355139 | 0.319178 |
| rs3810291 | -0.47348 | 0.570836 | 0.319248 |
| rs349088 | -0.92404 | 1.027025 | 0.320846 |
| rs189843 | 1.623994 | 1.543159 | 0.322216 |
| rs7929418 | -1.125 | 1.234813 | 0.322913 |
| rs10211055 | 1.035566 | 0.950885 | 0.323424 |
| rs9318380 | -1.17938 | 1.298409 | 0.326112 |
| rs10923724 | -1.1789 | 1.297967 | 0.326132 |
| rs13329567 | -0.45026 | 0.558307 | 0.328687 |
| rs7488867 | 0.832925 | 0.753704 | 0.328704 |
| rs962796 | -1.02064 | 1.143478 | 0.32899 |
| rs11889536 | -0.78266 | 0.899919 | 0.329186 |
| rs1477199 | -0.76203 | 0.878807 | 0.329209 |
| rs4538727 | -1.45741 | 1.597872 | 0.331055 |
| rs1420341 | 1.483762 | 1.429687 | 0.331856 |
| rs6595205 | 1.204368 | 1.144547 | 0.333073 |
| rs6587552 | 1.00018 | 0.933733 | 0.3332 |
| rs6564360 | 1.288118 | 1.232495 | 0.333618 |
| rs6921533 | -1.11455 | 1.252108 | 0.333793 |
| rs7550711 | 0.728427 | 0.65539 | 0.335219 |
| rs10263780 | 1.321105 | 1.271938 | 0.33564 |
| rs9904177 | -1.19793 | 1.345656 | 0.336401 |
| rs17513613 | -0.68634 | 0.814293 | 0.337052 |
| rs2733287 | -0.7457 | 0.876543 | 0.337253 |
| rs889398 | 0.753369 | 0.684229 | 0.337284 |
| rs12595158 | 1.575288 | 1.544477 | 0.338294 |
| rs1964927 | -0.92677 | 1.071357 | 0.339967 |
| rs1150659 | -0.99078 | 1.139958 | 0.340597 |
| rs1596299 | 1.479442 | 1.454402 | 0.341639 |
| rs17446091 | -1.24364 | 1.411804 | 0.342796 |
| rs6014523 | -0.95557 | 1.108618 | 0.343051 |
| rs13063194 | -1.07463 | 1.235755 | 0.343644 |
| rs1899898 | 1.525006 | 1.510028 | 0.344105 |
| rs7239114 | -1.01162 | 1.176052 | 0.346464 |
| rs420158 | -1.09181 | 1.261426 | 0.346527 |
| rs6574695 | -1.3116 | 1.497708 | 0.347406 |
| rs3902951 | 1.279002 | 1.265453 | 0.350052 |
| rs6448587 | 1.059656 | 1.032493 | 0.350922 |
| rs2155645 | -1.26353 | 1.464425 | 0.353323 |
| rs249292 | 1.492416 | 1.506152 | 0.353985 |
| rs9294260 | 1.04877 | 1.027689 | 0.354148 |
| rs7195386 | 0.965146 | 0.937488 | 0.354201 |
| **rsid** | **Beta** | **se** | **p (difference)^1^** |
| rs2065418 | 1.014054 | 0.99039 | 0.354241 |
| rs2875762 | 0.98442 | 0.959379 | 0.354739 |
| rs4482463 | -0.5999 | 0.753455 | 0.35613 |
| rs10009336 | 1.288748 | 1.294733 | 0.357106 |
| rs1006353 | -1.15496 | 1.358244 | 0.357173 |
| rs9965170 | 1.44595 | 1.466825 | 0.357544 |
| rs11577094 | -1.13364 | 1.342751 | 0.359924 |
| rs9397927 | 1.422977 | 1.456848 | 0.362513 |
| rs765875 | 1.335472 | 1.364373 | 0.363797 |
| rs1523768 | 1.246836 | 1.26768 | 0.364152 |
| rs621042 | -0.98201 | 1.189574 | 0.364993 |
| rs1467693 | -0.89041 | 1.092304 | 0.3667 |
| rs7925214 | 0.971151 | 0.97549 | 0.369946 |
| rs559231 | 1.008209 | 1.017693 | 0.370345 |
| rs1814170 | -1.05083 | 1.280895 | 0.370752 |
| rs879620 | -0.36889 | 0.518575 | 0.370936 |
| rs4307239 | -0.83896 | 1.048299 | 0.372677 |
| rs10886017 | 1.077731 | 1.100988 | 0.372803 |
| rs10733051 | -1.17169 | 1.428686 | 0.375026 |
| rs12189178 | -0.96759 | 1.209408 | 0.379327 |
| rs6121381 | 1.17418 | 1.227425 | 0.379916 |
| rs11251352 | 1.177894 | 1.237835 | 0.382298 |
| rs3007105 | 0.93499 | 0.960487 | 0.382698 |
| rs755407 | -1.03565 | 1.297645 | 0.383303 |
| rs4759075 | -1.08877 | 1.37054 | 0.38746 |
| rs1941697 | 1.09225 | 1.153545 | 0.388003 |
| rs653264 | 1.309279 | 1.408249 | 0.389083 |
| rs9527455 | 1.291777 | 1.389721 | 0.389697 |
| rs7573263 | 0.878651 | 0.910295 | 0.390255 |
| rs12885454 | -0.54277 | 0.74515 | 0.391753 |
| rs2195086 | 1.31008 | 1.419568 | 0.392562 |
| rs10243319 | -0.98968 | 1.277326 | 0.395487 |
| rs8192675 | -0.82503 | 1.083504 | 0.3955 |
| rs6471941 | -0.92087 | 1.198268 | 0.396257 |
| rs2718786 | 1.175583 | 1.275118 | 0.397368 |
| rs236527 | 1.322271 | 1.450139 | 0.397904 |
| rs17820822 | 0.890805 | 0.940006 | 0.398135 |
| rs12922346 | -0.73367 | 0.98289 | 0.398852 |
| rs9921416 | -1.12606 | 1.458681 | 0.402259 |
| rs10883759 | 1.224689 | 1.355556 | 0.405207 |
| rs1361739 | -0.58413 | 0.817796 | 0.405957 |
| rs9806058 | -0.98958 | 1.306005 | 0.40598 |
| rs7599312 | -0.71269 | 0.97463 | 0.406933 |
| rs2470520 | 1.095262 | 1.209704 | 0.40898 |
| rs4889606 | -0.47884 | 0.696383 | 0.409609 |
| rs1465900 | 1.268812 | 1.423713 | 0.410215 |
| rs1345942 | -0.9178 | 1.235998 | 0.412235 |
| **rsid** | **Beta** | **se** | **p (difference)^1^** |
| rs4670627 | -0.96444 | 1.294393 | 0.412776 |
| rs17789218 | 1.129321 | 1.265457 | 0.414361 |
| rs12206564 | 1.039503 | 1.156276 | 0.414724 |
| rs1584121 | -1.06242 | 1.424321 | 0.416148 |
| rs12449219 | 1.152074 | 1.299878 | 0.41671 |
| rs8070454 | -1.21901 | 1.619036 | 0.416749 |
| rs536445 | 0.951097 | 1.052827 | 0.416938 |
| rs2143253 | -0.85872 | 1.181949 | 0.419396 |
| rs7172627 | -0.81101 | 1.125761 | 0.420603 |
| rs7715256 | -0.52844 | 0.775123 | 0.420869 |
| rs2174307 | 1.035309 | 1.168227 | 0.42158 |
| rs2282802 | -0.94112 | 1.291956 | 0.422255 |
| rs7826312 | 1.107626 | 1.260759 | 0.422506 |
| rs4077093 | 1.230371 | 1.414336 | 0.422669 |
| rs10499275 | -0.89612 | 1.239281 | 0.423533 |
| rs4717623 | -0.84736 | 1.179725 | 0.424082 |
| rs7313220 | -0.98993 | 1.358422 | 0.424178 |
| rs2270778 | 1.354304 | 1.576836 | 0.424994 |
| rs1852006 | 0.878308 | 0.980454 | 0.425219 |
| rs11736228 | 1.024639 | 1.165144 | 0.425652 |
| rs1524277 | -1.00767 | 1.385206 | 0.42571 |
| rs6445538 | 0.873786 | 0.976635 | 0.426097 |
| rs10832778 | 0.953999 | 1.081227 | 0.427704 |
| rs1928295 | -0.68887 | 0.989932 | 0.428101 |
| rs7871866 | 0.922606 | 1.043531 | 0.428547 |
| rs17424278 | 1.332809 | 1.56458 | 0.429353 |
| rs1187352 | -0.81778 | 1.156314 | 0.429552 |
| rs11165643 | -0.41673 | 0.65438 | 0.433865 |
| rs486359 | -0.86173 | 1.223743 | 0.433996 |
| rs9644681 | 1.084909 | 1.264666 | 0.434422 |
| rs2063177 | 1.062158 | 1.237779 | 0.435249 |
| rs13184896 | -0.73973 | 1.070749 | 0.435288 |
| rs6807940 | 1.170966 | 1.378667 | 0.435711 |
| rs849135 | -0.88487 | 1.258069 | 0.43573 |
| rs11708733 | 1.306823 | 1.553151 | 0.435753 |
| rs6545709 | -0.8066 | 1.160646 | 0.436929 |
| rs11577179 | -0.88209 | 1.262749 | 0.43873 |
| rs995258 | 0.808636 | 0.924618 | 0.441187 |
| rs4858193 | -0.68327 | 1.012485 | 0.441722 |
| rs10118866 | -0.80586 | 1.1754 | 0.443079 |
| rs573455 | -1.07129 | 1.52383 | 0.443753 |
| rs4936671 | 1.060198 | 1.261171 | 0.444733 |
| rs12454204 | 0.844935 | 0.984835 | 0.44726 |
| rs11079849 | 0.680938 | 0.770835 | 0.448407 |
| rs11635675 | -0.77891 | 1.157285 | 0.449812 |
| rs16833232 | -1.09781 | 1.582032 | 0.450569 |
| rs7607369 | 0.944915 | 1.126762 | 0.451423 |
| **rsid** | **Beta** | **se** | **p (difference)^1^** |
| rs10131890 | 1.357269 | 1.683274 | 0.453783 |
| rs1927790 | -0.64178 | 0.985131 | 0.454142 |
| rs1951455 | -0.71242 | 1.086587 | 0.457063 |
| rs2396625 | -0.55681 | 0.877593 | 0.45725 |
| rs6691857 | -0.83068 | 1.249162 | 0.458324 |
| rs3807566 | 1.010602 | 1.23502 | 0.459147 |
| rs2717926 | 1.032333 | 1.267807 | 0.460358 |
| rs3829849 | -0.93731 | 1.400725 | 0.460807 |
| rs1668633 | 1.092462 | 1.350749 | 0.460846 |
| rs13417156 | -0.59363 | 0.942655 | 0.464675 |
| rs9951893 | -0.90442 | 1.375359 | 0.4671 |
| rs833831 | 1.098252 | 1.378209 | 0.467244 |
| rs491711 | -0.77376 | 1.19661 | 0.467466 |
| rs2605603 | -1.01645 | 1.539712 | 0.470069 |
| rs2174367 | -0.86002 | 1.324616 | 0.47058 |
| rs2119753 | 1.112364 | 1.410832 | 0.47142 |
| rs4820408 | -0.59412 | 0.957773 | 0.47143 |
| rs9458814 | -0.89744 | 1.380719 | 0.471939 |
| rs10975933 | 0.96219 | 1.206305 | 0.472915 |
| rs29941 | -0.65896 | 1.052076 | 0.473205 |
| rs6235 | 0.715782 | 0.865699 | 0.47439 |
| rs6985109 | 0.654989 | 0.783763 | 0.476146 |
| rs12762034 | -0.70958 | 1.133119 | 0.477286 |
| rs802460 | -0.9097 | 1.432531 | 0.482752 |
| rs7254892 | 1.119695 | 1.459035 | 0.483046 |
| rs4556997 | 0.717835 | 0.885885 | 0.483058 |
| rs12953970 | 1.001671 | 1.29092 | 0.483113 |
| rs7220138 | -0.70702 | 1.146368 | 0.483784 |
| rs355777 | -0.53061 | 0.89625 | 0.484729 |
| rs9650755 | -0.62385 | 1.031603 | 0.485502 |
| rs12439798 | -0.59139 | 0.987956 | 0.486796 |
| rs243387 | 1.123817 | 1.478062 | 0.486945 |
| rs7802342 | 0.973017 | 1.262135 | 0.487311 |
| rs11866815 | -0.59405 | 0.995264 | 0.488314 |
| rs1021066 | -0.91171 | 1.455096 | 0.488694 |
| rs10838465 | 0.784789 | 0.999242 | 0.490895 |
| rs12597712 | -0.85269 | 1.37904 | 0.4916 |
| rs7615297 | -0.85788 | 1.388426 | 0.492174 |
| rs331949 | -0.86203 | 1.397357 | 0.493069 |
| rs2365389 | 0.635406 | 0.786715 | 0.493359 |
| rs7009017 | -0.84252 | 1.370577 | 0.493603 |
| rs2362777 | -0.80691 | 1.318966 | 0.493738 |
| rs323742 | 0.771858 | 0.987112 | 0.493818 |
| rs5751239 | -0.80483 | 1.318391 | 0.494549 |
| rs12044597 | -0.58746 | 1.001277 | 0.49508 |
| rs33436 | -0.60806 | 1.032983 | 0.495701 |
| rs7607351 | 0.912013 | 1.199214 | 0.496404 |
| **rsid** | **Beta** | **se** | **p (difference)^1^** |
| rs8089514 | 0.937124 | 1.237371 | 0.49683 |
| rs4148155 | 0.894808 | 1.183107 | 0.499755 |
| rs6477694 | 0.897955 | 1.19379 | 0.501918 |
| rs1000940 | 0.71243 | 0.918354 | 0.502382 |
| rs16916303 | 1.079503 | 1.4669 | 0.502691 |
| rs733594 | -0.67174 | 1.151537 | 0.505111 |
| rs7551507 | -0.44165 | 0.806797 | 0.505484 |
| rs3819811 | -0.85987 | 1.449099 | 0.509585 |
| rs1512914 | 0.783697 | 1.046781 | 0.511444 |
| rs998732 | 0.780637 | 1.043872 | 0.512153 |
| rs7779498 | 1.062531 | 1.481272 | 0.514203 |
| rs2866816 | -0.73459 | 1.276488 | 0.515373 |
| rs12628891 | 1.039824 | 1.451651 | 0.51571 |
| rs340025 | 0.888265 | 1.221511 | 0.516779 |
| rs9296723 | 1.003666 | 1.401895 | 0.517474 |
| rs17100323 | 1.082214 | 1.524354 | 0.517768 |
| rs11880870 | -0.43915 | 0.827085 | 0.517919 |
| rs10757826 | 0.902172 | 1.24963 | 0.519014 |
| rs7761673 | 0.969997 | 1.357728 | 0.519903 |
| rs2322622 | -0.92514 | 1.590186 | 0.520851 |
| rs10192119 | 0.881537 | 1.224828 | 0.521475 |
| rs2467210 | -0.63717 | 1.144468 | 0.52192 |
| rs2233984 | -0.87348 | 1.523987 | 0.524761 |
| rs10824218 | 0.737403 | 1.008806 | 0.525157 |
| rs7748777 | -0.81473 | 1.433125 | 0.525206 |
| rs6879326 | 0.972304 | 1.380435 | 0.525698 |
| rs6504165 | -0.62591 | 1.14392 | 0.528137 |
| rs7630080 | 1.067644 | 1.543955 | 0.529252 |
| rs4663629 | 0.921611 | 1.316192 | 0.530633 |
| rs254800 | -0.85073 | 1.517371 | 0.532757 |
| rs2429150 | -0.64154 | 1.184532 | 0.533656 |
| rs11915371 | -0.65318 | 1.20438 | 0.534042 |
| rs10803762 | 0.839144 | 1.195043 | 0.534219 |
| rs7117238 | 0.943989 | 1.365109 | 0.534618 |
| rs3814883 | -0.27433 | 0.597468 | 0.535948 |
| rs13266989 | 0.854524 | 1.236446 | 0.539734 |
| rs10840606 | -0.53526 | 1.034436 | 0.541884 |
| rs12299814 | 0.671768 | 0.947877 | 0.543844 |
| rs8088123 | -0.51176 | 1.001256 | 0.544043 |
| rs7578575 | 0.805674 | 1.17096 | 0.544661 |
| rs965961 | -0.5861 | 1.125605 | 0.544674 |
| rs4483850 | -0.40301 | 0.827093 | 0.546579 |
| rs7133378 | 0.843859 | 1.242094 | 0.547278 |
| rs13201877 | -0.71583 | 1.354213 | 0.548951 |
| rs4521182 | -0.75161 | 1.415428 | 0.549374 |
| rs11611246 | 0.521231 | 0.712464 | 0.551085 |
| rs11754747 | -0.73755 | 1.398315 | 0.551196 |
| **rsid** | **Beta** | **se** | **p (difference)^1^** |
| rs294704 | -0.67385 | 1.296908 | 0.552885 |
| rs12148330 | -0.69627 | 1.335903 | 0.553244 |
| rs1899689 | 0.834412 | 1.246167 | 0.553649 |
| rs11075489 | 0.790079 | 1.172674 | 0.554117 |
| rs4653017 | 0.985257 | 1.505132 | 0.554759 |
| rs12989476 | 0.777598 | 1.155196 | 0.555361 |
| rs4372836 | 0.747796 | 1.104862 | 0.55544 |
| rs17387761 | -0.70325 | 1.360164 | 0.556887 |
| rs4929923 | 0.566862 | 0.80312 | 0.558054 |
| rs2282231 | 0.686544 | 1.013044 | 0.560173 |
| rs1853639 | 0.801489 | 1.214204 | 0.561391 |
| rs6497676 | -0.85692 | 1.641002 | 0.561508 |
| rs12615778 | 0.861536 | 1.323474 | 0.56312 |
| rs4516268 | -0.38332 | 0.831014 | 0.564357 |
| rs17724992 | -0.4013 | 0.864572 | 0.565409 |
| rs852056 | -0.62031 | 1.246757 | 0.565717 |
| rs1521527 | -0.56639 | 1.156777 | 0.567039 |
| rs657452 | 0.505872 | 0.715429 | 0.56717 |
| rs12904761 | 0.912907 | 1.433394 | 0.568862 |
| rs2029331 | -0.53045 | 1.099857 | 0.569119 |
| rs329651 | -0.51183 | 1.070069 | 0.570173 |
| rs12422552 | -0.47801 | 1.011741 | 0.57066 |
| rs11792311 | -0.53413 | 1.113446 | 0.571587 |
| rs13191362 | 0.645953 | 0.972676 | 0.572055 |
| rs10058464 | 0.712979 | 1.091422 | 0.572076 |
| rs12443621 | -0.71849 | 1.443543 | 0.572679 |
| rs6712 | -0.75932 | 1.519406 | 0.573554 |
| rs11066188 | -0.53946 | 1.128819 | 0.573615 |
| rs12369179 | 0.424844 | 0.583369 | 0.573633 |
| rs535533 | 0.905262 | 1.439436 | 0.574097 |
| rs1784460 | -0.46268 | 1.011379 | 0.580856 |
| rs273504 | 0.606434 | 0.924493 | 0.58114 |
| rs8067737 | 1.027373 | 1.691488 | 0.581982 |
| rs1007934 | -0.56418 | 1.202044 | 0.582979 |
| rs12939549 | 0.513632 | 0.761908 | 0.583994 |
| rs9475173 | -0.63075 | 1.332519 | 0.585576 |
| rs2246012 | -0.60507 | 1.287198 | 0.586098 |
| rs7730004 | 0.606521 | 0.943832 | 0.588838 |
| rs12325419 | -0.64715 | 1.380129 | 0.590342 |
| rs6606686 | 0.651208 | 1.03687 | 0.592547 |
| rs1304070 | -0.49547 | 1.105806 | 0.592879 |
| rs4704513 | -0.59149 | 1.286467 | 0.593164 |
| rs1452075 | -0.5047 | 1.125882 | 0.593796 |
| rs10145461 | 0.648385 | 1.035452 | 0.593926 |
| rs845084 | -0.54862 | 1.213539 | 0.595403 |
| rs4783241 | -0.58358 | 1.279981 | 0.595571 |
| rs12439829 | -0.55892 | 1.235807 | 0.596253 |
| **rsid** | **Beta** | **se** | **p (difference)^1^** |
| rs2163188 | 0.676066 | 1.097198 | 0.597224 |
| rs1911746 | 0.838267 | 1.406925 | 0.597914 |
| rs16906845 | 0.796402 | 1.332388 | 0.599251 |
| rs8061382 | 0.701186 | 1.152482 | 0.599681 |
| rs10797115 | -0.43372 | 1.0112 | 0.600552 |
| rs6841761 | 0.620057 | 1.00047 | 0.600641 |
| rs10795422 | -0.39943 | 0.947409 | 0.601215 |
| rs6930066 | -0.62856 | 1.386928 | 0.601459 |
| rs818524 | 0.866932 | 1.47588 | 0.601537 |
| rs3811514 | -0.45506 | 1.060131 | 0.603352 |
| rs6265 | -0.12562 | 0.427173 | 0.604918 |
| rs10514222 | 0.889095 | 1.534028 | 0.605259 |
| rs1014194 | 0.766152 | 1.298063 | 0.605807 |
| rs4906908 | 0.798356 | 1.361486 | 0.606071 |
| rs2190788 | -0.43416 | 1.027752 | 0.60612 |
| rs17203016 | -0.51296 | 1.185182 | 0.607501 |
| rs16965225 | -0.59628 | 1.347521 | 0.607516 |
| rs6759670 | 0.862634 | 1.49979 | 0.609347 |
| rs774211 | 0.831857 | 1.440484 | 0.609579 |
| rs11649864 | 0.787509 | 1.3604 | 0.611362 |
| rs17236194 | 0.786014 | 1.360826 | 0.612244 |
| rs17327461 | 0.617845 | 1.028936 | 0.612251 |
| rs6512302 | 0.767812 | 1.32575 | 0.612471 |
| rs17405819 | 0.426315 | 0.651919 | 0.612887 |
| rs7243357 | -0.42203 | 1.024522 | 0.613271 |
| rs1455137 | -0.56566 | 1.312953 | 0.61439 |
| rs12042959 | 0.947119 | 1.696941 | 0.616062 |
| rs4676084 | 0.857372 | 1.524066 | 0.617484 |
| rs7164727 | -0.285 | 0.762809 | 0.617744 |
| rs1511471 | -0.51871 | 1.250262 | 0.623054 |
| rs2467594 | -0.44403 | 1.099878 | 0.623566 |
| rs7519259 | 0.691665 | 1.220687 | 0.625721 |
| rs4864201 | 0.605088 | 1.044281 | 0.626108 |
| rs424539 | 0.741921 | 1.338427 | 0.62951 |
| rs2228213 | -0.37342 | 0.97473 | 0.630265 |
| rs10851523 | -0.58241 | 1.412964 | 0.631204 |
| rs11138313 | 0.719523 | 1.303226 | 0.632468 |
| rs2100814 | 0.658897 | 1.183458 | 0.634492 |
| rs17776719 | 0.642619 | 1.150652 | 0.634919 |
| rs2850969 | -0.43202 | 1.111777 | 0.634958 |
| rs7844647 | 0.848845 | 1.588959 | 0.635737 |
| rs12035149 | -0.45645 | 1.166742 | 0.635972 |
| rs8126575 | 0.8104 | 1.509128 | 0.63604 |
| rs11792069 | -0.42513 | 1.103171 | 0.636773 |
| rs4671328 | 0.420049 | 0.685938 | 0.63707 |
| rs2815324 | -0.52636 | 1.321225 | 0.637689 |
| rs2279620 | -0.52429 | 1.324172 | 0.639556 |
| **rsid** | **Beta** | **se** | **p (difference)^1^** |
| rs1358980 | -0.35254 | 0.959119 | 0.640196 |
| rs11089885 | 0.789615 | 1.484806 | 0.640503 |
| rs7784465 | 0.682112 | 1.256172 | 0.640938 |
| rs4880341 | 0.655863 | 1.202954 | 0.641793 |
| rs1399471 | 0.686525 | 1.271572 | 0.642496 |
| rs895330 | -0.27757 | 0.807822 | 0.644008 |
| rs1608445 | 0.705195 | 1.323628 | 0.645467 |
| rs7630302 | -0.37257 | 1.020545 | 0.646279 |
| rs4916229 | -0.45505 | 1.201936 | 0.646717 |
| rs4912637 | -0.56912 | 1.461681 | 0.649148 |
| rs1260326 | -0.59103 | 1.514778 | 0.65021 |
| rs217671 | -0.37737 | 1.046192 | 0.651065 |
| rs1730859 | -0.46046 | 1.230001 | 0.651073 |
| rs4237643 | 0.385153 | 0.642143 | 0.652982 |
| rs2271189 | -0.32601 | 0.938612 | 0.65316 |
| rs945211 | 0.664295 | 1.265822 | 0.653602 |
| rs12620249 | 0.727937 | 1.409124 | 0.653933 |
| rs9538141 | -0.25856 | 0.801926 | 0.658628 |
| rs483752 | -0.5338 | 1.433602 | 0.660501 |
| rs11781222 | -0.53953 | 1.449915 | 0.661213 |
| rs9489622 | -0.48892 | 1.339962 | 0.662532 |
| rs2289379 | 0.519055 | 0.969689 | 0.662853 |
| rs17767510 | 0.736979 | 1.469844 | 0.662876 |
| rs12593036 | 0.557215 | 1.087566 | 0.671681 |
| rs11128904 | 0.797471 | 1.655636 | 0.671875 |
| rs2162524 | -0.26616 | 0.854784 | 0.67199 |
| rs977747 | -0.27038 | 0.867338 | 0.672911 |
| rs10146527 | -0.3946 | 1.162076 | 0.672999 |
| rs7874154 | -0.33978 | 1.033488 | 0.673403 |
| rs1020548 | 0.756737 | 1.567372 | 0.673438 |
| rs7557796 | 0.451793 | 0.846956 | 0.674697 |
| rs1941213 | 0.64252 | 1.315102 | 0.677844 |
| rs12033257 | 0.481901 | 0.933034 | 0.679395 |
| rs10765208 | 0.712476 | 1.491837 | 0.679532 |
| rs761798 | 0.632047 | 1.306135 | 0.681629 |
| rs7987314 | -0.52018 | 1.504321 | 0.682148 |
| rs11672660 | -0.11582 | 0.518345 | 0.683351 |
| rs6738445 | -0.39597 | 1.210033 | 0.684408 |
| rs17531363 | -0.4133 | 1.254172 | 0.684762 |
| rs6556301 | 0.590214 | 1.220331 | 0.685626 |
| rs934515 | 0.581509 | 1.203927 | 0.686888 |
| rs17001561 | 0.598637 | 1.250806 | 0.687924 |
| rs17309825 | 0.533744 | 1.089585 | 0.688033 |
| rs1363695 | -0.43938 | 1.333327 | 0.688092 |
| rs472611 | 0.703179 | 1.513766 | 0.688435 |
| rs2160077 | 0.669503 | 1.437495 | 0.690023 |
| rs455527 | 0.644605 | 1.380683 | 0.691222 |
| **rsid** | **Beta** | **se** | **p (difference)^1^** |
| rs2172211 | 0.678332 | 1.469523 | 0.692 |
| rs7249143 | -0.43298 | 1.33654 | 0.692332 |
| rs10132280 | 0.369792 | 0.692594 | 0.692993 |
| rs6594967 | -0.53368 | 1.596408 | 0.693302 |
| rs3851083 | 0.665033 | 1.444385 | 0.693708 |
| rs17573940 | -0.39441 | 1.251033 | 0.69513 |
| rs4722398 | 0.607801 | 1.309847 | 0.696112 |
| rs4673553 | 0.456782 | 0.925374 | 0.696848 |
| rs9816226 | -0.09945 | 0.50326 | 0.698309 |
| rs2890652 | -0.30846 | 1.046914 | 0.699359 |
| rs10971721 | -0.35539 | 1.169311 | 0.699561 |
| rs4954638 | 0.616313 | 1.348397 | 0.6997 |
| rs16907751 | 0.617569 | 1.352478 | 0.699874 |
| rs9964756 | -0.40155 | 1.295923 | 0.701097 |
| rs7694732 | 0.582746 | 1.267914 | 0.701179 |
| rs13250058 | -0.43755 | 1.393705 | 0.701907 |
| rs5742914 | 0.545848 | 1.182025 | 0.703658 |
| rs6968554 | -0.48755 | 1.534267 | 0.703735 |
| rs999889 | -0.42479 | 1.380813 | 0.706112 |
| rs3803286 | -0.20834 | 0.807246 | 0.706361 |
| rs12630999 | -0.2284 | 0.863694 | 0.707391 |
| rs820071 | -0.30247 | 1.063591 | 0.708029 |
| rs2478879 | -0.23779 | 0.892575 | 0.708588 |
| rs2170382 | 0.560531 | 1.267638 | 0.714146 |
| rs1554790 | -0.30462 | 1.094433 | 0.714426 |
| rs10818810 | 0.487107 | 1.068582 | 0.714523 |
| rs9367368 | -0.36784 | 1.268967 | 0.714789 |
| rs2820311 | 0.325485 | 0.627771 | 0.715131 |
| rs17709991 | 0.667714 | 1.575962 | 0.716856 |
| rs1421334 | -0.35218 | 1.236582 | 0.717098 |
| rs215634 | 0.406924 | 0.861722 | 0.718474 |
| rs9507983 | 0.426399 | 0.915879 | 0.718503 |
| rs10071816 | -0.41163 | 1.410106 | 0.718902 |
| rs6819344 | -0.41661 | 1.45642 | 0.724909 |
| rs16932761 | -0.27196 | 1.050811 | 0.726316 |
| rs7561278 | -0.3335 | 1.234821 | 0.728041 |
| rs10883553 | 0.51317 | 1.202829 | 0.72885 |
| rs13012099 | 0.419361 | 0.938203 | 0.730547 |
| rs1045411 | 0.438397 | 0.998333 | 0.731797 |
| rs10491182 | 0.671805 | 1.682159 | 0.732194 |
| rs12446632 | -0.10871 | 0.601762 | 0.734054 |
| rs7025938 | 0.405069 | 0.912634 | 0.735073 |
| rs6781254 | 0.518126 | 1.247948 | 0.735286 |
| rs4677812 | 0.491315 | 1.169085 | 0.735388 |
| rs17448885 | 0.591634 | 1.474505 | 0.736855 |
| rs1048932 | -0.19857 | 0.877218 | 0.737165 |
| rs7636868 | 0.631983 | 1.613289 | 0.739789 |
| **rsid** | **Beta** | **se** | **p (difference)^1^** |
| rs7973955 | 0.518002 | 1.272532 | 0.740285 |
| rs10942267 | -0.23922 | 1.011035 | 0.740323 |
| rs2185027 | 0.483231 | 1.168795 | 0.740541 |
| rs11538 | 0.514094 | 1.262618 | 0.740657 |
| rs7323827 | 0.613252 | 1.562455 | 0.740683 |
| rs6764533 | 0.540342 | 1.345389 | 0.741296 |
| rs1955540 | 0.487781 | 1.188219 | 0.741736 |
| rs6482729 | 0.539577 | 1.349718 | 0.742524 |
| rs2080454 | -0.24172 | 1.029346 | 0.742943 |
| rs1787267 | 0.584991 | 1.497581 | 0.744113 |
| rs1876359 | -0.30041 | 1.223107 | 0.745926 |
| rs2836961 | 0.593492 | 1.547355 | 0.7479 |
| rs1320903 | -0.12665 | 0.693239 | 0.748314 |
| rs17681708 | 0.556234 | 1.443345 | 0.749915 |
| rs2479958 | -0.25587 | 1.103952 | 0.750005 |
| rs6011457 | -0.30648 | 1.271748 | 0.751695 |
| rs4886869 | 0.552016 | 1.448405 | 0.752968 |
| rs977540 | 0.451136 | 1.133638 | 0.754206 |
| rs1006896 | 0.379583 | 0.912424 | 0.756144 |
| rs10818938 | 0.450145 | 1.140932 | 0.756386 |
| rs10889550 | 0.618999 | 1.699799 | 0.758388 |
| rs1982441 | -0.21232 | 1.006271 | 0.759397 |
| rs11790280 | 0.518018 | 1.383945 | 0.760504 |
| rs4877313 | 0.504283 | 1.345965 | 0.761727 |
| rs223391 | 0.485301 | 1.30548 | 0.765645 |
| rs11056875 | 0.495367 | 1.340052 | 0.76578 |
| rs7181610 | 0.497309 | 1.354122 | 0.767047 |
| rs1625427 | 0.42518 | 1.114458 | 0.767839 |
| rs2293605 | -0.27868 | 1.270828 | 0.768173 |
| rs4969387 | -0.29151 | 1.322541 | 0.769563 |
| rs6548221 | 0.404242 | 1.053968 | 0.770078 |
| rs9349239 | -0.27591 | 1.273342 | 0.77028 |
| rs12651833 | -0.26632 | 1.242126 | 0.770575 |
| rs16851483 | 0.340257 | 0.836817 | 0.770587 |
| rs13227658 | 0.346025 | 0.86084 | 0.771682 |
| rs10962549 | 0.35809 | 0.902608 | 0.771721 |
| rs17014375 | 0.481485 | 1.337765 | 0.77332 |
| rs2023671 | 0.484077 | 1.363071 | 0.775957 |
| rs6804181 | -0.24427 | 1.2197 | 0.780312 |
| rs3005710 | -0.32811 | 1.524629 | 0.780908 |
| rs7181498 | 0.311846 | 0.792865 | 0.785667 |
| rs2959592 | -0.31236 | 1.504729 | 0.786127 |
| rs11702843 | 0.455309 | 1.324108 | 0.786205 |
| rs1554194 | -0.31511 | 1.521764 | 0.78707 |
| rs12150665 | 0.314659 | 0.809889 | 0.787387 |
| rs7640424 | -0.21552 | 1.156167 | 0.787648 |
| rs3957285 | -0.1722 | 0.99718 | 0.788043 |
| **rsid** | **Beta** | **se** | **p (difference)^1^** |
| rs2254542 | -0.27066 | 1.367184 | 0.788594 |
| rs8016771 | -0.26942 | 1.364948 | 0.788956 |
| rs13034320 | -0.34438 | 1.660941 | 0.790922 |
| rs1117080 | 0.486588 | 1.478276 | 0.791685 |
| rs1169091 | 0.422689 | 1.236604 | 0.79174 |
| rs11001963 | 0.46519 | 1.405592 | 0.792895 |
| rs9463175 | 0.479692 | 1.464029 | 0.793335 |
| rs12476772 | -0.19877 | 1.153945 | 0.798432 |
| rs11614340 | 0.387979 | 1.142937 | 0.798477 |
| rs13168288 | 0.426134 | 1.308184 | 0.800851 |
| rs663129 | 0.017026 | 0.312913 | 0.801736 |
| rs498240 | 0.332176 | 0.943959 | 0.80259 |
| rs11670142 | 0.42756 | 1.326351 | 0.802689 |
| rs756717 | -0.15313 | 0.997774 | 0.802903 |
| rs881301 | -0.27045 | 1.468673 | 0.802992 |
| rs7760082 | 0.384204 | 1.156024 | 0.803232 |
| rs7206395 | -0.24534 | 1.372972 | 0.803692 |
| rs3826705 | -0.28052 | 1.517892 | 0.804116 |
| rs10915840 | -0.25522 | 1.417834 | 0.804381 |
| rs6690764 | -0.20955 | 1.24655 | 0.806409 |
| rs6864049 | 0.380526 | 1.161134 | 0.806531 |
| rs10938397 | 0.203365 | 0.437063 | 0.806578 |
| rs6461115 | -0.19558 | 1.194616 | 0.807214 |
| rs4639527 | -0.12199 | 0.903324 | 0.809401 |
| rs11118308 | 0.448169 | 1.47348 | 0.811171 |
| rs40067 | -0.06257 | 0.666632 | 0.812161 |
| rs3845802 | 0.412387 | 1.331832 | 0.812306 |
| rs2516739 | 0.340915 | 1.034237 | 0.812933 |
| rs7147503 | -0.20149 | 1.27322 | 0.815292 |
| rs326889 | -0.11224 | 0.89376 | 0.815858 |
| rs3806116 | -0.23197 | 1.421272 | 0.817531 |
| rs7536433 | -0.16028 | 1.113609 | 0.81804 |
| rs273697 | -0.25886 | 1.545033 | 0.818363 |
| rs10163018 | 0.382632 | 1.250588 | 0.818805 |
| rs930295 | 0.293124 | 0.860819 | 0.819044 |
| rs10989568 | 0.378437 | 1.259892 | 0.822708 |
| rs7332115 | -0.12412 | 0.98581 | 0.823373 |
| rs1522569 | 0.330369 | 1.054816 | 0.824284 |
| rs284262 | 0.393615 | 1.357188 | 0.826501 |
| rs4865796 | 0.439194 | 1.569472 | 0.826968 |
| rs263041 | 0.371148 | 1.259878 | 0.827209 |
| rs7925748 | -0.14124 | 1.093036 | 0.828222 |
| rs2874800 | -0.19971 | 1.38436 | 0.830879 |
| rs223051 | 0.380531 | 1.332269 | 0.830961 |
| rs11951673 | -0.14595 | 1.149282 | 0.833301 |
| rs9845966 | -0.23181 | 1.584702 | 0.836136 |
| rs2527366 | 0.350733 | 1.241344 | 0.837495 |
| **rsid** | **Beta** | **se** | **p (difference)^1^** |
| rs7084454 | 0.253179 | 0.766415 | 0.837702 |
| rs12448738 | 0.327448 | 1.135709 | 0.838614 |
| rs1884897 | 0.237372 | 0.697701 | 0.839655 |
| rs3800229 | 0.296385 | 0.995768 | 0.840635 |
| rs2246664 | 0.31233 | 1.082933 | 0.84177 |
| rs10433609 | -0.1452 | 1.223372 | 0.843732 |
| rs11917965 | 0.343683 | 1.262805 | 0.844583 |
| rs7770443 | -0.15982 | 1.318819 | 0.846215 |
| rs13263601 | 0.311589 | 1.122637 | 0.847809 |
| rs248139 | 0.336829 | 1.259352 | 0.848422 |
| rs1241986 | -0.17221 | 1.409837 | 0.849138 |
| rs16882001 | -0.10859 | 1.07744 | 0.849439 |
| rs7567655 | 0.36489 | 1.41583 | 0.849439 |
| rs7235563 | 0.357795 | 1.38396 | 0.850032 |
| rs11659764 | 0.321271 | 1.1977 | 0.850894 |
| rs7024334 | -0.11463 | 1.14087 | 0.853556 |
| rs10135922 | 0.374184 | 1.508105 | 0.85371 |
| rs1075901 | 0.291038 | 1.070248 | 0.855498 |
| rs17619973 | 0.336645 | 1.329774 | 0.856463 |
| rs1885728 | -0.19536 | 1.613493 | 0.856711 |
| rs138289 | -0.12833 | 1.245079 | 0.857038 |
| rs17120344 | -0.11658 | 1.191025 | 0.858368 |
| rs155510 | -0.09742 | 1.087357 | 0.858848 |
| rs6707445 | -0.11203 | 1.172384 | 0.859192 |
| rs284227 | -0.09901 | 1.099683 | 0.859282 |
| rs8071182 | 0.324919 | 1.298101 | 0.860091 |
| rs2694047 | 0.256126 | 0.918679 | 0.861756 |
| rs329122 | -0.10217 | 1.139593 | 0.861974 |
| rs11150911 | -0.09599 | 1.109506 | 0.862653 |
| rs1863652 | 0.322115 | 1.325124 | 0.864577 |
| rs1296328 | -0.04541 | 0.835425 | 0.86565 |
| rs1445305 | -0.11416 | 1.28907 | 0.870512 |
| rs7498665 | 0.006793 | 0.549605 | 0.871234 |
| rs6901756 | 0.304157 | 1.283511 | 0.871235 |
| rs10197031 | 0.242357 | 0.906879 | 0.871913 |
| rs16903285 | 0.185077 | 0.55313 | 0.872351 |
| rs1937684 | 0.324141 | 1.420884 | 0.872496 |
| rs13153166 | 0.307075 | 1.32615 | 0.873605 |
| rs6047046 | -0.13425 | 1.448219 | 0.873687 |
| rs7565976 | -0.10713 | 1.290315 | 0.874923 |
| rs12041258 | 0.272242 | 1.122593 | 0.875333 |
| rs2357760 | -0.07008 | 1.061 | 0.875642 |
| rs4082793 | -0.05966 | 1.002562 | 0.876646 |
| rs825680 | 0.299565 | 1.347815 | 0.880011 |
| rs11173522 | 0.310234 | 1.420665 | 0.880191 |
| rs3887080 | 0.244212 | 1.001044 | 0.882395 |
| rs7941030 | -0.09459 | 1.295976 | 0.883094 |
| **rsid** | **Beta** | **se** | **p (difference)^1^** |
| rs3926926 | -0.09887 | 1.334244 | 0.883889 |
| rs11581304 | -0.07278 | 1.169686 | 0.885285 |
| rs13209968 | 0.262078 | 1.151224 | 0.885371 |
| rs9408882 | 0.347497 | 1.796317 | 0.888692 |
| rs10483389 | 0.238593 | 1.0199 | 0.888901 |
| rs1218822 | -0.02804 | 0.891386 | 0.889372 |
| rs10182181 | 0.152762 | 0.406553 | 0.889413 |
| rs719802 | 0.293859 | 1.437899 | 0.890607 |
| rs17019087 | 0.261789 | 1.206606 | 0.890783 |
| rs11604688 | 0.291162 | 1.424889 | 0.89111 |
| rs761423 | 0.275929 | 1.337981 | 0.893083 |
| rs8033995 | 0.282354 | 1.389677 | 0.893379 |
| rs946824 | 0.244961 | 1.110754 | 0.893395 |
| rs4722672 | 0.258996 | 1.217605 | 0.893575 |
| rs740157 | 0.263357 | 1.255172 | 0.893993 |
| rs9326846 | -0.07891 | 1.353382 | 0.897175 |
| rs6827083 | 0.27486 | 1.38367 | 0.897202 |
| rs16940823 | -0.05952 | 1.271278 | 0.902641 |
| rs6676084 | 0.25641 | 1.321377 | 0.903434 |
| rs6692586 | 0.230367 | 1.120229 | 0.904598 |
| rs7899106 | 0.218493 | 1.021635 | 0.904647 |
| rs1658820 | -0.03422 | 1.087534 | 0.904703 |
| rs10144318 | -0.06728 | 1.392568 | 0.906664 |
| rs12680842 | 0.209758 | 1.02323 | 0.911557 |
| rs10745785 | 0.231859 | 1.232151 | 0.912261 |
| rs7683836 | -0.04047 | 1.255693 | 0.913461 |
| rs1503526 | -0.01543 | 1.025653 | 0.913501 |
| rs7186893 | 0.20356 | 0.991925 | 0.913733 |
| rs4814512 | -0.04983 | 1.377271 | 0.915676 |
| rs13298062 | 0.242567 | 1.394313 | 0.91633 |
| rs4802778 | -0.02623 | 1.175354 | 0.917179 |
| rs4682718 | -0.02644 | 1.204958 | 0.919065 |
| rs13072095 | -0.0658 | 1.613372 | 0.920113 |
| rs702820 | -0.02862 | 1.257516 | 0.921062 |
| rs2051559 | 0.203546 | 1.088669 | 0.921375 |
| rs11496125 | 0.015919 | 0.825533 | 0.922744 |
| rs12101393 | 0.220826 | 1.290361 | 0.922987 |
| rs11191548 | 0.013459 | 0.871439 | 0.924554 |
| rs2192158 | 0.20617 | 1.187157 | 0.926119 |
| rs10830452 | 0.205631 | 1.182502 | 0.926191 |
| rs2274550 | -0.03115 | 1.4222 | 0.928755 |
| rs4953577 | 0.220311 | 1.400525 | 0.929317 |
| rs10942476 | 0.220389 | 1.408199 | 0.929657 |
| rs4986044 | 0.171343 | 0.860557 | 0.930325 |
| rs7869771 | -0.0061 | 1.184287 | 0.931295 |
| rs901630 | 0.005197 | 1.072505 | 0.932529 |
| rs17599948 | -0.02983 | 1.541494 | 0.934937 |
| **rsid** | **Beta** | **se** | **p (difference)^1^** |
| rs1993709 | 0.132313 | 0.449582 | 0.935875 |
| rs1476322 | 0.206471 | 1.385307 | 0.936483 |
| rs10779751 | 0.189042 | 1.171846 | 0.936771 |
| rs857601 | -0.01735 | 1.440117 | 0.937255 |
| rs13292976 | 0.167453 | 0.963557 | 0.940957 |
| rs2731277 | 0.191382 | 1.29452 | 0.941309 |
| rs4072917 | 0.00951 | 1.180826 | 0.941606 |
| rs2224272 | 0.201259 | 1.470884 | 0.942988 |
| rs429343 | 0.030519 | 0.938253 | 0.94436 |
| rs252749 | 0.010123 | 1.273975 | 0.946248 |
| rs6777784 | 0.007608 | 1.380991 | 0.948955 |
| rs2451746 | 0.166289 | 1.160326 | 0.951745 |
| rs9332817 | 0.009655 | 1.432968 | 0.951941 |
| rs1840969 | 0.175716 | 1.353552 | 0.953076 |
| rs13267015 | -0.00217 | 1.67118 | 0.953146 |
| rs823074 | 0.173021 | 1.327037 | 0.953757 |
| rs7102454 | 0.037976 | 1.002584 | 0.95384 |
| rs6870983 | 0.145089 | 0.875257 | 0.955345 |
| rs17551974 | 0.172815 | 1.373434 | 0.955436 |
| rs7006629 | 0.032458 | 1.148734 | 0.955877 |
| rs2283093 | 0.170843 | 1.376536 | 0.956678 |
| rs2425840 | 0.03192 | 1.18889 | 0.957004 |
| rs1454687 | 0.058022 | 0.708324 | 0.95724 |
| rs13380104 | 0.15085 | 1.025012 | 0.95738 |
| rs11115176 | 0.025457 | 1.321165 | 0.957406 |
| rs1394879 | 0.169414 | 1.402814 | 0.958299 |
| rs4963120 | 0.040748 | 1.078314 | 0.959123 |
| rs9615905 | 0.160591 | 1.260477 | 0.959173 |
| rs11753081 | 0.151707 | 1.11662 | 0.960259 |
| rs6419734 | 0.144795 | 1.029339 | 0.962244 |
| rs17069831 | 0.028886 | 1.421552 | 0.962331 |
| rs6138482 | 0.158937 | 1.33506 | 0.962438 |
| rs4757144 | 0.051233 | 0.973638 | 0.963313 |
| rs16966801 | 0.047528 | 1.083325 | 0.964297 |
| rs12364470 | 0.143214 | 1.086188 | 0.965376 |
| rs12675063 | 0.154519 | 1.426136 | 0.967302 |
| rs925421 | 0.035605 | 1.486161 | 0.967569 |
| rs1409818 | 0.047679 | 1.192252 | 0.967655 |
| rs10510419 | 0.053089 | 1.108561 | 0.969106 |
| rs506338 | 0.050101 | 1.194539 | 0.969333 |
| rs7083450 | 0.14576 | 1.34401 | 0.970501 |
| rs9547153 | 0.151738 | 1.509594 | 0.970577 |
| rs8097672 | 0.128038 | 0.888429 | 0.971292 |
| rs1956153 | 0.052166 | 1.239296 | 0.971767 |
| rs4985155 | 0.140173 | 1.311358 | 0.973163 |
| rs784944 | 0.049264 | 1.39444 | 0.973246 |
| rs1365466 | 0.132325 | 1.095021 | 0.97358 |
| **rsid** | **Beta** | **se** | **p (difference)^1^** |
| rs972283 | 0.048902 | 1.429951 | 0.973708 |
| rs1371108 | 0.135009 | 1.185553 | 0.97379 |
| rs2907948 | 0.139397 | 1.344457 | 0.974283 |
| rs9426003 | 0.138179 | 1.316321 | 0.974472 |
| rs17608150 | 0.135957 | 1.277648 | 0.975086 |
| rs13047416 | 0.120346 | 0.901395 | 0.978504 |
| rs762147 | 0.130815 | 1.398032 | 0.980163 |
| rs7801551 | 0.063632 | 1.415095 | 0.981732 |
| rs6804842 | 0.114185 | 0.797917 | 0.981875 |
| rs11629783 | 0.120105 | 1.06309 | 0.981951 |
| rs1689437 | 0.1202 | 1.103272 | 0.982539 |
| rs10460960 | 0.076535 | 1.018623 | 0.984729 |
| rs1430387 | 0.072198 | 1.247937 | 0.984761 |
| rs7600699 | 0.0699 | 1.391199 | 0.985011 |
| rs731834 | 0.07377 | 1.278019 | 0.9861 |
| rs1912631 | 0.076862 | 1.133991 | 0.986511 |
| rs905938 | 0.078474 | 1.060936 | 0.986795 |
| rs1634350 | 0.075546 | 1.267813 | 0.987106 |
| rs217433 | 0.121492 | 1.603391 | 0.987339 |
| rs9817583 | 0.080607 | 1.339332 | 0.990808 |
| rs13290794 | 0.085187 | 1.10744 | 0.992182 |
| rs1128249 | 0.106669 | 1.205002 | 0.992967 |
| rs2033529 | 0.091054 | 0.728972 | 0.994545 |
| rs10732321 | 0.0879 | 1.319202 | 0.995077 |
| rs2367112 | 0.090062 | 1.034043 | 0.995387 |
| rs7933205 | 0.102891 | 1.51787 | 0.996401 |
| rs2481665 | 0.098252 | 0.901571 | 0.998046 |
| ^1^p for the difference from the overall IVW estimate (beta: 0.10, s.e.0.03). To identify outliers, SNP-specific estimates were compared with the overall IVW estimate using a Bonferroni-corrected significance threshold of 0.05/965. Coefficients represent change in log-transformed percent absence per kg/m^2^ increase in BMI | | | |

| **Supplementary Table 13: Genetic models^1^: association of BMI with educational attainment at 16 and school absence at 14-16** | | | | |
| --- | --- | --- | --- | --- |
| **Direct associations with the BMI PGS** | **GCSE points** | **CI** | **Absence (% increase)** | **CI** |
| Standardized values of BMI PGS | -5.54 | -7.84,-3.26 | 2.72 | 0.57,4.91 |
|  |  |  |  |  |
| **Associations with genetically-instrumented BMI z-score** | **GCSE points** | **CI** | **Absence (% increase)** | **CI** |
| BMI at age 10 | 18.44 | -26.25, -10.64 | 10.12 | 2.68,18.10 |
| BMI at age 13 | -17.49 | -24.85,-10.15 | 9.58 | 2.58,17.07 |
| ^1^Excluding 24 SNPs identified as outliers in Two-Sample MR (Supplementary Table S12) | | | | |

| **Supplementary Table 14: Results of Two-Sample MR with 24 outlier SNPs excluded^1^** | | | | |
| --- | --- | --- | --- | --- |
| Method | N SNPs | b | se | p |
| Inverse variance weighted | 921 | -0.158576 | 0.0092251 | 3.17E-66 |
| Weighted median | 921 | -0.12675 | 0.0116438 | 1.35E-27 |
| Weighted mode | 921 | -0.062205 | 0.0323565 | 0.054852 |
| MR Egger | 921 | -0.084613 | 0.0281355 | 0.002707 |
| MR Egger - Intercept | 921 | -0.001144 | 0.0004114 | 0.005523 |
| ^1^24 SNPs were identified as significantly different from the overall IVW estimate for effect of BMI on years of schooling using a Bonferroni-corrected threshold of 5.29E-5. These were: rs10811901, rs10840606, rs11611246, rs11713193, rs11855853, rs12705977, rs12964689, rs1402025, rs1452075, rs1503526, rs16903285, rs2293605, rs2733287, rs329122, rs4916229, rs6449532, rs7334078, rs8047395, rs901630, rs903959, rs9267677, rs9816226, rs9817583, rs9965170 | | | | |

| **Supplementary Table 15: Associations of outlier BMI SNPs from TwoSample MR with other traits^1^** | | | | | | | | | |
| --- | --- | --- | --- | --- | --- | --- | --- | --- | --- |
| **Associations of outlying SNPs with BMI and adiposity traits** | | | | | | | | | |
| **SNP** | **Pos (hg19)** | **A1** | **A2** | **Trait** | **PMID** | **Beta** | **P** | **N** | **Unit** |
| rs11611246 | chr12:939480 | G | T | Arm fat mass left | UKBB | -0.02173 | 7.82E-14 | 331164 | IVNT |
| rs11713193 | chr3:49924424 | A | G | Arm fat mass left | UKBB | 0.02705 | 5.85E-30 | 331164 | IVNT |
| rs11855853 | chr15:78012618 | C | T | Arm fat mass left | UKBB | 0.01712 | 1.93E-10 | 331164 | IVNT |
| rs12964689 | chr18:21116998 | A | G | Arm fat mass left | UKBB | 0.01992 | 5.97E-17 | 331164 | IVNT |
| rs1503526 | chr5:63020706 | T | C | Arm fat mass left | UKBB | -0.01683 | 1.62E-12 | 331164 | IVNT |
| rs16903285 | chr5:87978252 | C | T | Arm fat mass left | UKBB | 0.03121 | 1.74E-18 | 331164 | IVNT |
| rs2733287 | chr12:41880909 | C | A | Arm fat mass left | UKBB | 0.01366 | 9.9E-09 | 331164 | IVNT |
| rs329122 | chr5:133864599 | A | G | Arm fat mass left | UKBB | -0.01543 | 1.67E-10 | 331164 | IVNT |
| rs8047395 | chr16:53798523 | A | G | Arm fat mass left | UKBB | 0.05295 | 3.9E-109 | 331164 | IVNT |
| rs901630 | chr6:98539519 | C | T | Arm fat mass left | UKBB | 0.01805 | 1.28E-13 | 331164 | IVNT |
| rs9816226 | chr3:185834499 | A | T | Arm fat mass left | UKBB | -0.0237 | 1.39E-14 | 331164 | IVNT |
| rs11611246 | chr12:939480 | G | T | Arm fat mass right | UKBB | -0.02216 | 2.49E-14 | 331226 | IVNT |
| rs11713193 | chr3:49924424 | A | G | Arm fat mass right | UKBB | 0.02733 | 1.42E-30 | 331226 | IVNT |
| rs11855853 | chr15:78012618 | C | T | Arm fat mass right | UKBB | 0.01776 | 3.94E-11 | 331226 | IVNT |
| rs12964689 | chr18:21116998 | A | G | Arm fat mass right | UKBB | 0.02072 | 3.17E-18 | 331226 | IVNT |
| rs1503526 | chr5:63020706 | T | C | Arm fat mass right | UKBB | -0.01646 | 4.76E-12 | 331226 | IVNT |
| rs16903285 | chr5:87978252 | C | T | Arm fat mass right | UKBB | 0.031 | 2.89E-18 | 331226 | IVNT |
| rs2733287 | chr12:41880909 | C | A | Arm fat mass right | UKBB | 0.01343 | 1.72E-08 | 331226 | IVNT |
| rs329122 | chr5:133864599 | A | G | Arm fat mass right | UKBB | -0.01542 | 1.7E-10 | 331226 | IVNT |
| rs8047395 | chr16:53798523 | A | G | Arm fat mass right | UKBB | 0.05302 | 1.7E-109 | 331226 | IVNT |
| rs901630 | chr6:98539519 | C | T | Arm fat mass right | UKBB | 0.01723 | 1.55E-12 | 331226 | IVNT |
| rs9267677 | chr6:31892641 | C | T | Arm fat mass right | UKBB | 0.02295 | 1.89E-08 | 331226 | IVNT |
| rs9816226 | chr3:185834499 | A | T | Arm fat mass right | UKBB | -0.02417 | 4.12E-15 | 331226 | IVNT |
| rs11611246 | chr12:939480 | G | T | Arm fat percentage left | UKBB | -0.01375 | 9.39E-10 | 331198 | IVNT |
| rs11713193 | chr3:49924424 | A | G | Arm fat percentage left | UKBB | 0.01798 | 1.29E-22 | 331198 | IVNT |
| rs11855853 | chr15:78012618 | C | T | Arm fat percentage left | UKBB | 0.01187 | 1.08E-08 | 331198 | IVNT |
| **SNP** | **Pos (hg19)** | **A1** | **A2** | **Trait** | **PMID** | **Beta** | **P** | **N** | **Unit** |
| rs12964689 | chr18:21116998 | A | G | Arm fat percentage left | UKBB | 0.0125 | 1.07E-11 | 331198 | IVNT |
| rs1503526 | chr5:63020706 | T | C | Arm fat percentage left | UKBB | -0.01315 | 8.85E-13 | 331198 | IVNT |
| rs16903285 | chr5:87978252 | C | T | Arm fat percentage left | UKBB | 0.02415 | 1.54E-18 | 331198 | IVNT |
| rs329122 | chr5:133864599 | A | G | Arm fat percentage left | UKBB | -0.0109 | 5.12E-09 | 331198 | IVNT |
| rs7334078 | chr13:99120484 | C | T | Arm fat percentage left | UKBB | -0.01315 | 1.17E-10 | 331198 | IVNT |
| rs8047395 | chr16:53798523 | A | G | Arm fat percentage left | UKBB | 0.03605 | 3.14E-85 | 331198 | IVNT |
| rs901630 | chr6:98539519 | C | T | Arm fat percentage left | UKBB | 0.01445 | 1.61E-14 | 331198 | IVNT |
| rs9816226 | chr3:185834499 | A | T | Arm fat percentage left | UKBB | -0.01507 | 2.31E-10 | 331198 | IVNT |
| rs11611246 | chr12:939480 | G | T | Arm fat percentage right | UKBB | -0.01365 | 1.45E-09 | 331249 | IVNT |
| rs11713193 | chr3:49924424 | A | G | Arm fat percentage right | UKBB | 0.0178 | 5.37E-22 | 331249 | IVNT |
| rs11855853 | chr15:78012618 | C | T | Arm fat percentage right | UKBB | 0.01281 | 8.16E-10 | 331249 | IVNT |
| rs12964689 | chr18:21116998 | A | G | Arm fat percentage right | UKBB | 0.01252 | 1.21E-11 | 331249 | IVNT |
| rs1503526 | chr5:63020706 | T | C | Arm fat percentage right | UKBB | -0.01304 | 1.75E-12 | 331249 | IVNT |
| rs16903285 | chr5:87978252 | C | T | Arm fat percentage right | UKBB | 0.02277 | 1.61E-16 | 331249 | IVNT |
| rs329122 | chr5:133864599 | A | G | Arm fat percentage right | UKBB | -0.01051 | 2.06E-08 | 331249 | IVNT |
| rs7334078 | chr13:99120484 | C | T | Arm fat percentage right | UKBB | -0.01319 | 1.24E-10 | 331249 | IVNT |
| rs8047395 | chr16:53798523 | A | G | Arm fat percentage right | UKBB | 0.0357 | 7.08E-83 | 331249 | IVNT |
| rs901630 | chr6:98539519 | C | T | Arm fat percentage right | UKBB | 0.01374 | 3.78E-13 | 331249 | IVNT |
| rs9267677 | chr6:31892641 | C | T | Arm fat percentage right | UKBB | 0.01783 | 1.85E-08 | 331249 | IVNT |
| rs9816226 | chr3:185834499 | A | T | Arm fat percentage right | UKBB | -0.01558 | 6.94E-11 | 331249 | IVNT |
| rs10811901 | chr9:23356935 | A | G | Arm fat-free mass left | UKBB | 0.008957 | 9.69E-09 | 331159 | IVNT |
| rs11611246 | chr12:939480 | G | T | Arm fat-free mass left | UKBB | -0.01403 | 1.01E-13 | 331159 | IVNT |
| rs11713193 | chr3:49924424 | A | G | Arm fat-free mass left | UKBB | 0.01578 | 1.54E-24 | 331159 | IVNT |
| rs12964689 | chr18:21116998 | A | G | Arm fat-free mass left | UKBB | 0.01422 | 3.3E-20 | 331159 | IVNT |
| rs2293605 | chr3:184044433 | T | C | Arm fat-free mass left | UKBB | -0.01937 | 3.39E-16 | 331159 | IVNT |
| rs2733287 | chr12:41880909 | C | A | Arm fat-free mass left | UKBB | 0.00978 | 2.52E-10 | 331159 | IVNT |
| rs329122 | chr5:133864599 | A | G | Arm fat-free mass left | UKBB | -0.008039 | 2.89E-07 | 331159 | IVNT |
| rs6449532 | chr5:60715446 | C | T | Arm fat-free mass left | UKBB | 0.008998 | 2.22E-08 | 331159 | IVNT |
| rs8047395 | chr16:53798523 | A | G | Arm fat-free mass left | UKBB | 0.03061 | 4.4E-87 | 331159 | IVNT |
| rs9816226 | chr3:185834499 | A | T | Arm fat-free mass left | UKBB | -0.01575 | 3.06E-15 | 331159 | IVNT |
| **SNP** | **Pos (hg19)** | **A1** | **A2** | **Trait** | **PMID** | **Beta** | **P** | **N** | **Unit** |
| rs10811901 | chr9:23356935 | A | G | Arm fat-free mass right | UKBB | 0.008686 | 1.38E-08 | 331221 | IVNT |
| rs11611246 | chr12:939480 | G | T | Arm fat-free mass right | UKBB | -0.01327 | 7.23E-13 | 331221 | IVNT |
| rs11713193 | chr3:49924424 | A | G | Arm fat-free mass right | UKBB | 0.01487 | 8.38E-23 | 331221 | IVNT |
| rs12964689 | chr18:21116998 | A | G | Arm fat-free mass right | UKBB | 0.01342 | 7.78E-19 | 331221 | IVNT |
| rs2293605 | chr3:184044433 | T | C | Arm fat-free mass right | UKBB | -0.01913 | 2.03E-16 | 331221 | IVNT |
| rs2733287 | chr12:41880909 | C | A | Arm fat-free mass right | UKBB | 0.008592 | 1.42E-08 | 331221 | IVNT |
| rs329122 | chr5:133864599 | A | G | Arm fat-free mass right | UKBB | -0.007173 | 0.000003 | 331221 | IVNT |
| rs6449532 | chr5:60715446 | C | T | Arm fat-free mass right | UKBB | 0.008761 | 2.73E-08 | 331221 | IVNT |
| rs8047395 | chr16:53798523 | A | G | Arm fat-free mass right | UKBB | 0.02896 | 2.82E-81 | 331221 | IVNT |
| rs9816226 | chr3:185834499 | A | T | Arm fat-free mass right | UKBB | -0.01399 | 8.97E-13 | 331221 | IVNT |
| rs10811901 | chr9:23356935 | A | G | Arm predicted mass left | UKBB | 0.008973 | 8.01E-09 | 331146 | IVNT |
| rs11611246 | chr12:939480 | G | T | Arm predicted mass left | UKBB | -0.0141 | 6.26E-14 | 331146 | IVNT |
| rs11713193 | chr3:49924424 | A | G | Arm predicted mass left | UKBB | 0.01588 | 5.06E-25 | 331146 | IVNT |
| rs12964689 | chr18:21116998 | A | G | Arm predicted mass left | UKBB | 0.01362 | 8.42E-19 | 331146 | IVNT |
| rs2293605 | chr3:184044433 | T | C | Arm predicted mass left | UKBB | -0.01852 | 4.87E-15 | 331146 | IVNT |
| rs2733287 | chr12:41880909 | C | A | Arm predicted mass left | UKBB | 0.009769 | 2.26E-10 | 331146 | IVNT |
| rs329122 | chr5:133864599 | A | G | Arm predicted mass left | UKBB | -0.007731 | 7.33E-07 | 331146 | IVNT |
| rs6449532 | chr5:60715446 | C | T | Arm predicted mass left | UKBB | 0.008903 | 2.76E-08 | 331146 | IVNT |
| rs8047395 | chr16:53798523 | A | G | Arm predicted mass left | UKBB | 0.03007 | 1.09E-84 | 331146 | IVNT |
| rs9816226 | chr3:185834499 | A | T | Arm predicted mass left | UKBB | -0.01528 | 1.58E-14 | 331146 | IVNT |
| rs10811901 | chr9:23356935 | A | G | Arm predicted mass right | UKBB | 0.008899 | 5.36E-09 | 331216 | IVNT |
| rs11611246 | chr12:939480 | G | T | Arm predicted mass right | UKBB | -0.01296 | 1.99E-12 | 331216 | IVNT |
| rs11713193 | chr3:49924424 | A | G | Arm predicted mass right | UKBB | 0.01508 | 1.47E-23 | 331216 | IVNT |
| rs12964689 | chr18:21116998 | A | G | Arm predicted mass right | UKBB | 0.01334 | 9.12E-19 | 331216 | IVNT |
| rs2293605 | chr3:184044433 | T | C | Arm predicted mass right | UKBB | -0.01893 | 3.26E-16 | 331216 | IVNT |
| rs2733287 | chr12:41880909 | C | A | Arm predicted mass right | UKBB | 0.008367 | 3E-08 | 331216 | IVNT |
| rs329122 | chr5:133864599 | A | G | Arm predicted mass right | UKBB | -0.00758 | 7.3E-07 | 331216 | IVNT |
| rs8047395 | chr16:53798523 | A | G | Arm predicted mass right | UKBB | 0.02913 | 9.7E-83 | 331216 | IVNT |
| rs9816226 | chr3:185834499 | A | T | Arm predicted mass right | UKBB | -0.01407 | 5.52E-13 | 331216 | IVNT |
| rs9816226 | chr3:185834499 | A | T | BMI tails upper & lower 5th percentiles | 23563607 | NA | 1.25E-08 | 16068 | - |
| **SNP** | **Pos (hg19)** | **A1** | **A2** | **Trait** | **PMID** | **Beta** | **P** | **N** | **Unit** |
| rs11611246 | chr12:939480 | G | T | Body fat percentage | UKBB | -0.01259 | 3.16E-08 | 331117 | IVNT |
| rs11713193 | chr3:49924424 | A | G | Body fat percentage | UKBB | 0.01773 | 1.66E-21 | 331117 | IVNT |
| rs11855853 | chr15:78012618 | C | T | Body fat percentage | UKBB | 0.01255 | 2.39E-09 | 331117 | IVNT |
| rs12964689 | chr18:21116998 | A | G | Body fat percentage | UKBB | 0.01465 | 3.7E-15 | 331117 | IVNT |
| rs1503526 | chr5:63020706 | T | C | Body fat percentage | UKBB | -0.01219 | 6.2E-11 | 331117 | IVNT |
| rs16903285 | chr5:87978252 | C | T | Body fat percentage | UKBB | 0.02263 | 4.31E-16 | 331117 | IVNT |
| rs329122 | chr5:133864599 | A | G | Body fat percentage | UKBB | -0.009204 | 1.11E-06 | 331117 | IVNT |
| rs7334078 | chr13:99120484 | C | T | Body fat percentage | UKBB | -0.01449 | 2.34E-12 | 331117 | IVNT |
| rs8047395 | chr16:53798523 | A | G | Body fat percentage | UKBB | 0.0321 | 2.7E-66 | 331117 | IVNT |
| rs8047395 | chr16:53798523 | A | G | Body fat percentage | 26833246 | 0.0416 | 1.87E-18 | 98145 | IVNT |
| rs901630 | chr6:98539519 | C | T | Body fat percentage | UKBB | 0.01661 | 3.05E-18 | 331117 | IVNT |
| rs10811901 | chr9:23356935 | A | G | Body mass index | 28892062 | 0.015 | 1E-08 | - | kg/m |
| rs11611246 | chr12:939480 | G | T | Body mass index | UKBB | -0.02291 | 5.96E-15 | 336107 | IVNT |
| rs11713193 | chr3:49924424 | A | G | Body mass index | UKBB | 0.03142 | 4.09E-39 | 336107 | IVNT |
| rs11855853 | chr15:78012618 | C | T | Body mass index | UKBB | 0.0198 | 2.98E-13 | 336107 | IVNT |
| rs12964689 | chr18:21116998 | A | G | Body mass index | UKBB | 0.01792 | 8.59E-14 | 336107 | IVNT |
| rs12964689 | chr18:21116998 | A | G | Body mass index | 25673413 | 0.0202 | 2.01E-08 | 235808 | IVNT |
| rs1503526 | chr5:63020706 | T | C | Body mass index | UKBB | -0.01688 | 2.19E-12 | 336107 | IVNT |
| rs1503526 | chr5:63020706 | T | C | Body mass index | 23754948 | -0.02228 | 2.93E-07 | 126574 | IVNT |
| rs1503526 | chr5:63020706 | T | C | Body mass index | 20935630 | NA | 9.33E-07 | 123864 | IVNT |
| rs1503526 | chr5:63020706 | T | C | Body mass index | 20935630 | NA | 9.33E-07 | 123865 | - |
| rs16903285 | chr5:87978252 | C | T | Body mass index | UKBB | 0.03299 | 3.94E-20 | 336107 | IVNT |
| rs16903285 | chr5:87978252 | C | T | Body mass index | 28892062 | 0.025 | 6E-16 | - | kg/m |
| rs16903285 | chr5:87978252 | C | T | Body mass index | 28892062 | 0.02723 | 4.45E-13 | 173430 | IVNT |
| rs2733287 | chr12:41880909 | C | A | Body mass index | UKBB | 0.01371 | 1.21E-08 | 336107 | IVNT |
| rs329122 | chr5:133864599 | A | G | Body mass index | UKBB | -0.01759 | 5.32E-13 | 336107 | IVNT |
| rs329122 | chr5:133864599 | A | G | Body mass index | 28892062 | 0.013 | 6E-08 | - | kg/m |
| rs329122 | chr5:133864599 | A | G | Body mass index | 28892062 | -0.01901 | 2.73E-07 | 173430 | IVNT |
| rs4916229 | chr1:171443368 | C | G | Body mass index | UKBB | -0.02044 | 5.59E-07 | 336107 | IVNT |
| rs8047395 | chr16:53798523 | A | G | Body mass index | UKBB | 0.05881 | 9E-132 | 336107 | IVNT |
| **SNP** | **Pos (hg19)** | **A1** | **A2** | **Trait** | **PMID** | **Beta** | **P** | **N** | **Unit** |
| rs8047395 | chr16:53798523 | A | G | Body mass index | 25673413 | 0.0656 | 2.1E-102 | 337327 | IVNT |
| rs8047395 | chr16:53798523 | A | G | Body mass index | 25673413 | 0.0677 | 9.5E-100 | 320263 | IVNT |
| rs8047395 | chr16:53798523 | A | G | Body mass index | 23754948 | 0.06527 | 1.77E-44 | 126541 | IVNT |
| rs8047395 | chr16:53798523 | A | G | Body mass index | 20935630 | NA | 2.5E-37 | 123830 | IVNT |
| rs8047395 | chr16:53798523 | A | G | Body mass index | 20935630 | NA | 2.5E-37 | 123865 | - |
| rs8047395 | chr16:53798523 | A | G | Body mass index | 19557197 | NA | 3.5E-13 | 31373 | - |
| rs8047395 | chr16:53798523 | A | G | Body mass index | 28892062 | 0.02566 | 4.4E-11 | 173430 | IVNT |
| rs8047395 | chr16:53798523 | A | G | Body mass index | 21935397 | NA | 5.26E-10 | 5373 | - |
| rs901630 | chr6:98539519 | C | T | Body mass index | UKBB | 0.01822 | 1.3E-13 | 336107 | IVNT |
| rs9267677 | chr6:31892641 | C | T | Body mass index | UKBB | 0.0309 | 6.5E-14 | 336107 | IVNT |
| rs9816226 | chr3:185834499 | A | T | Body mass index | 29273807 | -0.034 | 2.5E-30 | 526508 | IVNT |
| rs9816226 | chr3:185834499 | A | T | Body mass index | 29273807 | -0.035 | 2.7E-25 | 449889 | IVNT |
| rs9816226 | chr3:185834499 | A | T | Body mass index | 25673413 | -0.0397 | 6.03E-24 | 326506 | IVNT |
| rs9816226 | chr3:185834499 | A | T | Body mass index | UKBB | -0.0301 | 3.46E-22 | 336107 | IVNT |
| rs9816226 | chr3:185834499 | A | T | Body mass index | 25673413 | -0.0393 | 1.47E-21 | 309442 | IVNT |
| rs9816226 | chr3:185834499 | A | T | Body mass index | 20935630 | NA | 1.69E-18 | 123865 | - |
| rs9816226 | chr3:185834499 | A | T | Body mass index | 23001569 | NA | 1.69E-18 | 78436 | - |
| rs9816226 | chr3:185834499 | A | T | Body mass index | 20935630 | -0.14 | 2E-18 | - | kg/m2 increase |
| rs9816226 | chr3:185834499 | A | T | Body mass index | 20935630 | NA | 2E-18 | - | - |
| rs9816226 | chr3:185834499 | A | T | Body mass index | 23754948 | -0.04644 | 7.42E-16 | 126570 | IVNT |
| rs9816226 | chr3:185834499 | A | T | Body mass index | 20935630 | NA | 7.61E-14 | 123859 | IVNT |
| rs9816226 | chr3:185834499 | A | T | Body mass index 25 kgm2 | 23563607 | NA | 1.81E-14 | 16068 | - |
| rs9816226 | chr3:185834499 | A | T | Body mass index 30 kgm2 | 23563607 | NA | 1.57E-13 | 16068 | - |
| rs9816226 | chr3:185834499 | A | T | Body mass index 35 kgm2 | 23563607 | NA | 9.45E-09 | 16068 | - |
| rs8047395 | chr16:53798523 | A | G | Body mass index adjsuted for physical activity in males | 28448500 | 0.0611 | 3.06E-30 | 83695 | IVNT |
| rs329122 | chr5:133864599 | A | G | Body mass index adjusted for physical activity | 28448500 | -0.016 | 0.000009 | 195699 | IVNT |
| rs8047395 | chr16:53798523 | A | G | Body mass index adjusted for physical activity | 28448500 | 0.0627 | 0 | 178579 | IVNT |
| **SNP** | **Pos (hg19)** | **A1** | **A2** | **Trait** | **PMID** | **Beta** | **P** | **N** | **Unit** |
| rs8047395 | chr16:53798523 | A | G | Body mass index adjusted for physical activity | 28448500 | 0.0589 | 0 | 198607 | IVNT |
| rs9816226 | chr3:185834499 | A | T | Body mass index adjusted for physical activity | 28448500 | -0.0381 | 9.45E-15 | 179193 | IVNT |
| rs9816226 | chr3:185834499 | A | T | Body mass index adjusted for physical activity | 28448500 | -0.034 | 2.4E-13 | 199222 | IVNT |
| rs8047395 | chr16:53798523 | A | G | Body mass index adjusted for smoking | 28443625 | 0.0637 | 1.08E-70 | 205013 | IVNT |
| rs8047395 | chr16:53798523 | A | G | Body mass index adjusted for smoking | 28443625 | 0.0588 | 1.51E-68 | 235383 | IVNT |
| rs9816226 | chr3:185834499 | A | T | Body mass index adjusted for smoking | 28443625 | -0.0364 | 3.02E-15 | 195615 | IVNT |
| rs9816226 | chr3:185834499 | A | T | Body mass index adjusted for smoking | 28443625 | -0.0315 | 1.42E-13 | 225988 | IVNT |
| rs8047395 | chr16:53798523 | A | G | Body mass index adjusted for smoking in females | 28443625 | 0.057 | 8.41E-39 | 134560 | IVNT |
| rs9816226 | chr3:185834499 | A | T | Body mass index adjusted for smoking in females | 28443625 | -0.0337 | 2.34E-09 | 129150 | IVNT |
| rs8047395 | chr16:53798523 | A | G | Body mass index adjusted for smoking in males | 28443625 | 0.0608 | 5.71E-36 | 100824 | IVNT |
| rs8047395 | chr16:53798523 | A | G | Body mass index ajusted for physical activity in females | 28448500 | 0.0573 | 2.71E-34 | 114912 | IVNT |
| rs9816226 | chr3:185834499 | A | T | Body mass index ajusted for physical activity in females | 28448500 | -0.0382 | 1.12E-10 | 115226 | IVNT |
| rs8047395 | chr16:53798523 | A | G | Body mass index in female non-smokers | 28443625 | 0.0533 | 1.68E-28 | 111366 | IVNT |
| rs8047395 | chr16:53798523 | A | G | Body mass index in female smokers | 28443625 | 0.0744 | 9.84E-15 | 25971 | IVNT |
| rs12964689 | chr18:21116998 | A | G | Body mass index in females | 25673413 | 0.0264 | 1.94E-08 | 131933 | IVNT |
| rs8047395 | chr16:53798523 | A | G | Body mass index in females | 25673413 | 0.0663 | 1.27E-58 | 171007 | IVNT |
| rs8047395 | chr16:53798523 | A | G | Body mass index in females | 23754948 | 0.058 | 3.28E-19 | 67941 | IVNT |
| rs9816226 | chr3:185834499 | A | T | Body mass index in females | 25673413 | -0.0406 | 6.73E-15 | 165187 | IVNT |
| rs9816226 | chr3:185834499 | A | T | Body mass index in females | 23754948 | -0.045 | 2.53E-08 | 67955 | IVNT |
| rs8047395 | chr16:53798523 | A | G | Body mass index in females greater than 50 years of age | 26426971 | 0.069 | 1.1E-44 | 104323 | IVNT |
| rs9816226 | chr3:185834499 | A | T | Body mass index in females greater than 50 years of age | 26426971 | -0.04 | 1.2E-10 | 102876 | IVNT |
| rs8047395 | chr16:53798523 | A | G | Body mass index in females less than or equal to 50 years of age | 26426971 | 0.072 | 3.1E-32 | 65130 | IVNT |
|  |  |  |  |  |  |  |  |  |  |
| **SNP** | **Pos (hg19)** | **A1** | **A2** | **Trait** | **PMID** | **Beta** | **P** | **N** | **Unit** |
| rs9816226 | chr3:185834499 | A | T | Body mass index in females less than or equal to 50 years of age | 26426971 | -0.051 | 3E-10 | 60805 | IVNT |
| rs8047395 | chr16:53798523 | A | G | Body mass index in male non-smokers | 28443625 | 0.0579 | 5.79E-26 | 77019 | IVNT |
| rs8047395 | chr16:53798523 | A | G | Body mass index in male smokers | 28443625 | 0.0731 | 7.09E-13 | 24451 | IVNT |
| rs8047395 | chr16:53798523 | A | G | Body mass index in males | 25673413 | 0.0693 | 2.04E-62 | 151967 | IVNT |
| rs8047395 | chr16:53798523 | A | G | Body mass index in males | 23754948 | 0.073 | 1.36E-27 | 58601 | IVNT |
| rs9816226 | chr3:185834499 | A | T | Body mass index in males | 25673413 | -0.0394 | 3.94E-13 | 146966 | IVNT |
| rs9816226 | chr3:185834499 | A | T | Body mass index in males | 23754948 | -0.048 | 5.92E-09 | 58615 | IVNT |
| rs1503526 | chr5:63020706 | T | C | Body mass index in males greater than 50 years of age | 26426971 | -0.023 | 5.4E-06 | 87077 | IVNT |
| rs8047395 | chr16:53798523 | A | G | Body mass index in males greater than 50 years of age | 26426971 | 0.061 | 9.7E-34 | 90688 | IVNT |
| rs8047395 | chr16:53798523 | A | G | Body mass index in males less than or equal to 50 years of age | 26426971 | 0.08 | 2E-31 | 49698 | IVNT |
| rs8047395 | chr16:53798523 | A | G | Body mass index in non-smokers | 28443625 | 0.0596 | 2.16E-48 | 162548 | IVNT |
| rs8047395 | chr16:53798523 | A | G | Body mass index in non-smokers | 28443625 | 0.0548 | 3.06E-47 | 188385 | IVNT |
| rs9816226 | chr3:185834499 | A | T | Body mass index in non-smokers | 28443625 | -0.0326 | 5.79E-10 | 155250 | IVNT |
| rs9816226 | chr3:185834499 | A | T | Body mass index in non-smokers | 28443625 | -0.0279 | 8.36E-09 | 181091 | IVNT |
| rs8047395 | chr16:53798523 | A | G | Body mass index in physically active females | 28448500 | 0.052 | 1.38E-23 | 88369 | IVNT |
| rs9816226 | chr3:185834499 | A | T | Body mass index in physically active females | 28448500 | -0.0381 | 4.02E-09 | 88580 | IVNT |
| rs8047395 | chr16:53798523 | A | G | Body mass index in physically active indivdiuals | 28448500 | 0.0588 | 0 | 136822 | IVNT |
| rs8047395 | chr16:53798523 | A | G | Body mass index in physically active indivdiuals | 28448500 | 0.0552 | 0 | 148808 | IVNT |
| rs9816226 | chr3:185834499 | A | T | Body mass index in physically active indivdiuals | 28448500 | -0.0375 | 8.11E-12 | 137251 | IVNT |
| rs9816226 | chr3:185834499 | A | T | Body mass index in physically active indivdiuals | 28448500 | -0.0335 | 1.55E-10 | 149238 | IVNT |
| rs8047395 | chr16:53798523 | A | G | Body mass index in physically active males | 28448500 | 0.0593 | 1.16E-21 | 60958 | IVNT |
| rs8047395 | chr16:53798523 | A | G | Body mass index in physically inactive females | 28448500 | 0.07 | 8.62E-13 | 26568 | IVNT |
|  |  |  |  |  |  |  |  |  |  |
| **SNP** | **Pos (hg19)** | **A1** | **A2** | **Trait** | **PMID** | **Beta** | **P** | **N** | **Unit** |
| rs8047395 | chr16:53798523 | A | G | Body mass index in physically inactive indivdiuals | 28448500 | 0.072 | 6.28E-20 | 41610 | IVNT |
| rs8047395 | chr16:53798523 | A | G | Body mass index in physically inactive indivdiuals | 28448500 | 0.0682 | 9.35E-20 | 46194 | IVNT |
| rs8047395 | chr16:53798523 | A | G | Body mass index in physically inactive males | 28448500 | 0.0613 | 4.07E-09 | 21869 | IVNT |
| rs8047395 | chr16:53798523 | A | G | Body mass index in smokers | 28443625 | 0.0779 | 1.38E-25 | 45320 | IVNT |
| rs8047395 | chr16:53798523 | A | G | Body mass index in smokers | 28443625 | 0.0729 | 1.24E-24 | 50422 | IVNT |
| rs9816226 | chr3:185834499 | A | T | Body mass index in smokers | 28443625 | -0.0538 | 3.31E-08 | 42078 | IVNT |
| rs8047395 | chr16:53798523 | A | G | Body mass index males | 28892062 | 0.02773 | 1.57E-07 | 90992 | IVNT |
| rs8047395 | chr16:53798523 | A | G | Body mass index tails | 23563607 | 0.26 | 2.8E-22 | 15961 | log OR |
| rs8047395 | chr16:53798523 | A | G | Childhood BMI | 26604143 | 0.0479 | 2.46E-08 | 31087 | SDS |
| rs8047395 | chr16:53798523 | A | G | Childhood obesity | 22484627 | 0.1634 | 4.08E-09 | 13848 | log OR |
| rs9816226 | chr3:185834499 | A | T | Extreme obesity with early age of onset | 23563609 | NA | 1.7E-218 | 6889 | - |
| rs11611246 | chr12:939480 | G | T | Hip circumference | UKBB | -0.02008 | 8.39E-12 | 336601 | IVNT |
| rs11713193 | chr3:49924424 | A | G | Hip circumference | UKBB | 0.01971 | 2.47E-16 | 336601 | IVNT |
| rs12964689 | chr18:21116998 | A | G | Hip circumference | UKBB | 0.02054 | 1.34E-17 | 336601 | IVNT |
| rs12964689 | chr18:21116998 | A | G | Hip circumference | 25673412 | 0.027 | 1.5E-09 | 145368 | IVNT |
| rs12964689 | chr18:21116998 | A | G | Hip circumference | 25673412 | 0.027 | 1.5E-09 | 145368 | IVNT |
| rs1503526 | chr5:63020706 | T | C | Hip circumference | UKBB | -0.0132 | 4.12E-08 | 336601 | IVNT |
| rs16903285 | chr5:87978252 | C | T | Hip circumference | UKBB | 0.02747 | 2.12E-14 | 336601 | IVNT |
| rs2733287 | chr12:41880909 | C | A | Hip circumference | UKBB | 0.01321 | 4.06E-08 | 336601 | IVNT |
| rs329122 | chr5:133864599 | A | G | Hip circumference | UKBB | -0.01652 | 1.29E-11 | 336601 | IVNT |
| rs8047395 | chr16:53798523 | A | G | Hip circumference | UKBB | 0.04992 | 2.93E-95 | 336601 | IVNT |
| rs8047395 | chr16:53798523 | A | G | Hip circumference | 25673412 | 0.06 | 1.1E-61 | 212403 | IVNT |
| rs8047395 | chr16:53798523 | A | G | Hip circumference | 25673412 | 0.057 | 1.7E-57 | 226787 | IVNT |
| rs9816226 | chr3:185834499 | A | T | Hip circumference | UKBB | -0.01879 | 1.54E-09 | 336601 | IVNT |
| rs9816226 | chr3:185834499 | A | T | Hip circumference | 25673412 | -0.027 | 6.1E-09 | 214703 | IVNT |
| rs9816226 | chr3:185834499 | A | T | Hip circumference | 25673412 | -0.027 | 1.9E-08 | 200319 | IVNT |
| rs12964689 | chr18:21116998 | A | G | Hip circumference in females | 25673412 | 0.033 | 3.1E-09 | 88066 | IVNT |
| **SNP** | **Pos (hg19)** | **A1** | **A2** | **Trait** | **PMID** | **Beta** | **P** | **N** | **Unit** |
| rs8047395 | chr16:53798523 | A | G | Hip circumference in females | 25673412 | 0.059 | 5.3E-37 | 124760 | IVNT |
| rs8047395 | chr16:53798523 | A | G | Hip circumference in males | 25673412 | 0.056 | 1E-30 | 100094 | IVNT |
| rs11611246 | chr12:939480 | G | T | Impedance of arm left | UKBB | 0.01365 | 7.31E-11 | 331292 | IVNT |
| rs11713193 | chr3:49924424 | A | G | Impedance of arm left | UKBB | -0.01807 | 5.47E-26 | 331292 | IVNT |
| rs2293605 | chr3:184044433 | T | C | Impedance of arm left | UKBB | 0.01852 | 2.19E-12 | 331292 | IVNT |
| rs329122 | chr5:133864599 | A | G | Impedance of arm left | UKBB | 0.009552 | 4.07E-08 | 331292 | IVNT |
| rs4916229 | chr1:171443368 | C | G | Impedance of arm left | UKBB | 0.01305 | 7.63E-06 | 331292 | IVNT |
| rs8047395 | chr16:53798523 | A | G | Impedance of arm left | UKBB | -0.03048 | 2.54E-70 | 331292 | IVNT |
| rs9267677 | chr6:31892641 | C | T | Impedance of arm left | UKBB | -0.01978 | 1.82E-11 | 331292 | IVNT |
| rs9816226 | chr3:185834499 | A | T | Impedance of arm left | UKBB | 0.02108 | 2.1E-21 | 331292 | IVNT |
| rs10840606 | chr11:2234690 | A | G | Impedance of arm right | UKBB | 0.01234 | 4.51E-08 | 331279 | IVN |
| rs11611246 | chr12:939480 | G | T | Impedance of arm right | UKBB | 0.01255 | 1.83E-09 | 331279 | IVNT |
| rs11713193 | chr3:49924424 | A | G | Impedance of arm right | UKBB | -0.01827 | 9.96E-27 | 331279 | IVNT |
| rs2293605 | chr3:184044433 | T | C | Impedance of arm right | UKBB | 0.01883 | 7.36E-13 | 331279 | IVNT |
| rs329122 | chr5:133864599 | A | G | Impedance of arm right | UKBB | 0.009891 | 1.15E-08 | 331279 | IVNT |
| rs8047395 | chr16:53798523 | A | G | Impedance of arm right | UKBB | -0.03007 | 4.58E-69 | 331279 | IVNT |
| rs9267677 | chr6:31892641 | C | T | Impedance of arm right | UKBB | -0.01784 | 1.14E-09 | 331279 | IVNT |
| rs9816226 | chr3:185834499 | A | T | Impedance of arm right | UKBB | 0.01955 | 8.68E-19 | 331279 | IVNT |
| rs11611246 | chr12:939480 | G | T | Impedance of leg left | UKBB | 0.016 | 3.38E-09 | 331296 | IVNT |
| rs11713193 | chr3:49924424 | A | G | Impedance of leg left | UKBB | -0.01651 | 8.69E-14 | 331296 | IVNT |
| rs16903285 | chr5:87978252 | C | T | Impedance of leg left | UKBB | -0.02094 | 2.51E-10 | 331296 | IVNT |
| rs2293605 | chr3:184044433 | T | C | Impedance of leg left | UKBB | 0.03029 | 5.76E-19 | 331296 | IVNT |
| rs329122 | chr5:133864599 | A | G | Impedance of leg left | UKBB | 0.01261 | 2.01E-08 | 331296 | IVNT |
| rs6449532 | chr5:60715446 | C | T | Impedance of leg left | UKBB | -0.01556 | 1.51E-11 | 331296 | IVNT |
| rs8047395 | chr16:53798523 | A | G | Impedance of leg left | UKBB | -0.04957 | 1.6E-110 | 331296 | IVNT |
| rs9816226 | chr3:185834499 | A | T | Impedance of leg left | UKBB | 0.03198 | 6.09E-29 | 331296 | IVNT |
| rs10811901 | chr9:23356935 | A | G | Impedance of leg right | UKBB | -0.01254 | 1.65E-08 | 331301 | IVNT |
| rs11611246 | chr12:939480 | G | T | Impedance of leg right | UKBB | 0.01698 | 2.56E-10 | 331301 | IVNT |
| rs11713193 | chr3:49924424 | A | G | Impedance of leg right | UKBB | -0.01592 | 4.24E-13 | 331301 | IVNT |
| rs16903285 | chr5:87978252 | C | T | Impedance of leg right | UKBB | -0.01811 | 3.53E-08 | 331301 | IVNT |
| **SNP** | **Pos (hg19)** | **A1** | **A2** | **Trait** | **PMID** | **Beta** | **P** | **N** | **Unit** |
| rs2293605 | chr3:184044433 | T | C | Impedance of leg right | UKBB | 0.03034 | 2.71E-19 | 331301 | IVNT |
| rs329122 | chr5:133864599 | A | G | Impedance of leg right | UKBB | 0.01365 | 9.24E-10 | 331301 | IVNT |
| rs6449532 | chr5:60715446 | C | T | Impedance of leg right | UKBB | -0.01621 | 1.41E-12 | 331301 | IVNT |
| rs8047395 | chr16:53798523 | A | G | Impedance of leg right | UKBB | -0.04767 | 7E-104 | 331301 | IVNT |
| rs9816226 | chr3:185834499 | A | T | Impedance of leg right | UKBB | 0.03405 | 4.54E-33 | 331301 | IVNT |
| rs10840606 | chr11:2234690 | A | G | Impedance of whole body | UKBB | 0.01392 | 1.25E-08 | 331284 | IVNT |
| rs11611246 | chr12:939480 | G | T | Impedance of whole body | UKBB | 0.01629 | 6.05E-13 | 331284 | IVNT |
| rs11713193 | chr3:49924424 | A | G | Impedance of whole body | UKBB | -0.01847 | 1.87E-23 | 331284 | IVNT |
| rs2293605 | chr3:184044433 | T | C | Impedance of whole body | UKBB | 0.02358 | 1.22E-16 | 331284 | IVNT |
| rs329122 | chr5:133864599 | A | G | Impedance of whole body | UKBB | 0.0115 | 9.29E-10 | 331284 | IVNT |
| rs6449532 | chr5:60715446 | C | T | Impedance of whole body | UKBB | -0.01342 | 3.46E-12 | 331284 | IVNT |
| rs8047395 | chr16:53798523 | A | G | Impedance of whole body | UKBB | -0.04133 | 7.6E-110 | 331284 | IVNT |
| rs9267677 | chr6:31892641 | C | T | Impedance of whole body | UKBB | -0.01885 | 3E-09 | 331284 | IVNT |
| rs9816226 | chr3:185834499 | A | T | Impedance of whole body | UKBB | 0.02733 | 3.77E-30 | 331284 | IVNT |
| rs11611246 | chr12:939480 | G | T | Leg fat mass left | UKBB | -0.01727 | 1.78E-13 | 331275 | IVNT |
| rs11713193 | chr3:49924424 | A | G | Leg fat mass left | UKBB | 0.02324 | 8.82E-34 | 331275 | IVNT |
| rs11855853 | chr15:78012618 | C | T | Leg fat mass left | UKBB | 0.01426 | 4.88E-11 | 331275 | IVNT |
| rs12964689 | chr18:21116998 | A | G | Leg fat mass left | UKBB | 0.01717 | 3.75E-19 | 331275 | IVNT |
| rs1503526 | chr5:63020706 | T | C | Leg fat mass left | UKBB | -0.01324 | 5.45E-12 | 331275 | IVNT |
| rs16903285 | chr5:87978252 | C | T | Leg fat mass left | UKBB | 0.02419 | 3.41E-17 | 331275 | IVNT |
| rs2733287 | chr12:41880909 | C | A | Leg fat mass left | UKBB | 0.0125 | 7.74E-11 | 331275 | IVNT |
| rs329122 | chr5:133864599 | A | G | Leg fat mass left | UKBB | -0.01152 | 3.35E-09 | 331275 | IVNT |
| rs8047395 | chr16:53798523 | A | G | Leg fat mass left | UKBB | 0.04235 | 2.2E-107 | 331275 | IVNT |
| rs901630 | chr6:98539519 | C | T | Leg fat mass left | UKBB | 0.01647 | 5.35E-17 | 331275 | IVNT |
| rs903959 | chr8:142630782 | A | T | Leg fat mass left | UKBB | 0.01113 | 1.41E-08 | 331275 | IVNT |
| rs9267677 | chr6:31892641 | C | T | Leg fat mass left | UKBB | 0.02282 | 4.24E-12 | 331275 | IVNT |
| rs9816226 | chr3:185834499 | A | T | Leg fat mass left | UKBB | -0.01798 | 4.47E-13 | 331275 | IVNT |
| rs11611246 | chr12:939480 | G | T | Leg fat mass right | UKBB | -0.01701 | 7.43E-13 | 331293 | IVNT |
| rs11713193 | chr3:49924424 | A | G | Leg fat mass right | UKBB | 0.02318 | 6.86E-33 | 331293 | IVNT |
| rs11855853 | chr15:78012618 | C | T | Leg fat mass right | UKBB | 0.01385 | 2.74E-10 | 331293 | IVNT |
| **SNP** | **Pos (hg19)** | **A1** | **A2** | **Trait** | **PMID** | **Beta** | **P** | **N** | **Unit** |
| rs12964689 | chr18:21116998 | A | G | Leg fat mass right | UKBB | 0.01752 | 1.87E-19 | 331293 | IVNT |
| rs1503526 | chr5:63020706 | T | C | Leg fat mass right | UKBB | -0.01342 | 4.99E-12 | 331293 | IVNT |
| rs16903285 | chr5:87978252 | C | T | Leg fat mass right | UKBB | 0.02499 | 7.38E-18 | 331293 | IVNT |
| rs2733287 | chr12:41880909 | C | A | Leg fat mass right | UKBB | 0.0124 | 1.8E-10 | 331293 | IVNT |
| rs329122 | chr5:133864599 | A | G | Leg fat mass right | UKBB | -0.01098 | 2.49E-08 | 331293 | IVNT |
| rs7334078 | chr13:99120484 | C | T | Leg fat mass right | UKBB | -0.01221 | 1.46E-08 | 331293 | IVNT |
| rs8047395 | chr16:53798523 | A | G | Leg fat mass right | UKBB | 0.0428 | 3.4E-107 | 331293 | IVNT |
| rs901630 | chr6:98539519 | C | T | Leg fat mass right | UKBB | 0.01683 | 2.59E-17 | 331293 | IVNT |
| rs9267677 | chr6:31892641 | C | T | Leg fat mass right | UKBB | 0.02231 | 2.13E-11 | 331293 | IVNT |
| rs9816226 | chr3:185834499 | A | T | Leg fat mass right | UKBB | -0.01712 | 9.25E-12 | 331293 | IVNT |
| rs11611246 | chr12:939480 | G | T | Leg fat percentage left | UKBB | -0.01039 | 2.19E-08 | 331278 | IVNT |
| rs11713193 | chr3:49924424 | A | G | Leg fat percentage left | UKBB | 0.01632 | 5.65E-27 | 331278 | IVNT |
| rs11855853 | chr15:78012618 | C | T | Leg fat percentage left | UKBB | 0.01157 | 1.51E-11 | 331278 | IVNT |
| rs12964689 | chr18:21116998 | A | G | Leg fat percentage left | UKBB | 0.01146 | 4.47E-14 | 331278 | IVNT |
| rs1503526 | chr5:63020706 | T | C | Leg fat percentage left | UKBB | -0.01042 | 7.18E-12 | 331278 | IVNT |
| rs16903285 | chr5:87978252 | C | T | Leg fat percentage left | UKBB | 0.01657 | 2.85E-13 | 331278 | IVNT |
| rs2733287 | chr12:41880909 | C | A | Leg fat percentage left | UKBB | 0.00844 | 2.84E-08 | 331278 | IVNT |
| rs329122 | chr5:133864599 | A | G | Leg fat percentage left | UKBB | -0.00727 | 2.39E-06 | 331278 | IVNT |
| rs7334078 | chr13:99120484 | C | T | Leg fat percentage left | UKBB | -0.0106 | 3.18E-10 | 331278 | IVNT |
| rs8047395 | chr16:53798523 | A | G | Leg fat percentage left | UKBB | 0.02573 | 4.53E-64 | 331278 | IVNT |
| rs901630 | chr6:98539519 | C | T | Leg fat percentage left | UKBB | 0.01371 | 1.15E-18 | 331278 | IVNT |
| rs9267677 | chr6:31892641 | C | T | Leg fat percentage left | UKBB | 0.01804 | 4.47E-12 | 331278 | IVNT |
| rs11713193 | chr3:49924424 | A | G | Leg fat percentage right | UKBB | 0.01636 | 1.9E-26 | 331296 | IVNT |
| rs11855853 | chr15:78012618 | C | T | Leg fat percentage right | UKBB | 0.01114 | 1.45E-10 | 331296 | IVNT |
| rs12964689 | chr18:21116998 | A | G | Leg fat percentage right | UKBB | 0.01163 | 3.99E-14 | 331296 | IVNT |
| rs1503526 | chr5:63020706 | T | C | Leg fat percentage right | UKBB | -0.01028 | 2.39E-11 | 331296 | IVNT |
| rs16903285 | chr5:87978252 | C | T | Leg fat percentage right | UKBB | 0.0176 | 1.92E-14 | 331296 | IVNT |
| rs2733287 | chr12:41880909 | C | A | Leg fat percentage right | UKBB | 0.008451 | 4.08E-08 | 331296 | IVNT |
| rs7334078 | chr13:99120484 | C | T | Leg fat percentage right | UKBB | -0.01114 | 6.81E-11 | 331296 | IVNT |
| rs8047395 | chr16:53798523 | A | G | Leg fat percentage right | UKBB | 0.02593 | 1.84E-63 | 331296 | IVNT |
| **SNP** | **Pos (hg19)** | **A1** | **A2** | **Trait** | **PMID** | **Beta** | **P** | **N** | **Unit** |
| rs901630 | chr6:98539519 | C | T | Leg fat percentage right | UKBB | 0.0143 | 1.06E-19 | 331296 | IVNT |
| rs9267677 | chr6:31892641 | C | T | Leg fat percentage right | UKBB | 0.0171 | 9.19E-11 | 331296 | IVNT |
| rs10811901 | chr9:23356935 | A | G | Leg fat-free mass left | UKBB | 0.01047 | 9.46E-11 | 331258 | IVNT |
| rs11611246 | chr12:939480 | G | T | Leg fat-free mass left | UKBB | -0.01456 | 9.41E-14 | 331258 | IVNT |
| rs11713193 | chr3:49924424 | A | G | Leg fat-free mass left | UKBB | 0.01463 | 5.39E-20 | 331258 | IVNT |
| rs12964689 | chr18:21116998 | A | G | Leg fat-free mass left | UKBB | 0.01322 | 1.36E-16 | 331258 | IVNT |
| rs16903285 | chr5:87978252 | C | T | Leg fat-free mass left | UKBB | 0.01551 | 8.67E-11 | 331258 | IVNT |
| rs2293605 | chr3:184044433 | T | C | Leg fat-free mass left | UKBB | -0.02006 | 3.41E-16 | 331258 | IVNT |
| rs2733287 | chr12:41880909 | C | A | Leg fat-free mass left | UKBB | 0.009087 | 1.38E-08 | 331258 | IVNT |
| rs329122 | chr5:133864599 | A | G | Leg fat-free mass left | UKBB | -0.008319 | 2.95E-07 | 331258 | IVNT |
| rs6449532 | chr5:60715446 | C | T | Leg fat-free mass left | UKBB | 0.01008 | 1.43E-09 | 331258 | IVNT |
| rs8047395 | chr16:53798523 | A | G | Leg fat-free mass left | UKBB | 0.03556 | 4.5E-109 | 331258 | IVNT |
| rs9816226 | chr3:185834499 | A | T | Leg fat-free mass left | UKBB | -0.01792 | 4.57E-18 | 331258 | IVNT |
| rs10811901 | chr9:23356935 | A | G | Leg fat-free mass right | UKBB | 0.01081 | 2.26E-11 | 331285 | IVNT |
| rs11611246 | chr12:939480 | G | T | Leg fat-free mass right | UKBB | -0.0144 | 1.69E-13 | 331285 | IVNT |
| rs11713193 | chr3:49924424 | A | G | Leg fat-free mass right | UKBB | 0.01352 | 2.68E-17 | 331285 | IVNT |
| rs12964689 | chr18:21116998 | A | G | Leg fat-free mass right | UKBB | 0.01241 | 8.39E-15 | 331285 | IVNT |
| rs16903285 | chr5:87978252 | C | T | Leg fat-free mass right | UKBB | 0.01386 | 6.68E-09 | 331285 | IVNT |
| rs2293605 | chr3:184044433 | T | C | Leg fat-free mass right | UKBB | -0.02085 | 2.2E-17 | 331285 | IVNT |
| rs329122 | chr5:133864599 | A | G | Leg fat-free mass right | UKBB | -0.008531 | 1.45E-07 | 331285 | IVNT |
| rs6449532 | chr5:60715446 | C | T | Leg fat-free mass right | UKBB | 0.01055 | 2.32E-10 | 331285 | IVNT |
| rs8047395 | chr16:53798523 | A | G | Leg fat-free mass right | UKBB | 0.03424 | 2.4E-101 | 331285 | IVNT |
| rs9816226 | chr3:185834499 | A | T | Leg fat-free mass right | UKBB | -0.01869 | 1.6E-19 | 331285 | IVNT |
| rs10811901 | chr9:23356935 | A | G | Leg predicted mass left | UKBB | 0.01032 | 1.33E-10 | 331253 | IVNT |
| rs11611246 | chr12:939480 | G | T | Leg predicted mass left | UKBB | -0.01436 | 1.36E-13 | 331253 | IVNT |
| rs11713193 | chr3:49924424 | A | G | Leg predicted mass left | UKBB | 0.01449 | 6.99E-20 | 331253 | IVNT |
| rs12964689 | chr18:21116998 | A | G | Leg predicted mass left | UKBB | 0.01308 | 1.8E-16 | 331253 | IVNT |
| rs16903285 | chr5:87978252 | C | T | Leg predicted mass left | UKBB | 0.01537 | 9.63E-11 | 331253 | IVNT |
| rs2293605 | chr3:184044433 | T | C | Leg predicted mass left | UKBB | -0.01981 | 5.01E-16 | 331253 | IVNT |
| rs2733287 | chr12:41880909 | C | A | Leg predicted mass left | UKBB | 0.009026 | 1.38E-08 | 331253 | IVNT |
| **SNP** | **Pos (hg19)** | **A1** | **A2** | **Trait** | **PMID** | **Beta** | **P** | **N** | **Unit** |
| rs329122 | chr5:133864599 | A | G | Leg predicted mass left | UKBB | -0.008297 | 2.64E-07 | 331253 | IVNT |
| rs6449532 | chr5:60715446 | C | T | Leg predicted mass left | UKBB | 0.01009 | 1.07E-09 | 331253 | IVNT |
| rs8047395 | chr16:53798523 | A | G | Leg predicted mass left | UKBB | 0.03526 | 1.1E-108 | 331253 | IVNT |
| rs9816226 | chr3:185834499 | A | T | Leg predicted mass left | UKBB | -0.01786 | 3.49E-18 | 331253 | IVNT |
| rs10811901 | chr9:23356935 | A | G | Leg predicted mass right | UKBB | 0.01074 | 2.27E-11 | 331285 | IVNT |
| rs11611246 | chr12:939480 | G | T | Leg predicted mass right | UKBB | -0.01435 | 1.4E-13 | 331285 | IVNT |
| rs11713193 | chr3:49924424 | A | G | Leg predicted mass right | UKBB | 0.01337 | 3.58E-17 | 331285 | IVNT |
| rs12964689 | chr18:21116998 | A | G | Leg predicted mass right | UKBB | 0.01234 | 7.93E-15 | 331285 | IVNT |
| rs16903285 | chr5:87978252 | C | T | Leg predicted mass right | UKBB | 0.01379 | 6.24E-09 | 331285 | IVNT |
| rs2293605 | chr3:184044433 | T | C | Leg predicted mass right | UKBB | -0.02064 | 2.86E-17 | 331285 | IVNT |
| rs329122 | chr5:133864599 | A | G | Leg predicted mass right | UKBB | -0.008382 | 1.98E-07 | 331285 | IVNT |
| rs6449532 | chr5:60715446 | C | T | Leg predicted mass right | UKBB | 0.01048 | 2.41E-10 | 331285 | IVNT |
| rs8047395 | chr16:53798523 | A | G | Leg predicted mass right | UKBB | 0.03402 | 2.5E-101 | 331285 | IVNT |
| rs9816226 | chr3:185834499 | A | T | Leg predicted mass right | UKBB | -0.01849 | 2.21E-19 | 331285 | IVNT |
| rs9816226 | chr3:185834499 | A | T | Obesity | 23563607 | NA | 2E-14 | - | - |
| rs8047395 | chr16:53798523 | A | G | Obesity body mass index | 21935397 | NA | 5.06E-10 | 5373 | - |
| rs8047395 | chr16:53798523 | A | G | Obesity class 1 | 23563607 | 0.18 | 7.1E-44 | 98091 | log OR |
| rs9816226 | chr3:185834499 | A | T | Obesity class 1 | 23563607 | -0.11 | 1E-11 | 96355 | log OR |
| rs8047395 | chr16:53798523 | A | G | Obesity class 2 | 23563607 | 0.24 | 7.9E-34 | 71945 | log OR |
| rs9816226 | chr3:185834499 | A | T | Obesity class 2 | 23563607 | -0.15 | 5.5E-09 | 65579 | log OR |
| rs8047395 | chr16:53798523 | A | G | Obesity class 3 | 23563607 | 0.29 | 2.2E-16 | 48475 | log OR |
| rs8047395 | chr16:53798523 | A | G | Obesity with early age of onset age 2 | 22484627 | NA | 4.08E-09 | 13848 | - |
| rs8047395 | chr16:53798523 | A | G | Overweight | 23563607 | 0.11 | 6.9E-32 | 157970 | log OR |
| rs9816226 | chr3:185834499 | A | T | Overweight | 23563607 | -0.07 | 2E-09 | 157400 | log OR |
| rs11611246 | chr12:939480 | G | T | Trunk fat mass | UKBB | -0.01936 | 9.24E-11 | 331093 | IVNT |
| rs11713193 | chr3:49924424 | A | G | Trunk fat mass | UKBB | 0.0244 | 1.76E-23 | 331093 | IVNT |
| rs11855853 | chr15:78012618 | C | T | Trunk fat mass | UKBB | 0.0161 | 5.55E-09 | 331093 | IVNT |
| rs12964689 | chr18:21116998 | A | G | Trunk fat mass | UKBB | 0.02204 | 2.03E-19 | 331093 | IVNT |
| rs1503526 | chr5:63020706 | T | C | Trunk fat mass | UKBB | -0.01568 | 1.49E-10 | 331093 | IVNT |
| rs16903285 | chr5:87978252 | C | T | Trunk fat mass | UKBB | 0.03086 | 3.13E-17 | 331093 | IVNT |
| **SNP** | **Pos (hg19)** | **A1** | **A2** | **Trait** | **PMID** | **Beta** | **P** | **N** | **Unit** |
| rs329122 | chr5:133864599 | A | G | Trunk fat mass | UKBB | -0.01311 | 1.27E-07 | 331093 | IVNT |
| rs7334078 | chr13:99120484 | C | T | Trunk fat mass | UKBB | -0.0159 | 4.62E-09 | 331093 | IVNT |
| rs8047395 | chr16:53798523 | A | G | Trunk fat mass | UKBB | 0.04837 | 1.06E-86 | 331093 | IVNT |
| rs901630 | chr6:98539519 | C | T | Trunk fat mass | UKBB | 0.01896 | 3.6E-14 | 331093 | IVNT |
| rs9816226 | chr3:185834499 | A | T | Trunk fat mass | UKBB | -0.01742 | 3.63E-08 | 331093 | IVNT |
| rs11713193 | chr3:49924424 | A | G | Trunk fat percentage | UKBB | 0.01873 | 4.6E-17 | 331113 | IVNT |
| rs11855853 | chr15:78012618 | C | T | Trunk fat percentage | UKBB | 0.01419 | 1.84E-08 | 331113 | IVNT |
| rs12964689 | chr18:21116998 | A | G | Trunk fat percentage | UKBB | 0.01596 | 8.86E-13 | 331113 | IVNT |
| rs1503526 | chr5:63020706 | T | C | Trunk fat percentage | UKBB | -0.01334 | 2.39E-09 | 331113 | IVNT |
| rs16903285 | chr5:87978252 | C | T | Trunk fat percentage | UKBB | 0.02645 | 2.25E-15 | 331113 | IVNT |
| rs329122 | chr5:133864599 | A | G | Trunk fat percentage | UKBB | -0.01002 | 9.67E-06 | 331113 | IVNT |
| rs7334078 | chr13:99120484 | C | T | Trunk fat percentage | UKBB | -0.01685 | 1.03E-11 | 331113 | IVNT |
| rs8047395 | chr16:53798523 | A | G | Trunk fat percentage | UKBB | 0.03541 | 2.07E-56 | 331113 | IVNT |
| rs901630 | chr6:98539519 | C | T | Trunk fat percentage | UKBB | 0.01845 | 6.96E-16 | 331113 | IVNT |
| rs10811901 | chr9:23356935 | A | G | Trunk fat-free mass | UKBB | 0.009088 | 4.24E-09 | 331030 | IVNT |
| rs11611246 | chr12:939480 | G | T | Trunk fat-free mass | UKBB | -0.01235 | 3.94E-11 | 331030 | IVNT |
| rs11713193 | chr3:49924424 | A | G | Trunk fat-free mass | UKBB | 0.01195 | 5.37E-15 | 331030 | IVNT |
| rs12964689 | chr18:21116998 | A | G | Trunk fat-free mass | UKBB | 0.01228 | 9.91E-16 | 331030 | IVNT |
| rs2293605 | chr3:184044433 | T | C | Trunk fat-free mass | UKBB | -0.01955 | 9.22E-17 | 331030 | IVNT |
| rs2733287 | chr12:41880909 | C | A | Trunk fat-free mass | UKBB | 0.00907 | 3.19E-09 | 331030 | IVNT |
| rs6449532 | chr5:60715446 | C | T | Trunk fat-free mass | UKBB | 0.009903 | 5.14E-10 | 331030 | IVNT |
| rs8047395 | chr16:53798523 | A | G | Trunk fat-free mass | UKBB | 0.0264 | 2.01E-66 | 331030 | IVNT |
| rs9816226 | chr3:185834499 | A | T | Trunk fat-free mass | UKBB | -0.01345 | 1.07E-11 | 331030 | IVNT |
| rs10811901 | chr9:23356935 | A | G | Trunk predicted mass | UKBB | 0.008914 | 7.45E-09 | 330995 | IVNT |
| rs11611246 | chr12:939480 | G | T | Trunk predicted mass | UKBB | -0.0123 | 4.03E-11 | 330995 | IVNT |
| rs11713193 | chr3:49924424 | A | G | Trunk predicted mass | UKBB | 0.01178 | 1.05E-14 | 330995 | IVNT |
| rs12964689 | chr18:21116998 | A | G | Trunk predicted mass | UKBB | 0.01211 | 2.02E-15 | 330995 | IVNT |
| rs2293605 | chr3:184044433 | T | C | Trunk predicted mass | UKBB | -0.01922 | 2.44E-16 | 330995 | IVNT |
| rs2733287 | chr12:41880909 | C | A | Trunk predicted mass | UKBB | 0.009111 | 2.4E-09 | 330995 | IVNT |
| rs6449532 | chr5:60715446 | C | T | Trunk predicted mass | UKBB | 0.009786 | 7.22E-10 | 330995 | IVNT |
| **SNP** | **Pos (hg19)** | **A1** | **A2** | **Trait** | **PMID** | **Beta** | **P** | **N** | **Unit** |
| rs8047395 | chr16:53798523 | A | G | Trunk predicted mass | UKBB | 0.0263 | 2.54E-66 | 330995 | IVNT |
| rs9816226 | chr3:185834499 | A | T | Trunk predicted mass | UKBB | -0.01333 | 1.41E-11 | 330995 | IVNT |
| rs11611246 | chr12:939480 | G | T | Waist circumference | UKBB | -0.01709 | 7.99E-11 | 336639 | IVNT |
| rs11713193 | chr3:49924424 | A | G | Waist circumference | UKBB | 0.02057 | 1.08E-21 | 336639 | IVNT |
| rs12964689 | chr18:21116998 | A | G | Waist circumference | UKBB | 0.0188 | 2.34E-18 | 336639 | IVNT |
| rs1452075 | chr3:62481063 | C | T | Waist circumference | UKBB | -0.01529 | 3.72E-10 | 336639 | IVNT |
| rs1503526 | chr5:63020706 | T | C | Waist circumference | UKBB | -0.01106 | 2.77E-07 | 336639 | IVNT |
| rs16903285 | chr5:87978252 | C | T | Waist circumference | UKBB | 0.02488 | 9.95E-15 | 336639 | IVNT |
| rs2733287 | chr12:41880909 | C | A | Waist circumference | UKBB | 0.01495 | 3.84E-12 | 336639 | IVNT |
| rs329122 | chr5:133864599 | A | G | Waist circumference | UKBB | -0.01076 | 8.29E-07 | 336639 | IVNT |
| rs8047395 | chr16:53798523 | A | G | Waist circumference | UKBB | 0.0463 | 2.5E-102 | 336639 | IVNT |
| rs8047395 | chr16:53798523 | A | G | Waist circumference | 25673412 | 0.063 | 5.2E-73 | 231450 | IVNT |
| rs8047395 | chr16:53798523 | A | G | Waist circumference | 25673412 | 0.059 | 5.4E-70 | 243787 | IVNT |
| rs8047395 | chr16:53798523 | A | G | Waist circumference | 23754948 | 0.06341 | 7.81E-32 | 85604 | IVNT |
| rs8047395 | chr16:53798523 | A | G | Waist circumference | 19557197 | NA | 1.5E-11 | 31373 | - |
| rs901630 | chr6:98539519 | C | T | Waist circumference | UKBB | 0.01251 | 1.34E-08 | 336639 | IVNT |
| rs9267677 | chr6:31892641 | C | T | Waist circumference | UKBB | 0.02154 | 5.29E-09 | 336639 | IVNT |
| rs9816226 | chr3:185834499 | A | T | Waist circumference | UKBB | -0.01947 | 2.59E-12 | 336639 | IVNT |
| rs9816226 | chr3:185834499 | A | T | Waist circumference | 25673412 | -0.028 | 2.5E-10 | 231702 | IVNT |
| rs9816226 | chr3:185834499 | A | T | Waist circumference | 25673412 | -0.026 | 8.5E-09 | 219366 | IVNT |
| rs8047395 | chr16:53798523 | A | G | Waist circumference in females | 25673412 | 0.059 | 1.4E-40 | 134233 | IVNT |
| rs8047395 | chr16:53798523 | A | G | Waist circumference in females | 23754948 | 0.055 | 1.03E-13 | 47310 | IVNT |
| rs8047395 | chr16:53798523 | A | G | Waist circumference in males | 25673412 | 0.062 | 9.9E-40 | 109704 | IVNT |
| rs8047395 | chr16:53798523 | A | G | Waist circumference in males | 23754948 | 0.073 | 3.73E-20 | 38295 | IVNT |
| rs8047395 | chr16:53798523 | A | G | Waist hip ratio | 25673412 | 0.036 | 9.7E-26 | 211565 | IVNT |
| rs8047395 | chr16:53798523 | A | G | Waist hip ratio | 25673412 | 0.034 | 8.6E-25 | 225957 | IVNT |
| rs8047395 | chr16:53798523 | A | G | Waist hip ratio | 23754948 | 0.03736 | 1.57E-11 | 77292 | IVNT |
| rs8047395 | chr16:53798523 | A | G | Waist hip ratio in females | 25673412 | 0.03 | 2.2E-11 | 124237 | IVNT |
| rs8047395 | chr16:53798523 | A | G | Waist hip ratio in males | 25673412 | 0.039 | 1.2E-15 | 99694 | IVNT |
| rs8047395 | chr16:53798523 | A | G | Waist hip ratio in males | 23754948 | 0.048 | 2.72E-09 | 34571 | IVNT |
| **SNP** | **Pos (hg19)** | **A1** | **A2** | **Trait** | **PMID** | **Beta** | **P** | **N** | **Unit** |
| rs10811901 | chr9:23356935 | A | G | Weight | UKBB | 0.01217 | 1.43E-08 | 336227 | IVNT |
| rs11611246 | chr12:939480 | G | T | Weight | UKBB | -0.02063 | 1.78E-15 | 336227 | IVNT |
| rs11713193 | chr3:49924424 | A | G | Weight | UKBB | 0.02468 | 2.68E-31 | 336227 | IVNT |
| rs11855853 | chr15:78012618 | C | T | Weight | UKBB | 0.01379 | 8.89E-09 | 336227 | IVNT |
| rs12964689 | chr18:21116998 | A | G | Weight | UKBB | 0.02064 | 2.28E-22 | 336227 | IVNT |
| rs1503526 | chr5:63020706 | T | C | Weight | UKBB | -0.01254 | 3.57E-09 | 336227 | IVNT |
| rs16903285 | chr5:87978252 | C | T | Weight | UKBB | 0.0241 | 2.93E-14 | 336227 | IVNT |
| rs2293605 | chr3:184044433 | T | C | Weight | UKBB | -0.01943 | 2.58E-09 | 336227 | IVNT |
| rs2733287 | chr12:41880909 | C | A | Weight | UKBB | 0.0145 | 8.68E-12 | 336227 | IVNT |
| rs329122 | chr5:133864599 | A | G | Weight | UKBB | -0.01293 | 1.95E-09 | 336227 | IVNT |
| rs8047395 | chr16:53798523 | A | G | Weight | UKBB | 0.04966 | 1.4E-120 | 336227 | IVNT |
| rs8047395 | chr16:53798523 | A | G | Weight | 23754948 | 0.0577 | 3.27E-34 | 125882 | IVNT |
| rs901630 | chr6:98539519 | C | T | Weight | UKBB | 0.01242 | 1.09E-08 | 336227 | IVNT |
| rs9816226 | chr3:185834499 | A | T | Weight | UKBB | -0.02186 | 1.68E-15 | 336227 | IVNT |
| rs9816226 | chr3:185834499 | A | T | Weight | 23754948 | -0.03859 | 3.63E-11 | 125910 | IVNT |
| rs8047395 | chr16:53798523 | A | G | Weight in females | 23754948 | 0.053 | 2.89E-16 | 67576 | IVNT |
| rs8047395 | chr16:53798523 | A | G | Weight in males | 23754948 | 0.063 | 3.15E-20 | 58305 | IVNT |
| rs11611246 | chr12:939480 | G | T | Whole body fat mass | UKBB | -0.02077 | 7.37E-13 | 330762 | IVNT |
| rs11713193 | chr3:49924424 | A | G | Whole body fat mass | UKBB | 0.02649 | 4.88E-29 | 330762 | IVNT |
| rs11855853 | chr15:78012618 | C | T | Whole body fat mass | UKBB | 0.01616 | 1.58E-09 | 330762 | IVNT |
| rs12964689 | chr18:21116998 | A | G | Whole body fat mass | UKBB | 0.0221 | 1.12E-20 | 330762 | IVNT |
| rs1503526 | chr5:63020706 | T | C | Whole body fat mass | UKBB | -0.01515 | 1.66E-10 | 330762 | IVNT |
| rs16903285 | chr5:87978252 | C | T | Whole body fat mass | UKBB | 0.03051 | 7.04E-18 | 330762 | IVNT |
| rs2733287 | chr12:41880909 | C | A | Whole body fat mass | UKBB | 0.01348 | 1.33E-08 | 330762 | IVNT |
| rs329122 | chr5:133864599 | A | G | Whole body fat mass | UKBB | -0.01379 | 9.78E-09 | 330762 | IVNT |
| rs7334078 | chr13:99120484 | C | T | Whole body fat mass | UKBB | -0.01562 | 2.87E-09 | 330762 | IVNT |
| rs8047395 | chr16:53798523 | A | G | Whole body fat mass | UKBB | 0.05046 | 3.7E-100 | 330762 | IVNT |
| rs901630 | chr6:98539519 | C | T | Whole body fat mass | UKBB | 0.0195 | 9.34E-16 | 330762 | IVNT |
| rs9816226 | chr3:185834499 | A | T | Whole body fat mass | UKBB | -0.02012 | 5.22E-11 | 330762 | IVNT |
| rs10811901 | chr9:23356935 | A | G | Whole body fat-free mass | UKBB | 0.00988 | 2E-10 | 331291 | IVNT |
| **SNP** | **Pos (hg19)** | **A1** | **A2** | **Trait** | **PMID** | **Beta** | **P** | **N** | **Unit** |
| rs11611246 | chr12:939480 | G | T | Whole body fat-free mass | UKBB | -0.01378 | 2.09E-13 | 331291 | IVNT |
| rs11713193 | chr3:49924424 | A | G | Whole body fat-free mass | UKBB | 0.01358 | 9.11E-19 | 331291 | IVNT |
| rs12964689 | chr18:21116998 | A | G | Whole body fat-free mass | UKBB | 0.01311 | 1.39E-17 | 331291 | IVNT |
| rs2293605 | chr3:184044433 | T | C | Whole body fat-free mass | UKBB | -0.02029 | 8.51E-18 | 331291 | IVNT |
| rs2733287 | chr12:41880909 | C | A | Whole body fat-free mass | UKBB | 0.009245 | 1.83E-09 | 331291 | IVNT |
| rs329122 | chr5:133864599 | A | G | Whole body fat-free mass | UKBB | -0.007556 | 1.25E-06 | 331291 | IVNT |
| rs6449532 | chr5:60715446 | C | T | Whole body fat-free mass | UKBB | 0.009925 | 5.51E-10 | 331291 | IVNT |
| rs8047395 | chr16:53798523 | A | G | Whole body fat-free mass | UKBB | 0.03084 | 2.87E-89 | 331291 | IVNT |
| rs9816226 | chr3:185834499 | A | T | Whole body fat-free mass | UKBB | -0.01594 | 1.04E-15 | 331291 | IVNT |
| rs10811901 | chr9:23356935 | A | G | Whole body water mass | UKBB | 0.01 | 1.26E-10 | 331315 | IVNT |
| rs11611246 | chr12:939480 | G | T | Whole body water mass | UKBB | -0.01394 | 1.19E-13 | 331315 | IVNT |
| rs11713193 | chr3:49924424 | A | G | Whole body water mass | UKBB | 0.01378 | 3.16E-19 | 331315 | IVNT |
| rs12964689 | chr18:21116998 | A | G | Whole body water mass | UKBB | 0.0132 | 9.66E-18 | 331315 | IVNT |
| rs2293605 | chr3:184044433 | T | C | Whole body water mass | UKBB | -0.02029 | 9.45E-18 | 331315 | IVNT |
| rs2733287 | chr12:41880909 | C | A | Whole body water mass | UKBB | 0.009243 | 1.95E-09 | 331315 | IVNT |
| rs329122 | chr5:133864599 | A | G | Whole body water mass | UKBB | -0.007623 | 1.04E-06 | 331315 | IVNT |
| rs6449532 | chr5:60715446 | C | T | Whole body water mass | UKBB | 0.01014 | 2.48E-10 | 331315 | IVNT |
| rs8047395 | chr16:53798523 | A | G | Whole body water mass | UKBB | 0.03121 | 3.86E-91 | 331315 | IVNT |
| rs9816226 | chr3:185834499 | A | T | Whole body water mass | UKBB | -0.01585 | 1.63E-15 | 331315 | IVNT |
| **Associations of outlying SNPs with education traits** | | | | | | | | | |
| **SNP** | **Pos (hg19)** | **A1** | **A2** | **Trait** | **PMID** | **Beta** | **P** | **N** | **Unit** |
| rs10811901 | chr9:23356935 | A | G | Qualifications: A levels or as levels or equivalent | UKBB | 0.006647 | 1.72E-09 | 334070 | risk diff |
| rs11713193 | chr3:49924424 | A | G | Qualifications: A levels or as levels or equivalent | UKBB | -0.01057 | 3.35E-22 | 334070 | risk diff |
| rs901630 | chr6:98539519 | C | T | Qualifications: A levels or as levels or equivalent | UKBB | -0.008574 | 1.69E-14 | 334070 | risk diff |
| rs10811901 | chr9:23356935 | A | G | Qualifications: college or university degree | UKBB | 0.01024 | 5.91E-19 | 334070 | risk diff |
| rs11713193 | chr3:49924424 | A | G | Qualifications: college or university degree | UKBB | -0.01369 | 2.53E-33 | 334070 | risk diff |
|  |  |  |  |  |  |  |  |  |  |
| **SNP** | **Pos (hg19)** | **A1** | **A2** | **Trait** | **PMID** | **Beta** | **P** | **N** | **Unit** |
| rs1402025 | chr5:113987898 | T | C | Qualifications: college or university degree | UKBB | 0.008666 | 1.8E-10 | 334070 | risk diff |
| rs1503526 | chr5:63020706 | T | C | Qualifications: college or university degree | UKBB | 0.007172 | 3.08E-10 | 334070 | risk diff |
| rs16903285 | chr5:87978252 | C | T | Qualifications: college or university degree | UKBB | 0.01092 | 1.36E-10 | 334070 | risk diff |
| rs329122 | chr5:133864599 | A | G | Qualifications: college or university degree | UKBB | 0.0061 | 1.29E-07 | 334070 | risk diff |
| rs6449532 | chr5:60715446 | C | T | Qualifications: college or university degree | UKBB | 0.008129 | 7.15E-12 | 334070 | risk diff |
| rs901630 | chr6:98539519 | C | T | Qualifications: college or university degree | UKBB | -0.01415 | 6.86E-34 | 334070 | risk diff |
| rs903959 | chr8:142630782 | A | T | Qualifications: college or university degree | UKBB | -0.007821 | 1.86E-11 | 334070 | risk diff |
| rs901630 | chr6:98539519 | C | T | Qualifications: CSEs or equivalent | UKBB | 0.005341 | 3.32E-10 | 334070 | risk diff |
| rs11713193 | chr3:49924424 | A | G | Qualifications: none | UKBB | 0.006492 | 1.98E-12 | 334070 | risk diff |
| rs1503526 | chr5:63020706 | T | C | Qualifications: none | UKBB | -0.004131 | 7.76E-06 | 334070 | risk diff |
| rs4916229 | chr1:171443368 | C | G | Qualifications: none | UKBB | -0.006993 | 8.35E-06 | 334070 | risk diff |
| rs6449532 | chr5:60715446 | C | T | Qualifications: none | UKBB | -0.005527 | 8.98E-09 | 334070 | risk diff |
| rs901630 | chr6:98539519 | C | T | Qualifications: none | UKBB | 0.007384 | 5.63E-15 | 334070 | risk diff |
| rs4916229 | chr1:171443368 | C | G | Qualifications: other professional qualifications | UKBB | 0.009066 | 1.63E-06 | 334070 | risk diff |
| rs10811901 | chr9:23356935 | A | G | Years of educational attainment | 27225129 | 0.017 | 9.52E-12 | 328917 | years |
| rs11713193 | chr3:49924424 | A | G | Years of educational attainment | 27225129 | -0.022 | 6.86E-19 | 328917 | years |
| rs12964689 | chr18:21116998 | A | G | Years of educational attainment | 27225129 | -0.014 | 1.19E-08 | 328917 | years |
| rs1402025 | chr5:113987898 | T | C | Years of educational attainment | 27225129 | 0.018 | 1.51E-09 | 328917 | years |
| rs1503526 | chr5:63020706 | T | C | Years of educational attainment | 27225129 | 0.011 | 3.87E-06 | 328917 | years |
| rs329122 | chr5:133864599 | A | G | Years of educational attainment | 27225129 | 0.012 | 7.35E-07 | 328917 | years |
| rs4916229 | chr1:171443368 | C | G | Years of educational attainment | 27225129 | 0.021 | 6.03E-07 | 328917 | years |
| rs901630 | chr6:98539519 | C | T | Years of educational attainment | 27225129 | -0.024 | 1.58E-20 | 328917 | years |
| rs903959 | chr8:142630782 | A | T | Years of educational attainment | 27225129 | -0.016 | 3.35E-10 | 328917 | years |
| rs9965170 | chr18:44788274 | A | G | Years of educational attainment | 27225129 | 0.014 | 2.38E-08 | 328917 | years |
|  |  |  |  |  |  |  |  |  |  |
| **SNP** | **Pos (hg19)** | **A1** | **A2** | **Trait** | **PMID** | **Beta** | **P** | **N** | **Unit** |
| rs11713193 | chr3:49924424 | A | G | Years of educational attainment in females | 27225129 | -0.024 | 6.76E-13 | 181443 | years |
| rs901630 | chr6:98539519 | C | T | Years of educational attainment in females | 27225129 | -0.025 | 5.72E-13 | 181443 | years |
| rs903959 | chr8:142630782 | A | T | Years of educational attainment in females | 27225129 | -0.021 | 3.57E-10 | 181443 | years |
| rs11713193 | chr3:49924424 | A | G | Years of educational attainment in males | 27225129 | -0.022 | 1.31E-09 | 147474 | years |
| rs1402025 | chr5:113987898 | T | C | Years of educational attainment in males | 27225129 | 0.024 | 3.49E-08 | 147474 | years |
| rs901630 | chr6:98539519 | C | T | Years of educational attainment in males | 27225129 | -0.025 | 6.69E-11 | 147474 | years |
| **Associations of outlying SNPs with other traits** | | | | | | | | | |
| **SNP** | **Pos (hg19)** | **A1** | **A2** | **Trait** | **PMID** | **Beta** | **P** | **N** | **Unit** |
| rs9267677 | chr6:31892641 | C | T | Advanced age related macular degeneration geographic atrophy | 23455636 | NA | 9.48E-11 | 59494 | - |
| rs11713193 | chr3:49924424 | A | G | Age at first live birth | UKBB | -0.03225 | 2.64E-16 | 123846 | IVNT |
| rs329122 | chr5:133864599 | A | G | Age at first live birth | UKBB | 0.01796 | 6.89E-06 | 123846 | IVNT |
| rs11713193 | chr3:49924424 | A | G | Age at last live birth | UKBB | -0.02977 | 5.13E-14 | 123676 | IVNT |
| rs329122 | chr5:133864599 | A | G | Age at menarche | 25231870 | 0.03 | 1E-09 | 182416 | years |
| rs329122 | chr5:133864599 | A | G | Age at menarche | UKBB | 0.01411 | 1.95E-08 | 176008 | - |
| rs8047395 | chr16:53798523 | A | G | Age at menarche | UKBB | -0.02636 | 1.92E-26 | 176008 | - |
| rs8047395 | chr16:53798523 | A | G | Age at menarche | 25231870 | -0.033 | 1.2E-10 | 182416 | years |
| rs9965170 | chr18:44788274 | A | G | Age at menarche | UKBB | 0.02173 | 4.79E-18 | 176008 | - |
| rs9965170 | chr18:44788274 | A | G | Age at menarche | 25231870 | 0.04 | 2.7E-16 | 182416 | years |
| rs11713193 | chr3:49924424 | A | G | Age completed full time education | UKBB | -0.01515 | 4.17E-10 | 226899 | - |
| rs11713193 | chr3:49924424 | A | G | Age first birth | 27798627 | NA | 5.67E-14 | 213067 | years |
| rs329122 | chr5:133864599 | A | G | Age first birth | 27798627 | NA | 1.77E-06 | 213067 | years |
| rs11713193 | chr3:49924424 | A | G | Age first birth female | 27798627 | NA | 4.4E-09 | 163522 | years |
| rs9267677 | chr6:31892641 | C | T | Age-related macular degeneration | 26691988 | NA | 7.1E-14 | 33976 | log OR |
| rs11713193 | chr3:49924424 | A | G | Alcohol intake frequency | UKBB | 0.02141 | 1.64E-09 | 336965 | - |
| rs12964689 | chr18:21116998 | A | G | Alcohol intake frequency | UKBB | 0.02901 | 3.14E-16 | 336965 | - |
| **SNP** | **Pos (hg19)** | **A1** | **A2** | **Trait** | **PMID** | **Beta** | **P** | **N** | **Unit** |
| rs4916229 | chr1:171443368 | C | G | Alcohol intake frequency | UKBB | -0.03017 | 5.86E-07 | 336965 | - |
| rs8047395 | chr16:53798523 | A | G | Alcohol intake frequency | UKBB | 0.01651 | 3.56E-06 | 336965 | - |
| rs901630 | chr6:98539519 | C | T | Alcohol intake frequency | UKBB | 0.02194 | 1.63E-09 | 336965 | - |
| rs903959 | chr8:142630782 | A | T | Alcohol intake frequency | UKBB | 0.02166 | 2.49E-09 | 336965 | - |
| rs12964689 | chr18:21116998 | A | G | Average weekly red wine intake | UKBB | -0.01494 | 5.66E-10 | 241008 | IVNT |
| rs8047395 | chr16:53798523 | A | G | Average weekly spirits intake | UKBB | 0.01164 | 5.41E-06 | 240658 | - |
| rs10811901 | chr9:23356935 | A | G | Basal metabolic rate | UKBB | 0.01046 | 1.36E-10 | 331307 | IVNT |
| rs11611246 | chr12:939480 | G | T | Basal metabolic rate | UKBB | -0.01499 | 2.54E-14 | 331307 | IVNT |
| rs11713193 | chr3:49924424 | A | G | Basal metabolic rate | UKBB | 0.01584 | 7.54E-23 | 331307 | IVNT |
| rs12964689 | chr18:21116998 | A | G | Basal metabolic rate | UKBB | 0.01479 | 4.25E-20 | 331307 | IVNT |
| rs1503526 | chr5:63020706 | T | C | Basal metabolic rate | UKBB | -0.007147 | 9.24E-06 | 331307 | IVNT |
| rs16903285 | chr5:87978252 | C | T | Basal metabolic rate | UKBB | 0.01394 | 7.03E-09 | 331307 | IVNT |
| rs2293605 | chr3:184044433 | T | C | Basal metabolic rate | UKBB | -0.02002 | 6.17E-16 | 331307 | IVNT |
| rs2733287 | chr12:41880909 | C | A | Basal metabolic rate | UKBB | 0.01024 | 2.18E-10 | 331307 | IVNT |
| rs329122 | chr5:133864599 | A | G | Basal metabolic rate | UKBB | -0.00864 | 1.24E-07 | 331307 | IVNT |
| rs6449532 | chr5:60715446 | C | T | Basal metabolic rate | UKBB | 0.009915 | 3.41E-09 | 331307 | IVNT |
| rs8047395 | chr16:53798523 | A | G | Basal metabolic rate | UKBB | 0.03446 | 3.7E-101 | 331307 | IVNT |
| rs9816226 | chr3:185834499 | A | T | Basal metabolic rate | UKBB | -0.01695 | 4.1E-16 | 331307 | IVNT |
| rs10840606 | chr11:2234690 | A | G | Birth weight | UKBB | -0.02636 | 2.56E-10 | 193063 | IVNT |
| rs329122 | chr5:133864599 | A | G | Birth weight | UKBB | 0.01436 | 7.7E-06 | 193063 | IVNT |
| rs329122 | chr5:133864599 | A | G | Birth weight of first child | UKBB | 0.0212 | 2.62E-06 | 145558 | - |
| rs11611246 | chr12:939480 | G | T | Comparative body size at age 10 | UKBB | -0.01538 | 3.7E-14 | 331693 | - |
| rs6449532 | chr5:60715446 | C | T | Comparative body size at age 10 | UKBB | 0.01033 | 2.41E-09 | 331693 | - |
| rs8047395 | chr16:53798523 | A | G | Comparative body size at age 10 | UKBB | 0.03948 | 3.2E-124 | 331693 | - |
| rs12964689 | chr18:21116998 | A | G | Comparative height size at age 10 | UKBB | 0.01183 | 9.67E-13 | 332021 | - |
| rs8047395 | chr16:53798523 | A | G | Comparative height size at age 10 | UKBB | 0.008078 | 1.17E-06 | 332021 | - |
| rs4916229 | chr1:171443368 | C | G | Creatinine in urine | UKBB | -0.02164 | 3.12E-08 | 327525 | IVNT |
| rs11713193 | chr3:49924424 | A | G | Crohns disease | 23128233 | NA | 6.1E-10 | 14342 | log OR |
| rs9267677 | chr6:31892641 | C | T | Crohns disease | 26192919 | 0.2221 | 2.58E-10 | 20883 | log OR |
| rs329122 | chr5:133864599 | A | G | Current tobacco smoking | UKBB | -0.006546 | 1.05E-06 | 337030 | - |
| **SNP** | **Pos (hg19)** | **A1** | **A2** | **Trait** | **PMID** | **Beta** | **P** | **N** | **Unit** |
| rs8047395 | chr16:53798523 | A | G | Diabetes diagnosed by doctor | UKBB | 0.004025 | 1.24E-14 | 336473 | risk diff |
| rs11713193 | chr3:49924424 | A | G | Diastolic blood pressure | UKBB | 0.01354 | 3.78E-08 | 317756 | IVNT |
| rs16903285 | chr5:87978252 | C | T | Diastolic blood pressure | UKBB | 0.02072 | 1.75E-08 | 317756 | IVNT |
| rs329122 | chr5:133864599 | A | G | Diastolic blood pressure | UKBB | -0.01108 | 9.26E-06 | 317756 | IVNT |
| rs11611246 | chr12:939480 | G | T | Eosinophil count | 27863252 | -0.02835 | 8.39E-11 | 173480 | - |
| rs11611246 | chr12:939480 | G | T | Eosinophil percentage of granulocytes | 27863252 | -0.02758 | 3.07E-10 | 173480 | - |
| rs11611246 | chr12:939480 | G | T | Eosinophil percentage of white cells | 27863252 | -0.02847 | 6.79E-11 | 173480 | - |
| rs11713193 | chr3:49924424 | A | G | Fluid intelligence score | UKBB | -0.06502 | 4.81E-13 | 108818 | - |
| rs901630 | chr6:98539519 | C | T | Fluid intelligence score | UKBB | -0.06372 | 4.69E-12 | 108818 | - |
| rs4916229 | chr1:171443368 | C | G | Frequency of tiredness or lethargy in last 2 weeks | UKBB | -0.01721 | 6.41E-07 | 327528 | - |
| rs9267677 | chr6:31892641 | C | T | Geographic atrophy | 23455636 | NA | 9.48E-11 | 48433 | log OR |
| rs8047395 | chr16:53798523 | A | G | Getting up in morning | UKBB | 0.008403 | 0.000007 | 336501 | - |
| rs12964689 | chr18:21116998 | A | G | Granulocyte count | 27863252 | -0.02003 | 2.37E-08 | 173480 | - |
| rs9267677 | chr6:31892641 | C | T | Granulocyte count | 27863252 | 0.03439 | 1.68E-08 | 173480 | - |
| rs8047395 | chr16:53798523 | A | G | HbA1c | 28898252 | 0.01 | 3.27E-08 | 123665 | % |
| rs8047395 | chr16:53798523 | A | G | HDL cholesterol | 20686565 | NA | 5.62E-06 | 100184 | - |
| rs11713193 | chr3:49924424 | A | G | Heel bone mineral density | UKBB | 0.02135 | 6.45E-12 | 194398 | IVNT |
| rs8047395 | chr16:53798523 | A | G | Heel bone mineral density | UKBB | 0.01857 | 2.79E-09 | 194398 | IVNT |
| rs12964689 | chr18:21116998 | A | G | Height | UKBB | 0.01005 | 5.73E-09 | 336474 | IVNT |
| rs8047395 | chr16:53798523 | A | G | High density lipoprotein | 24097068 | -0.0187 | 2.88E-08 | 185511 | IVNT |
| rs8047395 | chr16:53798523 | A | G | High density lipoprotein | 20686565 | -0.0214 | 5.62E-06 | 99900 | Z-score |
| rs9267677 | chr6:31892641 | C | T | IgA deficiency | 27723758 | 0.6496 | 1.5E-17 | 6487 | log OR |
| rs1503526 | chr5:63020706 | T | C | Illnesses of father: chronic bronchitis or emphysema | UKBB | -0.003575 | 9.9E-06 | 294680 | risk diff |
| rs8047395 | chr16:53798523 | A | G | Illnesses of father: diabetes | UKBB | 0.003551 | 2.8E-06 | 293407 | risk diff |
| rs8047395 | chr16:53798523 | A | G | Illnesses of siblings: high blood pressure | UKBB | 0.005355 | 1.38E-06 | 262361 | risk diff |
| rs11713193 | chr3:49924424 | A | G | Inflammatory bowel disease | 26192919 | -0.097 | 7.15E-09 | 34652 | log OR |
| rs901630 | chr6:98539519 | C | T | Intelligence multi trait analysis | 29326435 | -0.03324 | 1E-28 | - | unit increase |
| **SNP** | **Pos (hg19)** | **A1** | **A2** | **Trait** | **PMID** | **Beta** | **P** | **N** | **Unit** |
| rs16903285 | chr5:87978252 | C | T | Irritability | UKBB | 0.009395 | 1.75E-08 | 322668 | risk diff |
| rs11713193 | chr3:49924424 | A | G | Job involves heavy manual or physical work | UKBB | 0.02281 | 5.72E-16 | 190643 | - |
| rs901630 | chr6:98539519 | C | T | Job involves heavy manual or physical work | UKBB | 0.02074 | 6.57E-13 | 190643 | - |
| rs11713193 | chr3:49924424 | A | G | Job involves mainly walking or standing | UKBB | 0.02344 | 5.98E-11 | 190606 | IVNT |
| rs901630 | chr6:98539519 | C | T | Job involves mainly walking or standing | UKBB | 0.0278 | 3.47E-14 | 190606 | IVNT |
| rs10840606 | chr11:2234690 | A | G | Malignant neoplasm of prostate | UKBB | -0.001732 | 4E-11 | 337199 | risk diff |
| rs8047395 | chr16:53798523 | A | G | Medication for cholesterol, blood pressure or diabetes: none of the above | UKBB | -0.007957 | 3.05E-06 | 154702 | risk diff |
| rs11713193 | chr3:49924424 | A | G | Miserableness | UKBB | 0.006884 | 1.06E-08 | 331856 | risk diff |
| rs8047395 | chr16:53798523 | A | G | Morning or evening person | UKBB | -0.01683 | 2.07E-12 | 301143 | - |
| rs9267677 | chr6:31892641 | C | T | Myeloid white cell count | 27863252 | 0.0335 | 4.19E-08 | 173480 | - |
| rs9965170 | chr18:44788274 | A | G | Nap during day | UKBB | -0.01368 | 6.41E-21 | 337074 | - |
| rs12964689 | chr18:21116998 | A | G | Neutrophil count | 27863252 | -0.02022 | 1.61E-08 | 173480 | - |
| rs11611246 | chr12:939480 | G | T | Neutrophil percentage of granulocytes | 27863252 | 0.02605 | 2.78E-09 | 173480 | - |
| rs8047395 | chr16:53798523 | A | G | Nonsyndromic striae distensae stretch marks | 23633020 | NA | 5.05E-06 | 33930 | - |
| rs901630 | chr6:98539519 | C | T | Number of days or week walked 10+ minutes | UKBB | 0.03044 | 3.58E-10 | 331654 | - |
| rs329122 | chr5:133864599 | A | G | Number of operations | UKBB | -0.009778 | 4.13E-07 | 337159 | - |
| rs11713193 | chr3:49924424 | A | G | Number of treatments or medications taken | UKBB | 0.01245 | 1.59E-10 | 337159 | - |
| rs8047395 | chr16:53798523 | A | G | Number of treatments or medications taken | UKBB | 0.00871 | 8.17E-06 | 337159 | - |
| rs11713193 | chr3:49924424 | A | G | Overall health rating | UKBB | 0.01251 | 1.31E-12 | 336020 | - |
| rs901630 | chr6:98539519 | C | T | Overall health rating | UKBB | 0.01198 | 3.21E-11 | 336020 | - |
| rs329122 | chr5:133864599 | A | G | Platelet distribution width | 27863252 | -0.0183 | 5.3E-07 | 173480 | - |
| rs16903285 | chr5:87978252 | C | T | Plateletcrit | 27863252 | 0.03334 | 8.9E-10 | 173480 | - |
| rs2293605 | chr3:184044433 | T | C | Plateletcrit | 27863252 | -0.03372 | 1.93E-09 | 173480 | - |
| rs9267677 | chr6:31892641 | C | T | Primary sclerosing cholangitis | 27992413 | -0.3736 | 3.6E-12 | 14890 | log OR |
| **SNP** | **Pos (hg19)** | **A1** | **A2** | **Trait** | **PMID** | **Beta** | **P** | **N** | **Unit** |
| rs10840606 | chr11:2234690 | A | G | Prostate cancer | 19767753 | NA | 1.5E-11 | 3748 | - |
| rs9267677 | chr6:31892641 | C | T | Psoriasis | UKBB | 0.001026 | 1.24E-14 | 337199 | risk diff |
| rs16903285 | chr5:87978252 | C | T | Platelet count | 27863252 | 0.03337 | 7.69E-10 | 173480 | - |
| rs8047395 | chr16:53798523 | A | G | Relative age of first facial hair | UKBB | -0.008487 | 9.81E-08 | 151113 | - |
| rs8047395 | chr16:53798523 | A | G | Relative age voice broke | UKBB | -0.005663 | 1.85E-06 | 144645 | - |
| rs9267677 | chr6:31892641 | C | T | Rheumatoid arthritis | 24390342 | -0.3784 | 3.8E-39 | 80799 | log OR |
| rs9267677 | chr6:31892641 | C | T | Rheumatoid arthritis | 24390342 | -0.3646 | 2.2E-35 | 58284 | log OR |
| rs9267677 | chr6:31892641 | C | T | Rheumatoid arthritis | 20453842 | NA | 8.94E-15 | 25708 | - |
| rs9267677 | chr6:31892641 | C | T | Rheumatoid arthritis | 20453842 | -0.3425 | 8.94E-15 | 25704 | log OR |
| rs8047395 | chr16:53798523 | A | G | Self-reported breast cancer | UKBB | -0.00182 | 3.27E-07 | 337159 | risk diff |
| rs8047395 | chr16:53798523 | A | G | Self-reported diabetes | UKBB | 0.003339 | 1.73E-12 | 337159 | risk diff |
| rs1402025 | chr5:113987898 | T | C | Self-reported hypertension | UKBB | -0.007716 | 1.3E-09 | 337159 | risk diff |
| rs8047395 | chr16:53798523 | A | G | Self-reported hypertension | UKBB | 0.006877 | 1.21E-10 | 337159 | risk diff |
| s10840606 | chr11:2234690 | A | G | Self-reported prostate cancer | UKBB | -0.002055 | 7.1E-14 | 337159 | risk diff |
| rs9267677 | chr6:31892641 | C | T | Self-reported psoriasis | UKBB | 0.0144 | 8.9E-230 | 337159 | risk diff |
| rs9267677 | chr6:31892641 | C | T | Self-reported psoriatic arthropathy | UKBB | 0.001876 | 1.42E-24 | 337159 | risk diff |
| rs10811901 | chr9:23356935 | A | G | Sitting height | UKBB | 0.01109 | 5.09E-09 | 336172 | IVNT |
| rs8047395 | chr16:53798523 | A | G | Sleep duration | UKBB | -0.008672 | 4.01E-06 | 335410 | - |
| rs329122 | chr5:133864599 | A | G | Smoking status: current | UKBB | -0.003546 | 1.91E-06 | 336024 | risk diff |
| rs8047395 | chr16:53798523 | A | G | Snoring | UKBB | -0.006665 | 2.76E-08 | 314449 | risk diff |
| rs4916229 | chr1:171443368 | C | G | Sodium in urine | UKBB | -0.01944 | 1.32E-06 | 326831 | IVNT |
| rs8047395 | chr16:53798523 | A | G | Sodium in urine | UKBB | 0.0161 | 1.11E-11 | 326831 | IVNT |
| rs901630 | chr6:98539519 | C | T | Sodium in urine | UKBB | 0.01399 | 7.5E-09 | 326831 | IVNT |
| rs12964689 | chr18:21116998 | A | G | Sum basophil neutrophil counts | 27863252 | -0.01978 | 3.47E-08 | 173480 | - |
| rs11611246 | chr12:939480 | G | T | Sum eosinophil basophil counts | 27863252 | -0.02581 | 3.52E-09 | 173480 | - |
| rs12964689 | chr18:21116998 | A | G | Sum neutrophil eosinophil counts | 27863252 | -0.02026 | 1.57E-08 | 173480 | - |
| rs9267677 | chr6:31892641 | C | T | Sum neutrophil eosinophil counts | 27863252 | 0.03386 | 2.63E-08 | 173480 | - |
| rs8047395 | chr16:53798523 | A | G | Systolic blood pressure | UKBB | 0.01092 | 9.59E-06 | 317754 | IVNT |
| rs11713193 | chr3:49924424 | A | G | Taking other prescription medications | UKBB | 0.007812 | 1.33E-10 | 336330 | risk diff |
| rs12705977 | chr7:114332246 | G | T | Tense or highly strung | UKBB | -0.005141 | 3.53E-08 | 327232 | risk diff |
| **SNP** | **Pos (hg19)** | **A1** | **A2** | **Trait** | **PMID** | **Beta** | **P** | **N** | **Unit** |
| rs10811901 | chr9:23356935 | A | G | Time spent using computer | UKBB | 0.0138 | 2.15E-10 | 261987 | - |
| rs901630 | chr6:98539519 | C | T | Time spent using computer | UKBB | -0.01445 | 5.3E-11 | 261987 | - |
| rs10811901 | chr9:23356935 | A | G | Time spent watching television | UKBB | -0.01173 | 5.42E-10 | 319740 | - |
| rs11713193 | chr3:49924424 | A | G | Time spent watching television | UKBB | 0.01887 | 5.75E-24 | 319740 | - |
| rs901630 | chr6:98539519 | C | T | Time spent watching television | UKBB | 0.01243 | 8.36E-11 | 319740 | - |
| rs8047395 | chr16:53798523 | A | G | Treatment with bendroflumethiazide | UKBB | 0.002526 | 7.67E-06 | 337159 | risk diff |
| rs8047395 | chr16:53798523 | A | G | Treatment with blood pressure medication | UKBB | 0.006135 | 1.27E-06 | 180203 | risk diff |
| rs9267677 | chr6:31892641 | C | T | Treatment with calcipotriol | UKBB | 0.000724 | 6.57E-13 | 337159 | risk diff |
| rs9267677 | chr6:31892641 | C | T | Treatment with dovobet ointment | UKBB | 0.001213 | 1.11E-17 | 337159 | risk diff |
| rs9267677 | chr6:31892641 | C | T | Treatment with dovonex 50micrograms or g cream | UKBB | 0.000743 | 1.88E-13 | 337159 | risk diff |
| rs8047395 | chr16:53798523 | A | G | Treatment with metformin | UKBB | 0.002514 | 3.68E-11 | 337159 | risk diff |
| rs8047395 | chr16:53798523 | A | G | Triglycerides | 24097068 | 0.0168 | 2.24E-06 | 176178 | IVNT |
| rs329122 | chr5:133864599 | A | G | Type 2 diabetes | 28869590 | 0.03922 | 3E-09 | - | log OR |
| rs329122 | chr5:133864599 | A | G | Type II diabetes | 24509480 | 0.05827 | 8.3E-06 | 110452 | log OR |
| rs8047395 | chr16:53798523 | A | G | Type II diabetes | 26551672 | 0.1213 | 8.1E-22 | 84780 | log OR |
| rs8047395 | chr16:53798523 | A | G | Type II diabetes | 28566273 | 0.1 | 3.2E-16 | 159208 | log OR |
| rs8047395 | chr16:53798523 | A | G | Type II diabetes | 22885922 | 0.1044 | 7.3E-08 | 63390 | log OR |
| rs8047395 | chr16:53798523 | A | G | Type II diabetes | 24509480 | 0.07696 | 1.8E-07 | 110452 | log OR |
| rs11713193 | chr3:49924424 | A | G | Ulcerative colitis | 23128233 | NA | 3.91E-08 | 20672 | log OR |
| rs11713193 | chr3:49924424 | A | G | Usual walking pace | UKBB | -0.009446 | 1.92E-10 | 335349 | - |
| rs12964689 | chr18:21116998 | A | G | Usual walking pace | UKBB | -0.008194 | 3.37E-08 | 335349 | - |
| rs8047395 | chr16:53798523 | A | G | Usual walking pace | UKBB | -0.007751 | 1.88E-07 | 335349 | - |
| rs12705977 | chr7:114332246 | G | T | Vascular or heart problems diagnosed by doctor: high blood pressure | UKBB | -0.006017 | 2.77E-08 | 336683 | risk diff |
| rs1402025 | chr5:113987898 | T | C | Vascular or heart problems diagnosed by doctor: high blood pressure | UKBB | -0.007418 | 8.73E-09 | 336683 | risk diff |
| rs8047395 | chr16:53798523 | A | G | Vascular or heart problems diagnosed by doctor: high blood pressure | UKBB | 0.00677 | 4.06E-10 | 336683 | risk diff |
| rs1402025 | chr5:113987898 | T | C | Vascular or heart problems diagnosed by doctor: none of the above | UKBB | 0.007805 | 3.75E-09 | 336683 | risk diff |
| **SNP** | **Pos (hg19)** | **A1** | **A2** | **Trait** | **PMID** | **Beta** | **P** | **N** | **Unit** |
| rs8047395 | chr16:53798523 | A | G | Vascular or heart problems diagnosed by doctor: none of the above | UKBB | -0.006508 | 4.88E-09 | 336683 | risk diff |
| rs11713193 | chr3:49924424 | A | G | Wheeze or whistling in the chest in last year | UKBB | 0.006833 | 6.41E-12 | 331257 | risk diff |
| rs12705977 | chr7:114332246 | G | T | Wheeze or whistling in the chest in last year | UKBB | -0.005659 | 1.39E-08 | 331257 | risk diff |
| rs12964689 | chr18:21116998 | A | G | White blood cell count | 27863252 | -0.01959 | 4.53E-08 | 173480 | - |
| rs9267677 | chr6:31892641 | C | T | White blood cell count | 27863252 | 0.03623 | 2.6E-09 | 173480 | - |
| ^1^24 SNPs were identified as significantly different from the overall IVW estimate for effect of BMI on years of schooling using a Bonferroni-corrected threshold of 5.29E-5. Genome-wide significant associations (p<5x10-8) of these SNPs with other traits were identified using PhenoScanner v2: <http://www.phenoscanner.medschl.cam.ac.uk/>. The following SNPs were identified as outliers: rs10811901, rs10840606, rs11611246, rs11713193, rs11855853, rs12705977, rs12964689, rs1402025 rs1452075, rs1503526, rs16903285, rs2293605, rs2733287, rs329122, rs4916229, rs6449532, rs7334078, rs8047395, rs901630, rs903959, rs9267677, rs9816226, rs9817583, rs9965170. Note: for rs9817583, PhenoScanner identified no genome-wide significant associations | | | | | | | | | |
